# Imidazolyl‐Substituted Benzo‐ and Naphthodithiophenes as Precursors for the Synthesis of Transient Open‐Shell Quinoids

**DOI:** 10.1002/open.202300003

**Published:** 2023-01-26

**Authors:** Peng Hou, Sebastian Peschtrich, Wolfram Feuerstein, Roland Schoch, Stephan Hohloch, Frank Breher, Jan Paradies

**Affiliations:** ^1^ Chemistry Department Paderborn University Warburger Strasse 100 33098 Paderborn Germany; ^2^ Institute of Inorganic Chemistry Karlsruhe Institute of Technology (KIT) Engesserstraße 15 76131 Karlsruhe Germany; ^3^ Department of General, Inorganic and Theoretical Chemistry University of Innsbruck Innrain 80–82 6020 Innsbruck Austria

**Keywords:** density functional theory, heteroacene, quinoid, thiophene, UV/vis spectroscopy

## Abstract

The synthesis of three novel imidazolyl‐substituted sulfur‐containing heteroacenes is reported. These heteroacenes consisting of annelated benzo‐ and naphthothiophenes serve as precursors for the generation of open‐shell quinoid heteroacenes by oxidation with alkaline ferric cyanide. Spectroscopic and computational experiments support the formation of reactive open‐shell quinoids, which, however, quickly produce paramagnetic polymeric material.

## Graphical abstract

**A series of sulfur‐containing heteroacenes** were synthesized and utilized as precursors to imidazolylidene‐substituted quinoids. The transiently formed quinoids exhibit small singlet‐triplet gaps and exist as open‐shell molecules, as evidenced by EPR spectroscopy and quantum chemical calculations.

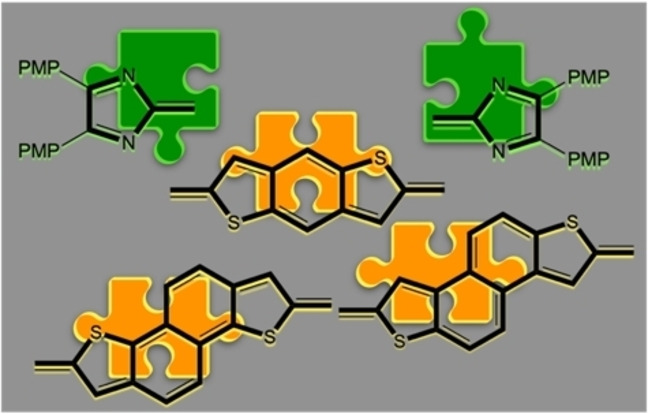

## Introduction

**Scheme 1:**
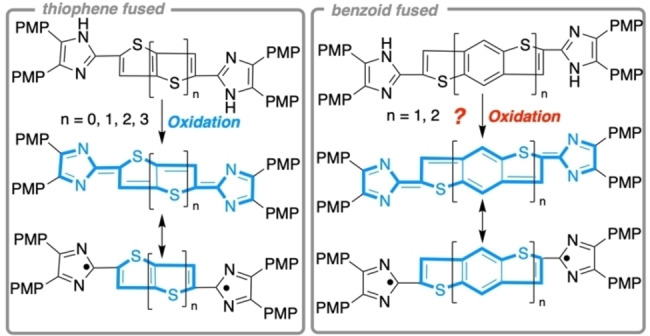
Sulfur‐containing heteroacenes and quinoid resonance structures.

The efficient harvesting of solar light is important for energy conversion technology. Against this background, organic solar cells and dye‐sensitized solar cells have attracted significant attention due to their potential of harvesting a broader spectrum of the sunlight, leading to improved efficiencies compared to non‐organic congeners.^[1]^ Quinoid organic structures have found use in solar cell applications due to their ability to absorb in the near infrared (NIR), thus increasing the bandwidth of wavelengths accessible for energy conversion.^[2]^ Therefore, the development of novel quinoid structures with NIR‐absorption properties has become a central interest.^[3]^ Their optical and electronic properties are highly dependent on their molecular structure, and the determination of structure‐property relationships is important for the tailored synthesis of materials for specific applications.^[4]^ Recently, we investigated the impact of thiophene annelation on the NIR absorbance of quinoid heteroacenes (Scheme 1, left).^[5]^ Expectedly, the increase of thiophene subunits led to a blueshift in the NIR absorption of these stable quinoids. At the same time, the singlet‐triplet gap decreased such that thermally accessible open‐shell states became available. To establish a more profound understanding of the impact of the carbocycle on the quinoid formation, in the present study, we describe the synthesis and spectroscopic as well as theoretical investigations of benzo and naphtho subunits fused to thiophene‐annelated quinoid heteroacenes.

## Results and Discussion

### Synthesis

**Figure 1:**
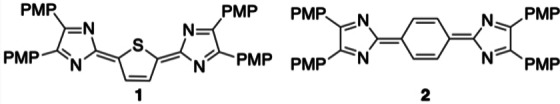
Stable thiophene‐ (**1**) and benzo‐ (**2**) derived quinoids as reference structures (PMP=4‐methoxyphenyl).

For comparison, the thiopheno and benzo derivatives **1** ^[6]^ and **2** ^[5]^ were synthesized according to the literature references in 88 % and 74 % yields, respectively (Figure 1, see Supporting Information for details).

**Synthesis of the oxidation precursors**: The position of the heteroatom in heteroacenes has significant impact on the optoelectronic properties. Thus, we targeted the synthesis of the isomerically pure benzo and naphtho derivatives by the double domino C−S cross‐coupling/5‐*endo*‐dig cyclization reaction developed earlier in our group.[^5^, ^7^]

**Scheme 2:**
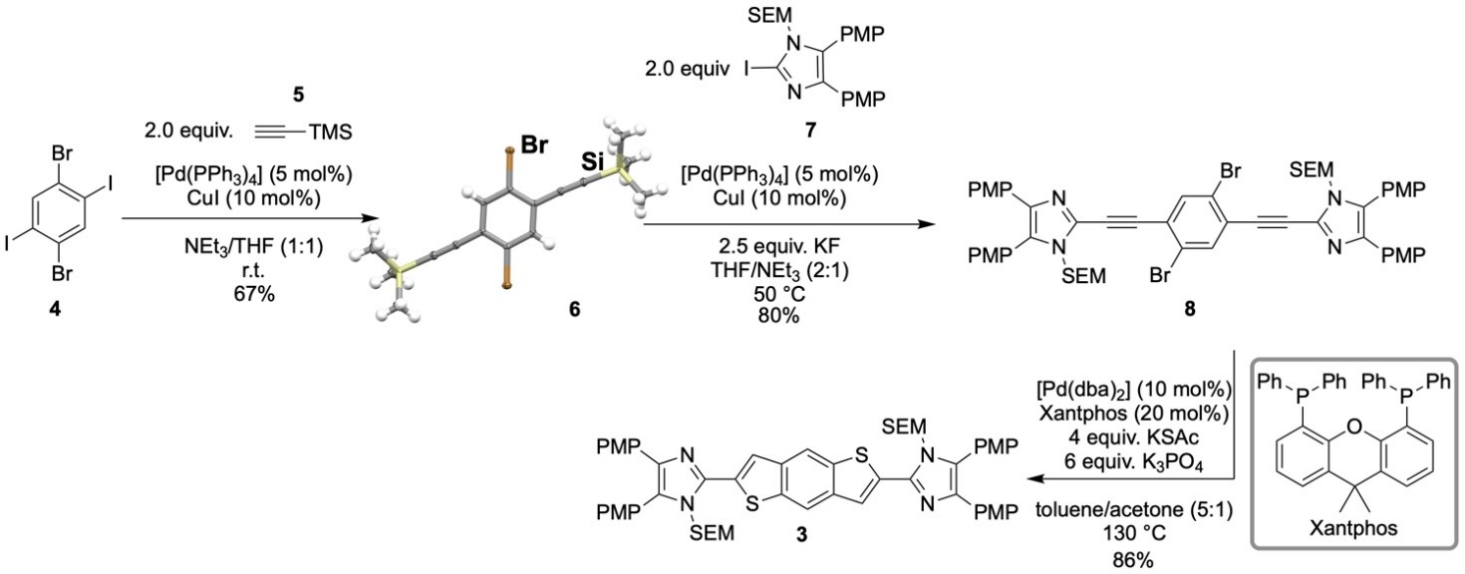
Synthesis of the benzo[1,2‐*b*:4,5‐*b′*]dithiophene oxidation precursor (PMP=4‐methoxyphenyl; SEM=trimethylsilylethoxymethyl).

The synthesis of **3** commenced with the double Sonogashira coupling of 1,4‐dibromo‐2,5‐diiodobenzene (**4**) with trimethylsilyl acetylene (**5**), providing the TMS‐protected product **6** in 67 % yield (Scheme 2). The two imidazole units were installed by in situ removal of the TMS group from **6** with potassium fluoride in the presence of the SEM‐protected iodoimidazole **7** (SEM=trimethylsilylethoxymethyl), 5 mol % of the palladium catalyst [Pd(PPh_3_)_4_] and 10 mol % copper(I) iodide. The cyclization precursor **8** was obtained as a yellow solid in 80 % yield. The SEM protection of the imidazole moieties was necessary during the whole process because of the poor solubility of the free amines. The double C−S cross‐coupling/cyclization sequence using potassium thioacetate as H_2_S surrogate[^7c^, ^7f^] was initiated by [Pd(dba)_2_] and Xantphos. This ring closing reaction provided isomerically pure **3** as a dark orange solid in 86 % yield.

**Scheme 3:**
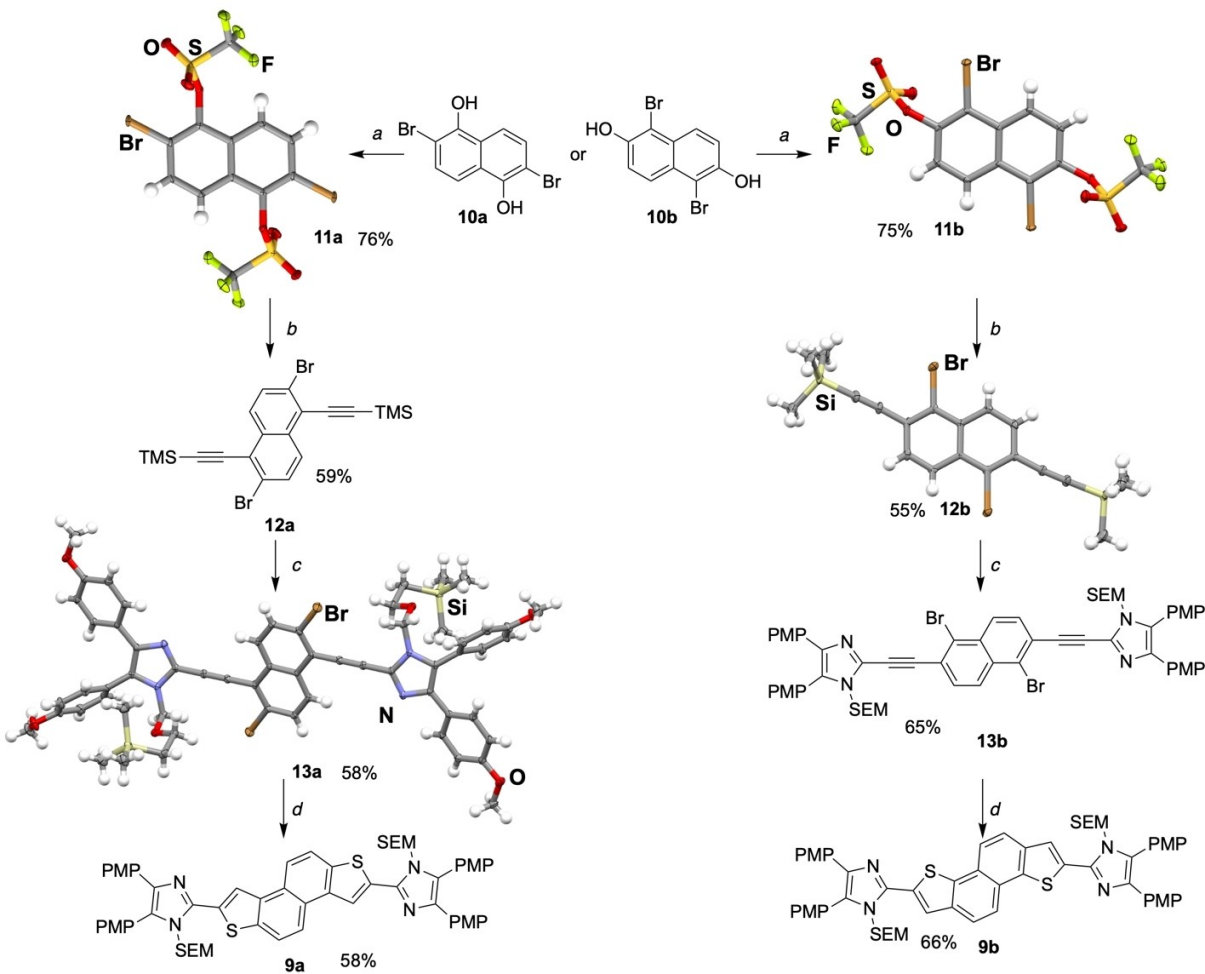
Synthesis of quinoid derivatives **10 a** and **10 b** (PMP=4‐methoxyphenyl; a) 5.5 equiv. pyridine, 2.1 equiv. Tf_2_O, CH_2_Cl_2_, 0 °C to r.t.,10 % HCl; b) 2.0 equiv. TMS‐acetylene (**5**), [Pd(PPh_3_)_4_] (5 mol %), CuI (10 mol %), HN*i*Pr_2_/THF (1 : 1), r.t.; c) 2.0 equiv. **7**, [Pd(PPh_3_)_4_] (5 mol %), CuI (10 mol %), 2.5 equiv. KF, THF/NEt_3_ (2 : 1), 50 °C; d) [Pd(dba)_2_] (10 mol %), Xantphos (20 mol %), 4.0 equiv. KSAc, 6.0 equiv. K_3_PO_4_, toluene/acetone (5 : 1), 130 °C (SEM=trimethylsilylethoxymethyl).

Next, we pursued a similar strategy for the synthesis of the two naphtho derivatives **9 a** and **9 b** as oxidation precursors (Scheme 3). The quadruple‐substituted naphthalene compounds **10 a** and **10 b** were necessary for the regioselective double Sonogashira coupling. Bromine and a triflate group turned out to work best for ensuring sufficient regioselectivity, and the Sonogashira coupling proceeded with excellent regiocontrol (Scheme 3). This allowed the synthesis of the orthogonally activated coupling partners **11 a** and **11 b** in 76 % and 75 % yield, respectively. The formation of the triflates was unambiguously established by X‐ray diffraction analysis. The regioselective Sonogashira coupling of **11 a** and **11 b** with TMS‐acetylene was achieved in 59 % and 55 % yield, respectively, in the presence of 5 mol % [Pd(PPh_3_)_4_] and 10 mol % CuI in a mixture of HN*i*Pr_2_/THF (1 : 1 ratio). The regioselectivity of the coupling reaction was further supported by the molecular structure determination of **12 b** by X‐ray diffraction analysis. The incorporation of the two imidazole units was accomplished by in situ deprotection of the TMS groups of **12 a** and **12 b** with potassium fluoride in the presence of the orthogonally SEM‐protected iodoimidazole **7**, 5 mol % of [Pd(PPh_3_)_4_] and 10 mol % copper(I) iodide. The products **13 a** and **13 b** were both obtained as yellow solids in 58 % and 65 % yield, respectively. The molecular structure of **13 a** was determined by X‐ray diffraction analysis, confirming the formation of the internal alkyne. The C−S cross‐coupling/cyclization sequence was initiated with potassium thioacetate as H_2_S surrogate and [Pd(dba)_2_]/Xantphos as precatalyst (10 mol %, 1 : 2 ratio). The SEM‐protected quinoid precursors **9 a** and **9 b** were obtained as dark orange solids in 58 % and 66 % yield, respectively. The successful cyclization was confirmed by ^1^H and ^13^C NMR spectroscopy. A new resonance signal in the aromatic region at δ=8.00 ppm (**9 a**) and 8.51 ppm (**9 b**) in the ^1^H NMR spectra was observed, corresponding to the thiophene C−H group.

**Scheme 4:**
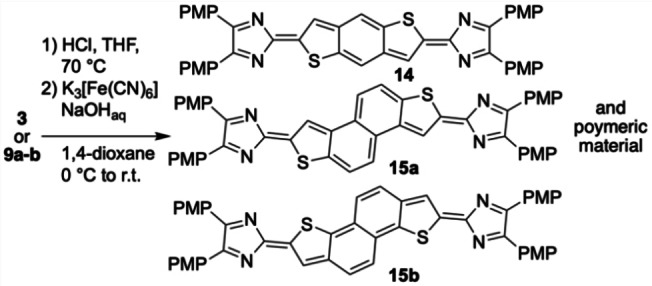
Reaction of **3**, **9 a** and **9 b** with K_3_[Fe(CN)_6_] after SEM‐deprotection.

**Oxidation attempts**: Finally, the oxidation of the heteroacenes **3**, **9 a** and **9 b** was investigated and required the SEM deprotection. This was achieved by the reaction with excess of aqueous HCl in THF and yielded the deprotected heteroacenes as yellow solids. The low solubility of the materials impeded further characterization by NMR spectroscopy and the material was carried through the oxidation step with ferric cyanide in alkaline aqueous media, upon which the formerly brown/orange suspension turned blue/black within seconds (Scheme 4).[^6^, ^8^]

Despite of the pronounced color change, no unambiguous evidence for the successful formation of the quinoids **14** and **15 a**–**b** could be found (see below for UV/vis spectroscopy and computational analysis). The obtained materials were insoluble in common solvent, preventing the analysis by NMR spectroscopy. However, ESI mass spectrometry in positive channel of a low‐concentration solution of **14** in acetonitrile/dichloromethane somewhat supports the formation of the quinoid product by the detection of m/z=745.1985, corresponding to the [M+H]^+^ ion. Additionally, a mass peak of m/z=746.1816 was detected which corresponds the [M]^+^ ion of the deprotected oxidation precursor as contamination. Likewise, the oxidation of the bis(imidazolyl) derivatives **9 a** and **9 b** yielded insoluble materials and did not allow the characterization by NMR spectroscopy. Again, ESI mass spectrometry in positive channel of low‐concentrated solutions in acetonitrile/dichloromethane supports the formation of the quinoid products **15 a**–**b** by the detection of the m/z=795.2094 and 795.2115 signals corresponding to the respective [M+H]^+^ species. Neither ESI nor MALDI mass spectrometric investigations supported the formation of oligomeric or polymeric material. Thus, the molecular structure of the obtained material remains inconclusive and presumably small amounts of the quinoids are present.

### Spectroscopic investigation

**Figure 2:**
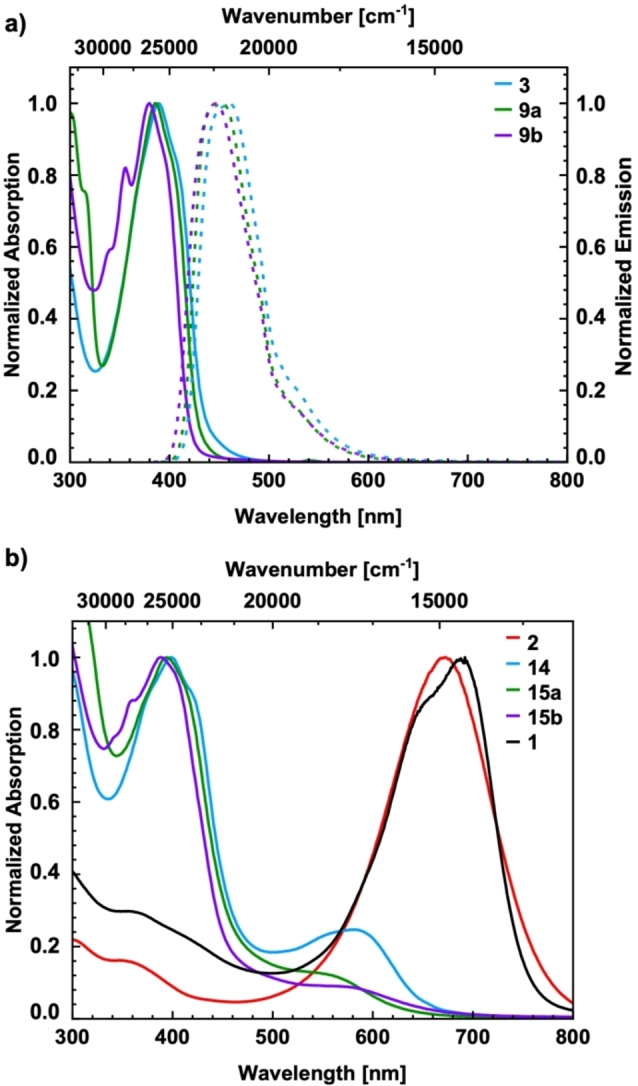
a) UV/vis spectroscopic data (solid lines) and fluorescence spectroscopic data (dashed lines) of the SEM‐protected bisimidazole derivatives **3**, **9 a** and **9 b**; b) UV/vis spectroscopic data after oxidation to **14**, **15 a** and **15 b** (UV/vis data of **1** and **2** included for comparison^[5]^).

The synthesized bisimidazole derivatives and the quinoid materials were examined by UV/vis and fluorescence spectroscopy. Spectra were recorded from 12.5–15.8 μm solutions in CH_2_Cl_2_ at 25 °C. The spectral results are summarized in Figure 2 and Table 1. The absorption maxima of the three bisimidazole derivatives **3**, **9 a** and **9 b** are in a similar range between 369 and 389 nm. As expected, with increasing size of the heterocyclic core, the absorption maximum is redshifted. The samples were irradiated at the λ_max_ edge, and the emission spectra were recorded. The Stokes shift of 0.47–0.50 eV indicates significant differences of geometric and electronic structure between the ground and the excited state. The material of the oxidation reactions was investigated by UV/vis and fluorescence spectroscopy, although it must be noted that the material is contaminated by the oxidation precursor.

**Table 1:** Summarized spectroscopic data of thioacenes and quinoid thioacenes.

Compound	λ_max_ (CH_2_Cl_2_) [nm]	λ_max_ (CH_2_Cl_2_) [eV]	Log ϵ (λ_max_) [1/(m⋅cm)]	λ_max‐FL_ (CH_2_Cl_2_) [nm]	λ_max‐FL_ (CH_2_Cl_2_) [eV]
**3**	389	3.19	4.87	461	2.69
**9 a**	386	3.21	4.98	453	2.74
**9 b**	380	3.26	4.94	447	2.77
**1**	688	1.80	4.96	–	–
**2**	678	1.83	4.95	–	–
**14**	398	3.12	4.69	–	–
**15 a**	394	3.15	4.78	–	–
**15 b**	388	3.20	4.76	–	–

Thus, the clean formation of the oxidation products **14**, **15 a** and **15 b** is also not fully supported by UV/vis spectroscopy. As reported previously,^[5]^ a significant red shift compared to the reduced species is expected and can be observed for quinoids **1** and **2** with an absorption maximum around 680 nm. When the benzene core is extended by two thiophene units (**14**), only a small band at higher wavelengths, at 581 nm, is visible. For the even more extended cores of **15 a** and **15 b**, this band becomes less pronounced. The UV/vis difference spectra (see Supporting Information) show significantly different absorption characteristics compared to the corresponding oxidation precursors. The λ_max_ band is slightly redshifted and significantly broadened, indicating different species than the oxidation precursors. The fluorescence properties of the oxidation products were also investigated, but in line with our earlier studies, the quinoids displayed no fluorescence.

Open‐shell quinoids tend to form dimeric, oligomeric or polymeric products with loss of their NIR absorption properties. Thus, in line with our previous results, we anticipate the transient formation of the quinoids and subsequent oligo‐ and polymerization.[^3d^, ^9^] This is supported by the observation of EPR‐active species (X‐band) at room temperature and at 90 K in solution and in the solid state (see Supporting Information). It has been frequently observed that for related diradicals of this type,^[10]^ a doublet EPR signal is observed, which corresponds to a monoradical species. This finding is in line with literature reports on the so‐called “diradical paradox”.^[11]^ Doublet EPR signatures from diradicals may result from self‐aggregation, monoradical impurities, etc. Such doublet signals may overlay the weak intensity of a triplet signal which, on first sight, would be expected for the spectra of triplet diradicals, if the latter exist in a thermal equilibrium with a closed‐shell molecule or open‐shell singlet diradical.^[12]^ We found that **14**, **15 a** and **15 b** exhibit a doublet EPR signature at 90 K and at room temperature. The quinoid **2** was EPR‐active only at room temperature, so a thermal excitation to the open‐shell triplet can be assumed. However, more information could not be extracted from the EPR spectroscopic experiments. Therefore, it must be assumed that the quinoids are highly reactive species which undergo consecutive reactions, restoring the non‐quinoid resonance form with loss of NIR absorption, which is reported for similar imidazolylidene derivatives.^[9]^ The open‐shell nature of the quinoid **2** and of the presumably transiently generated quinoids **14**, **15 a** and **15 b** is furthermore supported by quantum chemical calculations.

### Computational investigations

**Figure 3:**
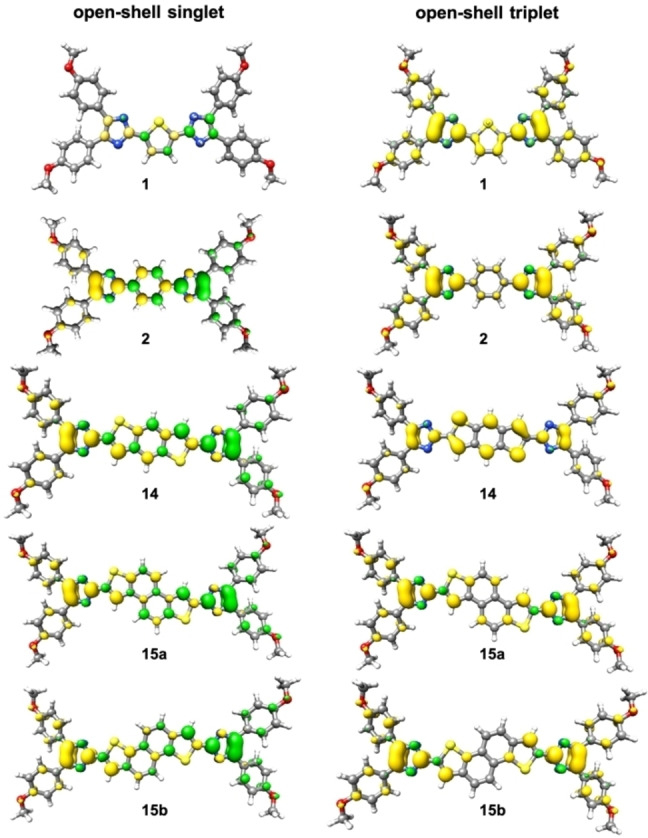
Spin densities of the open‐shell singlet (left) and open‐shell triplet (right) optimized geometries (PBE0/def2‐TZVP; isovalue ±0.0036 bohr^−3^).

The electronic structures of the quinoids were investigated by density functional theory (DFT) and by complete active space self‐consistent field (CASSCF) calculations as implemented in the ORCA 5.0.3 package.^[13]^ The geometries were optimized using the PBE0^[14]^ functional in combination with the def2‐TZVP^[15]^ basis set. For geometries with different orbital occupations, we conducted optimizations using the Kohn–Sham formalism for closed‐shell (RKS singlet), open‐shell triplet (UKS triplet) and open‐shell broken‐symmetry singlet (UKS singlet). Energies and details are summarized in Table 2. For comparison, the thiophene (**1**) and the benzo (**2**) derivatives were included. It was found that the open‐shell singlet is energetically more favored than the closed‐shell singlet. However, the energy and geometry of the closed‐shell singlet of **1** are almost identical to those of the open‐shell singlet. The closed‐shell benzo system **2** is significantly destabilized in comparison to the thiophene derivative **1**. This can be rationalized by the increased resonance energy gained from aromatization, stabilizing the open‐shell singlet. This is also reflected in the increased LUMO occupation of 41 % computed from a CASSCF(2,2) calculation.^[16]^ The spin densities of the UKS triplet and UKS singlet geometries are depicted in Figure 3 and are in line with the assumption of unpaired electrons in a diradical resonance structure.

**Table 2:** Relative energies (PBE0/def2‐TZVP) of closed‐shell and open‐shell states in kJ mol^−1^ (value in parentheses corresponds to the *S* ^2^ expectation value).

Compound	RKS	UKS triplet	UKS singlet	LUMO occ.^[a]^	*N_FOD_*
**1**[25]	0.1	42.5 (2.033)	0.0 (0.090)	0.15	1.304
**2**[25]	6.0	26.2 (2.037)	0.0 (0.623)	0.41	1.544
**14**	15.0	14.1 (2.025)	0.0 (0.867)	0.71	1.811
**15 a**	31.5	6.2 (2.045)	0.0 (0.99)	0.79	2.157
**15 b**	45.9	2.5 (2.051)	0.0 (1.043)	0.94	2.228

[a] LUMO occupation obtained from CASSCF(2,2)/def2‐TZVP calculations on the PBE0/def2‐TZVP‐optimized UKS singlet geometries; [b] *N_FOD_* obtained from TPSS/def2‐TZVP calculation on the PBE0/def2‐TZVP‐optimized UKS singlet geometries with a smearing temperature of 5000 K.

In line with our earlier findings,^[5]^ the spin density is majorly localized at the imidazole moieties. The molecule **14** consists of two thiophene rings annelated to the central benzene. This significantly reduces the triplet energy and destabilizes the closed‐shell state. This trend is also observed for the two isomeric naphtho derivatives **15 a** and **15 b**. As indicated by the *S* ^2^ expectation values of the UKS singlets, these states suffer from significant spin contamination. This is further supported by the fractional occupation number weighted electron density (FOD) analysis,^[17]^ which reveals increasing static electron correlation, as indicated by the larger *N_FOD_* occupation number. Although the open‐shell singlet energies are overstabilized, as reported earlier,^[18]^ and require spin projection for quantitative analysis, a qualitative trend can be clearly identified. The increase of the central aromatic carbon framework leads to increasing LUMO occupation, resulting in biradical character (compare **14** and **15 a**). Also, the connectivity of the annelation has an influence on the LUMO occupation. The calculated LUMO occupation of the naphtho[2,1‐*b*:6,5‐*b′*]dithiophene isomer **15 a** is 15 % lower than that of the corresponding naphtho[1,2‐*b*:5,6‐*b′*]dithiophene derivative **15 b**. These results fully support the formation of reactive open‐shell species and are in line with the observed EPR signals. However, the TD‐DFT spectra of the singlet or the triplet states of **14**, **15 a** or **15 b** could not be matched to the recorded spectra in Figure 2.

## Conclusion

We have synthesized three new bis(imidazolyl)‐substituted sulfur‐containing heteroacenes featuring benzo and naphtho subunits through palladium‐catalyzed C−S cross‐coupling reactions. The oxidation of these heteroacenes leads to reactive open‐shell structures which quickly undergo polymerization. Our studies revealed that the benzo and naphtho derivatives exist as biradicals. With increasing size of the carbocyclic unit, the open‐shell triplet state is only 6.2 to 2.5 kJ mol^−1^ less stabilized than the singlet state, which accounts for the formation of polymeric structures accompanied with loss of diradical character and NIR absorption.

Taking these findings and our earlier report on sulfur‐rich quinoid heteroacenes into account, a strategy for the concise synthesis of open‐shell quinoid molecules can be outlined. For open‐shell singlet quinoids, the incorporation of heterocycles like thiophenes to a high degree is advised. This results in reduced stabilization energy though the formation of a six‐electron π‐system,^[19]^ in other words, reduced aromaticity, which in turn facilitates cross‐conjugation. In contrast, when open‐shell triplet structures are of interest, carbon‐rich annelated benzo systems should be targeted, due to the higher stabilization energy of the aromatic state. These fundamental considerations will help in the rational design of new structures with interesting optoelectronic and magnetic properties.

## Experimental Section

Detailed experimental procedures and analytical data can be found free of charge in the Supporting Information. Crystallographic data have been deposited at the Cambridge Crystallographic Data Centre. Deposition Numbers 2214715 (for **11 b**), 2214716, (for **12 b**) 2214717 (for **13 a**), 2214718 (for **11 a**) and 2221938 (for **6**) contain the supplementary crystallographic data for this paper. These data are provided free of charge by the joint Cambridge Crystallographic Data Centre and Fachinformationszentrum Karlsruhe Access Structures service.

## Conflict of interest

The authors declare no conflict of interest.

1

## Supporting information

As a service to our authors and readers, this journal provides supporting information supplied by the authors. Such materials are peer reviewed and may be re‐organized for online delivery, but are not copy‐edited or typeset. Technical support issues arising from supporting information (other than missing files) should be addressed to the authors.

Supporting Information

## Data Availability

The data that support the findings of this study are available in the supplementary material of this article.
