# Supplementary material for: Imidazolyl‐Substituted Benzo‐ and Naphthodithiophenes as Precursors for the Synthesis of Transient Open‐Shell Quinoids
Source: ChemistryOpen. 2023 Jan 26;12(11):e202300003. doi: 10.1002/open.202300003 (PMC10661821; doi:10.1002/open.202300003)
Supplement: Supplementary file 1 — Supporting Information [file OPEN-12-e202300003-s001.pdf]

ChemistryOpen

Supporting Information

Imidazolyl-Substituted Benzo- and Naphthodithiophenes as Precursors for the Synthesis of Transient Open-Shell Quinoids

Peng Hou, Sebastian Peschtrich, Wolfram Feuerstein, Roland Schoch, Stephan Hohloch, Frank Breher, and Jan Paradies*

CONTENTS

1. GENERAL INFORMATION 4

1.1. Chemical materials and synthesis techniques 4

1.2. Characterization methods 4

2. EXPERIMENTAL SECTION 7

2.1. Synthesis of 2,5-bis(4,5-bis(4-methoxyphenyl)-2H-imidazol-2-ylidene)-2,5-dihydrothiophene (1) 7

2.2. Synthesis of 3,6-bis(4,5-bis(4-methoxyphenyl)-2H-imidazol-2-ylidene)cyclohexa-1,4-diene (2) 8

2.3. Preparing the core precursors 9

2.3.1. Synthesis of ((2,5-dibromo-1,4-phenylene)bis(ethyne-2,1-diyl))bis(trimethylsilane) (6).... 9

2.3.2. General Procedure of Trifluoromethanesulfonate-Substituted Naphthalen 9

2.3.2.1. 2,6-dibromonaphthalene-1,5-diyl bis(trifluoromethanesulfonate) (11a)...................... 10

2.3.2.2. 1,5-dibromonaphthalene-2,6-diyl bis(trifluoromethanesulfonate) (11b)...................... 10

2.3.3. General Procedure of TMS-Acetylene-Substituted Naphthalene 11

2.3.3.1. ((2,6-dibromonaphthalene-1,5-diyl)bis(ethyne-2,1-diyl))bis(trimethylsilane) (12a)......11

2.3.3.2. ((1,5-dibromonaphthalene-2,6-diyl)bis(ethyne-2,1-diyl))bis(trimethylsilane) (12b)......11

2.4. Preparing imidazole derivatives 12

2.4.1. Synthesis of 4,5-bis(4-methoxyphenyl)-1*H*-imidazole (SI3) 12

2.4.2. Synthesis of 4,5-bis(4-methoxyphenyl)-1-((2-(trimethylsilyl)ethoxy)methyl)-1H-imidazole (SI4) 13

2.4.3. 2-iodo-4,5-bis(4-methoxyphenyl)-1-((2-(trimethylsilyl)ethoxy)methyl)-1*H*-imidazole (7) . 14

2.5. Bis(alkyne)-imidazol precursor by Sonogashira cross-coupling 15

2.5.1. 2,2'-((2,5-dibromo-1,4-phenylene)bis(ethyne-2,1-diyl))bis(4,5-bis(4-methoxyphenyl)-1-((2-(trimethylsilyl)ethoxy)methyl)-1H-imidazole) (8) 15

2.5.2. 2,2'-((2,6-dibromonaphthalene-1,5-diyl)bis(ethyne-2,1-diyl))bis(4,5-bis(4-methoxyphenyl) -1-((2-(trimethylsilyl)ethoxy)methyl)-1H-imidazole) (13a) 16

2.5.3. 2,2'-((1,5-dibromonaphthalene-2,6-diyl)bis(ethyne-2,1-diyl))bis(4,5-bis(4-methoxyphenyl) -1-((2-(trimethylsilyl)ethoxy)methyl)-1H-imidazole) (13b) 17

2.6. Annulation to heterocacene by Pd-catalyzed C-S-cross-coupling 18
2.6.1. 2,6-bis(4,5-bis(4-methoxyphenyl)-1-((2-(trimethylsilyl)ethoxy)methyl)-1H-imidazol-2-yl)benzo[1,2-b:4,5-b']dithiophene (3) 18
2.6.2. 2,7-bis(4,5-bis(4-methoxyphenyl)-1-((2-(trimethylsilyl)ethoxy)methyl)-1H-imidazol-2-yl)naphtho[2,1-b:6,5-b']dithiophene (14a) 19
2.6.3. 2,7-bis(4,5-bis(4-methoxyphenyl)-1-((2-(trimethylsilyl)ethoxy)methyl)-1H-imidazol-2-yl)naphtho[1,2-b:5,6-b']dithiophene (14b) 19
2.7. Deprotection of SEM-group and oxidation of deprotected precursors with Fe^{3+} to quinoidal form 20
2.7.1. General deprotection procedure of SEM group 20
2.7.1.1. 2,6-bis(4,5-bis(4-methoxyphenyl)-1H-imidazol-2-yl)benzo[1,2-b:4,5-b']dithiophene (SI5) 21
2.7.1.2. 2,7-bis(4,5-bis(4-methoxyphenyl)-1H-imidazol-2-yl)naphtho[2,1-b:6,5-b']dithiophene (SI6) 21
2.7.1.3. 2,7-bis(4,5-bis(4-methoxyphenyl)-1H-imidazol-2-yl)naphtho[1,2-b:5,6-b']dithiophene (SI7) 22
2.7.2. General procedure for oxidation of precursor to quinoids 22
2.7.2.1. 2,6-bis(4,5-bis(4-methoxyphenyl)-2H-imidazol-2-ylidene)-2,6-dihydrobenzo[1,2-b:4,5-b']dithiophene (9) 22
2.7.2.2. 2,7-bis(4,5-bis(4-methoxyphenyl)-2H-imidazol-2-ylidene)-2,7-dihydronaphtho[2,1-b:6,5-b']dithiophene (10a) 23
2.7.2.3. 2,7-bis(4,5-bis(4-methoxyphenyl)-2H-imidazol-2-ylidene)-2,7-dihydronaphtho[1,2-b:5,6-b']dithiophene (10b) 23
3. CHARACTERIZATION 24
3.1. NMR-spectra 24
3.2. Mass spectra 42
3.3. UV-Vis difference spectra 54
3.4. EPR measurement 55
4. REFERENCES 59

1. General Information

1.1. Chemical materials and synthesis techniques

All commercially available starting materials, reagents and solvents were used as received without further purification unless specially mentioned otherwise. The chemical compounds were purchased from *Sigma Aldrich*, *ABCR*, *Alfa Aesar*, *TCI Carl Roth and Acros*. O_2- and moisture-free solvents were either used as commercially received or pre-dried as follow: Dichloromethane (DCM) – distillation with calcium hydride as desiccant, Tetrahydrofurane (THF) – distillation with anhydrous calcium dichloride as desiccant, toluene – distillation with anhydrous calcium dichloride as desiccant, diethyl ether – distillation with anhydrous calcium dichloride as desiccant, acetone – bubbling with argon for 10 mins with molecular sieves as desiccant or lyophilization with schlenk line technique for 3 circles and molecular sieves as desiccant. Molecular sieves were pre-dried in vacuum at 140 °C for 24 h and stored in an oven at 120 °C or in an argon filled glovebox. Solutions of inorganic salts or aqueous soluble substances were used as solution in distilled water as solvent.

All preparations involving air and/or moisture sensitive compounds were carried out in oven dried (120 °C) glassware (round-bottom-flask or vial). Reactions using air and/or moisture sensitive compounds were performed under dry O_2-free Argon atmosphere with schlenk line techniques or in an argon filled glovebox by *Glovebox System*. Liquids were transferred with a plastic syringe connected to a steel cannula (120 mm, Ø0.8 mm). The following mixtures were employed for temperature control below room temperature: 0 °C-water/ice, −10 °C to 0 °C-ice/NaCl, −78 °C to −10 °C-acetone/liquid nitrogen. The monitoring of the reactions was indicated on thin-layer-chromatography using silica gel on aluminum sheet (TCL silica gel 60, F_{254}). The purifications by flash column chromatography were carried out on silica gel under constant excess pressure of 0.8 bar of nitrogen gas. (Si 60). Eluents for flash column chromatography were pre-distilled under reduced pressure with a rotary evaporator (water bath, temperature at 60 °C). The rotary evaporator was also utilized for the removal of solvents and volatiles.

1.2. Characterization methods

Nuclear magnetic resonance spectroscopy (NMR)

^{1}H NMR spectra were recorded on a *Bruker Avance* 300 NMR-spectrometer (300 MHz), a *Bruker Avance* 500 NMR-spectrometer (500 MHz) or a *Bruker Ascent* 700 NMR-spectrometer (700 MHz) in

deuterated solvents. Chemical shifts are expressed in ppm (parts per million) referred to the residual proton and carbon signal of $CDCl_3$ (7.26 ppm for ^{1}H NMR, 77.16 ppm for ^{13}C NMR), DMSO-d_6 (2.50 ppm for ^{1}H NMR, 39.52 ppm for ^{13}C NMR), THF-d_8 (1.72 ppm, 3.58 ppm for ^{1}H NMR, 67.21 ppm, 25.31 ppm for ^{13}C NMR), toluol-d_6 (2.08 ppm, 6.97 ppm, 7.01 ppm, 7.09 ppm for ^{1}H NMR, 137.48 ppm, 128.87 ppm, 127.96 ppm, 125.12 ppm and 20.43 ppm for ^{13}C NMR). The description of ^{1}H NMR signals multiplicity involved are: s = singlet, d = doublet, t = triplet, q = quartet, b = broad, m = multiplet etc. The ^{1}H coupling constants are given in absolute values in Hertz (Hz). The data are reported as followed: chemical shift, multiplicity, integration, absolute values of coupling constant in Hz. ^{13}C NMR spectra were recorded on a *Bruker Avance* 500 NMR-spectrometer (500 MHz) or a *Bruker Ascent* 700 NMR-spectrometer (700 MHz) in deuterated solvents. All measurements performed by 500 MHz are at 303 K and all measurements performed by 700 MHz are at 298 K.

Mass spectrometry (MS)

The Mass-spectrometry was recorded on a *Finnigan* MAT 95 mass-spectrometer (ionization EI), a *Waters Synapt* 2G mass-spectrometer (ionization ESI). Molecular fragments are observed as mass-to-charge ratio (m/z) and the abbreviation [M] refers to neutral molecule while [M^+] refers to the molecule-ion.

Infrared spectroscopy (IR)

IR spectra were recorded from solids (ATR) on a *JASCO* FTIR 460 infrared spectrometer. The deposit of the absorption bands is given in wave numbers ($\tilde{v}$)in cm^{-1}. The indications of the absorption are categorized as follow: vs (very strong, < 20% transmission), s (strong, 20%-40% transmission), m (medium, 40%-60% transmission), w (weak, 60%-80% transmission) and vw (very weak, > 80% transmission).

Ultraviolet-visible light spectroscopy (UV/Vis)

UV/Vis spectra were recorded on an *Agilent Technologies* Cary 50 UV-VIS-spectrometer The measurements were performed in DCM or THF solutions of the respective compounds.

Fluorescence spectroscopy

Luminescence spectra were recorded on a *JASCO* FP-8300 fluorescence-spectrometer. The measurements were performed in DCM or THF solutions of the respective compounds.

Electron paramagnetic resonance spectroscopy (EPR)

EPR spectra were recorded using a Bruker EMXplus continuous wave (cw) X-Band spectrometer with nitrogen cooling for 100 K measurements. Samples were filled into fused silica glass tubes. Solvents were vacuum transferred to the samples and afterwards the sample tubes were sealed under vacuum. EPR spectra were analysed and simulated using the EasySpin MATLAB toolbox (Stoll, S. & Schweiger, A. EasySpin, a comprehensive software package for spectral simulation and analysis in EPR. J. Magn. Reson. 178, 42–55 (2006). MATLAB R2017a (9.2.0). The MathWorks (2017).)

X-ray single crystal diffraction

The presented X-ray single crystal data were collected on a *Bruker Venture D8* three-cycle diffractometer equipped with a Mo K_α μ-source (λ=0.71073 Å). Monochromatization of the radiation was obtained using *Incoatec* multilayer Montel optics and a Photon III area detector was used for data acquisition. All crystals were kept at 120 K during measurement.

Data processing was carried out using the *Bruker* APEX 3 software package: This includes SAINT for data integration and SADABS for a multi-scan absorption correction. Structure solution was obtained by direct methods and the refinement of the structures using full-matrix least squares method based on F^2 were achieved in SHELX.[SI1] All non-hydrogen-atoms were refined anisotropically and the hydrogen atom positions were refined at idealized positions riding on the carbon atoms with isotropic displacement parameters $U_{iso}(H)=1.2\ U_{eq}(C)$ and C-H bond lengths of 0.93-0.96 Å.

Crystallographic data have been deposited at the Cambridge Crystallographic Data Centre assigned to the deposition numbers **2214715-2214718** and **2221938**. Copies are available free of charge via www.ccdc.cam.ac.uk.

2. Experimental section

2.1. Synthesis of 2,5-bis(4,5-bis(4-methoxyphenyl)-2H-imidazol-2-ylidene)-2,5-dihydrothiophene (1)

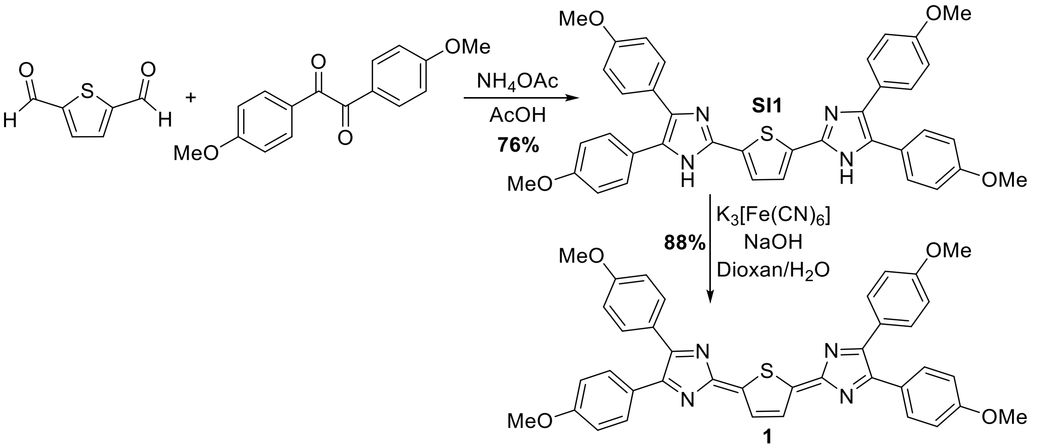

The quinoid was synthesized according to literature in 67% yield over two steps.[SI2]

SI1:

^{1}H NMR (300 MHz, DMSO): δ [ppm] = 12.66 (br 2H, H_{NH}), 7.86 (d, $^{3}J_{HH}$ = 8.79 Hz, 8H, H_{Ph}), 7.60 (s, 2H, $H_{thiophene}$), 7.14 (d, $^{3}J_{HH}$ = 8.84 Hz, 8H, H_{Ph}), 3.83 (s, 12H, H_{OCH3}).

MS (ESI) *m/z*: calc. $[C_{38}H_{33}N_4O_4S]^+$ = 641.2218, found: 641.2267.

1:

^{1}H NMR (300 MHz, $CDCl_3$): δ [ppm] = 8.30 (s, 1H, $H_{thiophene}$), 7.4 (dd, $^{3}J_{HH}$ = 14.68 Hz, 8H, H_{Ph}), 6.94 (dd, $^{3}J_{HH}$ = 8.84 Hz, 8H, H_{Ph}), 3.88 (s, 12H, H_{OCH3})

^{13}C NMR (125 MHz, $CDCl_3$): δ [ppm] = 162.1 (2C), 161.8 (2C), 138.7 (2C), 131.8 (4C), 131.5 (4C), 126.1 (2C), 114.2 (4C), 114.2 (4C), 55.6 (4C).

MS (ESI) *m/z*: calc. $[C_{38}H_{31}N_4O_4S]^+$ = 639.2061, found: 639.2205.

2.2. Synthesis of 3,6-bis(4,5-bis(4-methoxyphenyl)-2H-imidazol-2-ylidene)cyclohexa-1,4-diene (2)

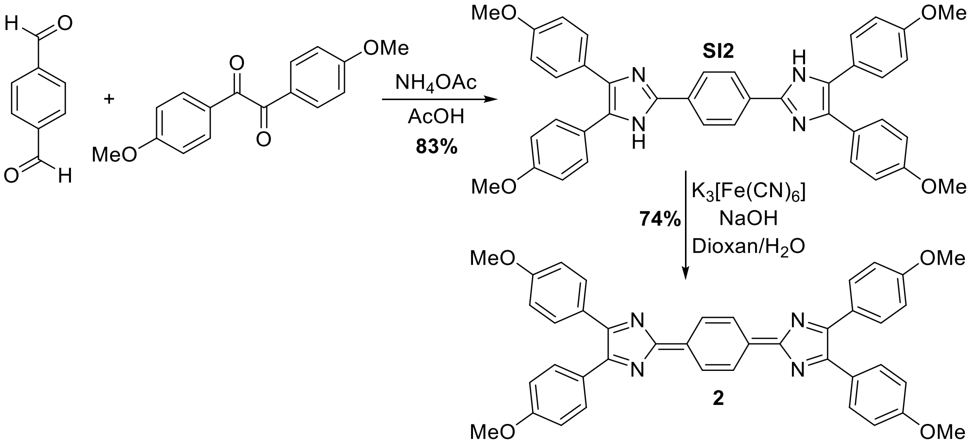

The quinoid was synthesized according to literature in 61% yield over two steps.[SI2

SI2:

^{1}H NMR (300 MHz, DMSO): δ [ppm] = 12.54 (s, 2H, H_{NH}), 8.14(s, 4H, H_{Ph}), 7.49 (d, $^{3}J_{HH}$ = 8.82 Hz, 4H, H_{Ph}), 7.44 (d, $^{3}J_{HH}$ = 8.82 Hz, 4H, H_{Ph}), 7.02 (d, $^{3}J_{HH}$ = 8.82 Hz, 4H, H_{Ph}), 6.89 (d, $^{3}J_{HH}$ = 8.93 Hz, 4H, H_{Ph}), 3.81 (s, 6H, H_{OCH3}), 3.76 (s, 6H, H_{OCH3}).

^{13}C NMR (125 MHz, DMSO): δ [ppm] = 131.7 (2C), 129.4 (4C), 127.9 (4C), 124.9 (4C), 114.5 (2C), 113.8 (4C), 113.4 (4C), 54.9 (4C), 54.7 (4C).

2:

^{1}H NMR (700 MHz, CDCl$_3$): δ [ppm] = 8.54 (s, 4H, H_{Ph}), 7.81 (d, $^{3}J_{HH}$ = 8.78 Hz, 8H, H_{Ph}), 6.97 (d, $^{3}J_{HH}$ = 8.79 Hz, 8H, H_{Ph}), 3.89 (s, 12H, H_{OMe}).

^{13}C NMR (176 MHz, CDCl$_3$): δ [ppm] = 166.54 (4C), 166.34 (2C), 162.21 (4C), 135.82 (2C), 132.04 (8C), 131.83 (4C), 126.67 (4C), 114.34 (8C), 55.69 (4C).

MS (ESI) *m/z*: calc. $[C_{38}H_{33}N_4O_4S]^+$ = 633.2497, found: 633.2504.

2.3. Preparing the core precursors

2.3.1. Synthesis of ((2,5-dibromo-1,4-phenylene)bis(ethyne-2,1-diyl))bis(trimethylsilane) (6)

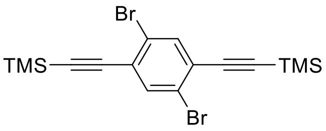

1,4-dibromo-2,5-diiodobenzene (1.46 g, 3.0 mmol, 1.0 equiv.) and CuI (57 mg, 0.3 mmol, 0.1 equiv.) were added to a Schlenk-flask. The flask was then degassed and backfilled with argon. $Pd(PPh_3)_4$ (173 mg, 0.15 mmol, 5% equiv.) was then added to the flask and degassed THF (15 ml) and TEA (15 ml) was injected via syringe. TMS-acetylene **5** (619 mg, 0.89 ml, 6.3 mmol, 2.1 equiv.) was added dropwise to the mixture. The reaction was stirred at ambient temperature overnight. Then the reaction mixture was poured into saturated NH_4Cl solution and extracted with DCM three times with 30 ml. The combined organic layer was dried over $MgSO_4$ and filtered. The filter was rinsed three times with 10 ml DCM. The collected organic layer was freed from volatiles under vacuum. The product was purified by column chromatography (SiO_2, pure CH) and obtained as white powder in 67% yield (0.86 g, 2.0 mmol).

^{1}H NMR (500 MHz, 303 K, $CDCl_3$): δ [ppm] = 0.27 (s, 9H, H_{TMS}); 7.67 (s, 2H, H_{Ar}).

^{13}C NMR (125 MHz, 303 K, $CDCl_3$): δ [ppm] = 136.6 (1C); 126.6 (1C); 123.9 (1C); 103.2 (1C); 101.5 (1C); -0.2 (3C).

MS (ESI) *m/z*: calc. [$C_{16}H_{19}Br_2Si_2$] = 427.9450, found: 427.9446.

IR: ῦ = 3090 (vw), 2958 (m), 2896 (m), 1557 (w), 1462 (m), 1247 (s), 1063 (m), 838

2.3.2. General Procedure of Trifluoromethanesulfonate-Substituted Naphthalene

2,6-dibromonaphthalene-1,5-diol (**10a**) or 1,5-dibromonaphthalene-2,6-diol (**10b**) was added to a flask which was degassed and backfilled with argon. Then 50 ml dried DCM was added to form a suspension. The suspension was cooled to 0 °C with an ice bath. Pyridine (5.5 equiv) was added to the suspension and Tf_2O (2.1 equiv) was slowly added dropwise to the mixture within one hour. The mixture was kept at 0 °C for one hour and stirred overnight at ambient temperature. Then 100 ml 10% aqueous HCl solution was added. The brown mixture was extracted with DCM three times with 30 ml, the organic layer was collected, dried over $MgSO_4$ and filtered. The filter was rinsed three times with 10 ml DCM.

The collected organic layer was freed from volatiles under vacuum. The crude product was purified by column chromatography and obtained as gray to light brown powder in 75% - 76% yield.

2.3.2.1. 2,6-dibromonaphthalene-1,5-diyl bis(trifluoromethanesulfonate) (11a)

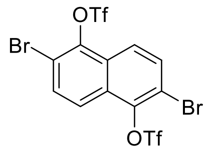

Starting from 2,6-dibromonaphthalene-1,5-diol (**10a**) after purification by column chromatography (SiO_2, CH:EE = 3:1), the product was obtained in 76% (2.2 g, 3.8 mmol) as light brown powder.

^{1}H NMR (500 MHz, 303 K, $CDCl_3$): δ [ppm] = 8.02 (d, $^3J_{HH}$ = 9.07 Hz, 2H, $H_{Naphtho}$); 7.88 (d, $^3J_{HH}$ = 9.04 Hz, 2H, $H_{Naphtho}$).

^{13}C NMR (125 MHz, 303 K, $CDCl_3$): δ [ppm] = 142.43 (2C); 133.00 (2C); 128.50 (2C); 122.76 (2C); 119.78 (1C); 117.22 (1C); 116.48 (2C).

HRMS (EI) *m/z*: calc. [$C_{12}H_4Br_2F_6O_6S_2$] = 581.7700, found: 581.7275.

IR: ṽ = 3098 (vw), 1586 (m), 1406 (s), 1220 (s), 1205 (s), 1129 (s), 933 (s), 853 (s), 817 (s), 786 (s), 749 (s), 694 (s), 619 (s).

2.3.2.2. 1,5-dibromonaphthalene-2,6-diyl bis(trifluoromethanesulfonate) (11b)

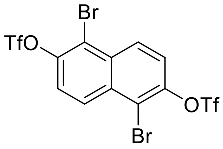

Starting from 1,5-dibromonaphthalene-2,6-diol (**10b**) after purification by column chromatography (SiO_2, CH:EE = 3:1), the product was obtained in 75% yield (2.8 g, 4.8 mmol).

^{1}H NMR (500 MHz, 303 K, $CDCl_3$): δ [ppm] = 8.46 (d, $^3J_{HH}$ = 9.32 Hz, 2H, $H_{Naphtho}$); 7.65 (d, $^3J_{HH}$ = 9.31 Hz, 2H, $H_{Naphtho}$).

^{13}C NMR (125 MHz, 303 K, $CDCl_3$): δ [ppm] = 146.5 (2C); 132.5 (2C); 129.9 (2C); 122.7 (2C); 119.9 (1C); 117.4 (1C); 116.6 (2C).

HRMS (EI) *m/z:* calc. [$C_{12}H_4Br_2F_6O_6S_2$] = 581.7700, found: 581.7690.

IR: ṽ = 1589 (w), 1491 (w), 1423 (m), 1363 (m), 1201 (m), 1136 (s), 962 (m), 838 (s), 813 (s), 728 (m), 626 (w).

2.3.3. General Procedure of TMS-Acetylene-Substituted Naphthalene

11a or **11b** and CuI (0.1 equiv.) were added to a Schlenk-Flask which was degassed and backfilled with argon. $Pd(PPh_3)_4$ (0.05 equiv.) was added to the vessel and degassed THF (3.5 ml/mmol) and DIPA (3.5 ml/mmol) were injected via syringe. TMS-acetylene (2.0 equiv.) was added dropwise to the rection mixture. The reaction was stirred at room temperature overnight. Then the reaction mixture was poured into saturated NH_4Cl solution and extracted with DCM three times (each 30 ml). The organic layer was collected, dried over $MgSO_4$, filtered and the solvent was removed under reduced pressure. The product was isolated after purification by column chromatography as a white powder in 55% - 59% yield.

2.3.3.1. ((2,6-dibromonaphthalene-1,5-diyl)bis(ethyne-2,1-diyl))bis(trimethylsilane) (12a)

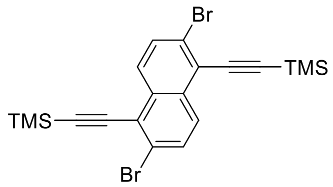

Starting from **11a** (874 mg, 1.5 mmol, 1.0 equiv.) after purification by column chromatography (SiO_2, pure CH), the product was obtained in 59% yield (395 mg, 0.9 mmol).

^{1}H NMR (500 MHz, 303 K, $CDCl_3$): δ [ppm] = 8.20 (d, $^3J_{HH}$ = 8.77 Hz, 2H, $H_{Naphtho}$); 7.58 (d, $^3J_{HH}$ = 8.73 Hz, 2H, $H_{Naphtho}$); 0.32 (s, 9H, H_{TMS}).

^{13}C NMR (125 MHz, 303 K, $CDCl_3$): δ [ppm] = 133.32 (1C); 131.78 (1C); 128.29 (1C); 126.29 (1C); 123.25 (1C); 106.84 (1C); 101.02 (1C); 0.32 (3C).

HRMS (EI) *m/z*: calc. [$C_{20}H_{22}Br_2Si_2$] = 477.9606, found: 477.9607.

IR: ῦ = 2959 (m), 2898 (w), 2161 (m), 1558 (m), 1480 (w), 1386 (m), 1297 (m), 1249 (s), 1176 (m), 1126 (m), 941 (s), 844 (vs), 806 (s), 957 (s), 689 (m).

2.3.3.2. ((1,5-dibromonaphthalene-2,6-diyl)bis(ethyne-2,1-diyl))bis(trimethylsilane) (12b)

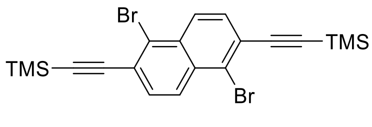

Starting from **11b** (874 mg, 1.5 mmol, 1.0 equiv.) after purification by column chromatography (SiO_2, pure CH), the product was obtained in 55% yield (395 mg, 0.8 mmol).

^{1}H NMR (500 MHz, 303 K, $CDCl_3$): δ [ppm] = 8.21 (d, $^3J_{HH}$ = 8.77 Hz, 2H, $H_{Naphtho}$); 7.58 (d, $^3J_{HH}$ = 8.73 Hz, 2H, $H_{Naphtho}$); 0.32 (s, 9H, H_{TMS}).

^{13}C NMR (125 MHz, 303 K, $CDCl_3$): δ [ppm] = 132.47 (1C); 130.99 (1C); 127.49 (1C); 126.74 (1C); 124.96 (1C); 103.84 (1C); 102.48 (1C); -0.03 (3C).

HRMS (EI) *m/z*: calc. [$C_{20}H_{22}Br_2Si_2$] = 477.9606, found: 477.9607.

IR: ũ = 3091 (vw), 2956 (m), 2897 (w), 2158 (m), 1578 (w), 1468 (m), 1345 (m), 1306 (m), 1250 (s), 1143 (m), 969 (m), 838 (vs), 817 (vs), 759 (s), 698 (s), 621 (m).

2.4. Preparing imidazole derivatives

2.4.1. Synthesis of 4,5-bis(4-methoxyphenyl)-1*H*-imidazole (SI3)

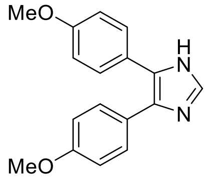

4,4′-Dimethoxybenzoin (3.26 g, 12 mmol, 1.0 equiv.) and formamide (15.0 equiv.) were added to a flask and heated to 150 ºC for 6 h. The reaction was cooled and poured into 5% HCl aqueous solution and then extracted with DCM (three times, each with 30 ml). After drying over $MgSO_4$, the solvent was removed under reduce pressure. The residue was mixed with NH_4OAc (6.0 equiv.) in EtOH (1.7 mL/mmol) and refluxed for another 6 h. After the mixture was cooled to ambient temperature, the solvent was removed and the residue was dissolved in DCM and washed with water. The organic layer was collected, dried over $MgSO_4$, filtered and the solvent was removed under reduced pressure. The product was isolated after purification by column chromatography (SiO_2, CH:EE:EtOH = 3:2:0.8). The product was obtained as white solid in 73% yield (2.46 g, 8.76 mmol).

^{1}H NMR (500 MHz, 303 K, DMSO): δ [ppm] = 12.26 (s, 1H, H_{NH}); 7.68 (s, 1H, H_{NCHN}); 7.41 (d, $^3J_{HH}$ = 8.26 Hz, 2H, H_{Ph}); 7.32 (d, $^3J_{HH}$ = 8.26 Hz, 2H, H_{Ph}); 6.96 (d, $^3J_{HH}$ = 8.12 Hz, 2H, H_{Ph}); 6.85 (d, $^3J_{HH}$ = 8.15 Hz, 2H, H_{Ph}); 3.77 (s, 3H, H_{OMe}); 3.74 (s, 3H, H_{OMe}).

^{13}C NMR (125 MHz, 303 K, DMSO): δ [ppm] = 158.51 (1C); 157.75 (1C); 135.21 (1C); 134.84 (1C); 129.12 (2C); 128.15 (1C); 128.08 (2C); 125.23 (1C); 123.82 (1C); 114.11 (2C); 113.59 (2C); 55.11 (1C); 54.98 (1C).

MS (ESI) *m/z*: calc. $[C_{17}H_{17}N_2O_2]^+$ = 281.1290, found: 281.1300; calc. $[C_{17}H_{16}N_2O_2+Na]^+$ = 303.1109, found: 303.1117;

IR: ũ = 3107 (v), 3005 (vw), 2935 (vw, 2835 (v), 1750 (vw), 1613 (m), 1588 (w), 1524 (m), 1506 (s), 1449 (m), 1414 (w), 1373 (vw), 1292 (m), 1278 (m), 1248 (vs), 1170 (vs), 1131 (m), 1107 (m), 1031 (s), 964 (w), 954 (m), 931 (vs), 799 (s), 747 (m), 659 (m), 634 (m), 624 (m), 609 (w).

2.4.2. Synthesis of 4,5-bis(4-methoxyphenyl)-1-((2-(trimethylsilyl)ethoxy)methyl)-1H-imidazole (SI4)

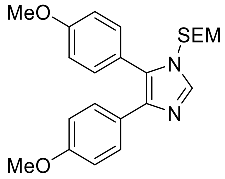

Imidazole **SI3** (1.96 g, 7.0 mmol, 1.0 equiv.) and NaH (60 wt% suspended in mineral oil, 1.5 equiv.) were added to an oven-dried Schlenk-Flask. The flask was equipped with a rubber septum, evacuated, backfilled with argon and placed in an ice bath. Dry THF (5 ml/mmol) was added slowly in the flask via a syringe. The ice bath was removed and the reaction was stirred at ambient temperature for 30 min. SEMCl (1.1 equiv.) was injected into the reaction mixture over 10 min. The reaction was stirred overnight at ambient temperature and was poured into water. The aqueous layer was extracted with DCM (three times, each with 30 ml) and the combined organic layer dried over $MgSO_4$, filtered and the solvent was removed under reduced pressure. The product was isolated after purification by column chromatography (SiO_2, CH:EE = 1:1). The product was obtained as a white solid in 93% yield (2.67 g, 6.5 mmol).

^{1}H NMR (500 MHz, 303 K, $CDCl_3$): δ [ppm] = 7.69 (s, 1H, H_{NCHN}); 7.45 (d, $^3J_{HH}$ = 8.82 Hz, 2H, H_{Ph}); 7.32 (d, $^3J_{HH}$ = 8.82 Hz, 2H, H_{Ph}); 6.96 (d, $^3J_{HH}$ = 8.77 Hz, 2H, H_{Ph}); 6.77 (d, $^3J_{HH}$ = 8.86 Hz, 2H, H_{Ph}); 5.08 (s, 2H, H_{NCH2O}); 3.86 (s, 3H, H_{OMe}); 3.77 (s, 3H, H_{OMe}); 3.46 (t, $^3J_{HH}$ = 8.25 Hz, 2H, $H_{OCH2CH2TMS}$); 0.88 (t, $^3J_{HH}$ = 8.30 Hz, 2H, $H_{OCH2CH2TMS}$); -0.02 (s, 9H, H_{TMS}).

^{13}C NMR (125 MHz, 303 K, CDCl$_3$): δ [ppm] = 159.92 (1C); 158.45 (1C); 138.47 (1C); 137.76 (1C); 132.47 (2C); 128.07 (2C); 127.53(1C); 127.49 (1C); 122.46 (1C); 114.45 (2C); 113.74 (2C); 73.91 (1C); 66.27 (1C); 55.40 (1C); 55.30 (1C); 17.93 (1C); -1.29 (3C).

MS (ESI) *m/z*: calc. $[C_{23}H_{31s}N_2O_3Si]^+$ = 411.2104, found: 411.2128; calc. $[C_{23}H_{30}N_2O_3Si+Na]^+$ = 433.1923, found: 433.1924

IR: ῦ = 5952 (w), 2837 (vw), 1709 (w), 1669 (w), 1606 (m), 1577 (w), 1519 (m), 1497 (m), 1463 (m), 1442 (w), 1442 (w), 1293 (m), 1246 (vs), 1173 (s), 1088 (s), 1030 (s), 946 (w), 920 (w), 831 (vs), 769 (s), 694 (s), 657 (w), 643 (w), 616 (vw).

2.4.3. 2-iodo-4,5-bis(4-methoxyphenyl)-1-((2-(trimethylsilyl)ethoxy)methyl)-1*H*-imidazole (7)

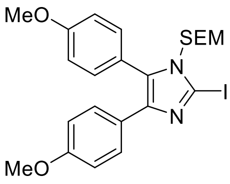

SEM-group protected imidazole **SI4** (1.82 g, 6.5 mmol, 1.0 equiv.) was added to an oven-dried Schlenk-flask. The flask was evacuated and backfilled with argon. Dry THF (10 ml/mmol) was added into the flask via a syringe and the mixture was stirred until a clear solution was obtained. The flask was cooled to -78 °C in an acetone bath. *n*-BuLi was given to the reaction mixture as a solution in hexane (2.5 M, 1.2 equiv.) over 0.5 hour and the mixture was stirred for another half hour at -78 °C. Iodine (1.24 g, 9.8 mmol, 1.5 equiv.) was dissolved in dry THF (3.6 ml/mmol), and the solution was slowly added to the mixture via syringe. Then the reaction was stirred overnight at ambient temperature before it was poured into saturated $NaHSO_3$ solution and extracted with DCM (three times, each with 30 ml). The combined organic layer was dried over $MgSO_4$, filtered and the solvent was removed under reduced pressure. The product was isolated after purification by column chromatography (SiO_2, CH:EE = 5.5:1). The product was obtained as a white solid in 76% yield (4.9 mmol, 2.64 g).

^{1}H NMR (500 MHz, 303 K, CDCl$_3$): δ [ppm] = 7.39 (d, $^3J_{HH}$ = 8.93 Hz, 2H, H_{Ph}); 7.29 (d, $^3J_{HH}$ = 8.76 Hz, 2H, H_{Ph}); 6.96 (d, $^3J_{HH}$ = 8.74 Hz, 2H, H_{Ph}); 6.75 (d, $^3J_{HH}$ = 8.95 Hz, 2H, H_{Ph}); 5.06 (s, 2H, H_{NCH2O}); 3.86 (s, 3H, H_{OMe}); 3.76 (s, 3H, H_{OMe}); 3.49 (t, $^3J_{HH}$ = 8.66 Hz, 2H, $H_{OCH2CH2TMS}$); 0.88 (t, $^3J_{HH}$ = 8.31 Hz, 2H, $H_{OCH2CH2TMS}$); -0.01 (s, 9H, H_{TMS}).

^{13}C NMR (125 MHz, 303 K, $CDCl_3$): δ [ppm] = 160.23 (1C); 158.68 (1C); 141.91 (1C); 132.64 (2C); 131.72 (1C); 128.07 (2C); 126.61 (1C); 122.39 (1C); 114.49 (2C); 113.69 (2C); 90.78 (1C); 75.32 (1C); 66.56 (1C); 55.43 (1C); 55.30 (1C); 18.11 (1C); -1.23 (3C).

MS (ESI) *m/z*: calc. $[C_{23}H_{30}N_2O_3SiI]^+$ = 537.1070, found: 537.1051; calc. $[C_{23}H_{30}N_2O_3SiI+Na]^+$ = 537.1070, found: 537.1051

IR: ũ = 3001 (vw), 2924 (vw), 2829 (vw), 1613 (m), 1581 (m), 1519 (s), 1495 (s), 1455 (m), 1392 (m), 1320 (m), 1293 (m), 1245 (vs), 1183 (s), 1171 (s), 1121 (w), 1078 (s), 1035 (s), 956 (m), 942 (m), 920 (m), 863 (m), 833 (vs), 767 (s), 728 (m), 707 (m), 667 (w), 647 (w), 634 (m).

2.5. Bis(alkyne)-imidazol precursor by Sonogashira cross-coupling

The bis(alkynes) **6**, **12a** or **12b** (1.0 equiv.) and Iodo-Imidazol **7** (2.0 equiv.) were added to a vial. The vial was sealed, degassed and backfilled with argon. In the Glovebox, $Pd(PPh_3)_4$ (0.05 equiv.), CuI (0.1 equiv.) and KF (2.2 to 2.5 equiv.) were added to the vial. Afterwards degassed THF (10 ml/mmol) and TEA (5ml/mmol) were injected via syringe. The reaction was stirred at 50 to 80 °C overnight. Then the reaction mixture was poured into saturated NH_4Cl solution and extracted with DCM three times (each with 30 ml). The organic layer was gathered, dried over $MgSO_4$, filtered and the solvent was removed under reduced pressure. The product was purified by column chromatography and obtained as yellow to dark orange solid in 58 to 92 % yield**.**

2.5.1. 2,2'-((2,5-dibromo-1,4-phenylene)bis(ethyne-2,1-diyl))bis(4,5-bis(4-methoxyphenyl)-1-((2-(trimethylsilyl)ethoxy)methyl)-1H-imidazole) (8)

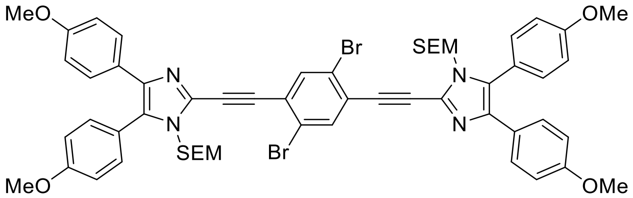

Starting from **6** (86 mg, 0.20 mmol, 1.0 equiv.) and **7** (214.6 mg, 0.40 mmol, 2.0 equiv.) after purification by column chromatography (SiO_2, CH:EE = 3.5:1), the product was obtained as a yellow solid in 80% yield (177 mg, 0.16 mmol).

^{1}H NMR (500 MHz, 303 K, $CDCl_3$): δ [ppm] = 7.88 (s, 2H, H_{BrCCH}); 7,47 (d, $^3J_{HH}$ = 8.84 Hz, 4H, H_{PhOMe}); 7.38 (d, $^3J_{HH}$ = 8.74 Hz, 4H, H_{PhOMe}); 6.96 (d, $^3J_{HH}$ = 8.82 Hz, 4H, H_{PhOMe}); 6.77 (d, $^3J_{HH}$ = 8.88 Hz, 4H,

H_{PhOMe}); 5.37 (s, 4H, H_{NCH2O}); 3.85 (s, 6H, H_{OMe}); 3.76 (s, 6H, H_{OMe}); 3.68 (t, $^3J_{HH}$ = 8.33 Hz, 4H, $H_{OCH2CH2TMS}$); 0.92 (t, $^3J_{HH}$ = 8.3 Hz, 4H, $H_{OCH2CH2TMS}$); -0.05 (s, 18H, H_{TMS}).

^{13}C NMR (125 MHz, 303 K, $CDCl_3$): δ [ppm] = 160.16 (2C); 158.81 (2C); 140.03 (2C); 136.47 (2C); 132.27 (4C); 130.73 (2C); 130.23 (2C); 128.45 (4C); 126.50 (2C); 125.91 (2C); 123.19 (2C); 121.70 (2C); 114.43 (4C); 113.68 (4C); 90.23 (2C); 86.98 (2C); 73.52 (2C); 66.78 (2C); 55.33 (2C); 55.21 (2C); 18.14 (2C); -1.35 (6C).

MS (ESI) *m/z*: calc. $[C_{56}H_{61}N_4O_6Si_2Br_2]^+$ = 1099.2496, found: 1101.2485; calc. $[C_{56}H_{62}N_4O_6Si_2Br_2]^{2+}$ = 551.1272, found: 551.1279

IR: ũ = 2990 (vw), 2954 (w), 2893 (w), 2831 (vw), 2218 (m), 1612 (m), 1577 (w), 1519 (s), 1488 (m), 1461 (m), 1438 (s), 1413 (m), 1319 (m), 1304 (m), 1291 (m), 1247 (vs), 1174 (s), 1089 (s), 1063 (m), 1038 (s), 966 (m), 920 (m), 888 (w), 857 (s), 835 (vs), 794 (m), 757 (m), 739 (m), 698 (m), 657 (w).

2.5.2. 2,2'-((2,6-dibromonaphthalene-1,5-diyl)bis(ethyne-2,1-diyl))bis(4,5-bis(4-methoxyphenyl) -1-((2-(trimethylsilyl)ethoxy)methyl)-1H-imidazole) (13a)

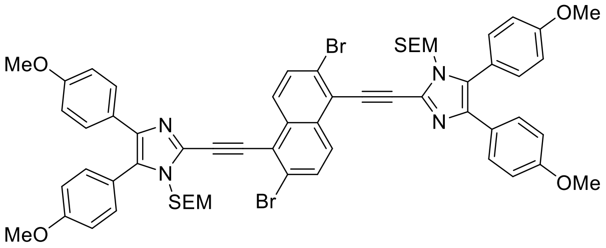

Starting from **12a** (72 mg, 0.15 mmol, 1.0 equiv.) and **7** (161 mg, 0.30 mmol, 2.0 equiv.) after purification by column chromatography (SiO_2, CH:DCM:EE = 3:1:0.7), the product was obtained as a yellow solid in 58% yield (100 mg, 0.09 mmol).

^{1}H NMR (500 MHz, 303 K, $CDCl_3$): δ [ppm] = 8.45 (d, $^3J_{HH}$ = 8.90 Hz, 2H, $H_{Naphtho}$); 7.80 (d, $^3J_{HH}$ = 8.92 Hz, 2H, $H_{Naphtho}$); 7,49 (d, $^3J_{HH}$ = 8.79 Hz, 4H, H_{PhOMe}); 7.41 (d, $^3J_{HH}$ = 8.77 Hz, 4H, H_{PhOMe}); 6.98 (d, $^3J_{HH}$ = 8.78 Hz, 4H, H_{PhOMe}); 6.81 (d, $^3J_{HH}$ = 8.85 Hz, 4H, H_{PhOMe}); 5.47 (s, 4H, H_{NCH2O}); 3.87 (s, 6H, H_{OMe}); 3.79 (s, 6H, H_{OMe}); 3.70 (t, $^3J_{HH}$ = 8.35 Hz, 4H, $H_{OCH2CH2TMS}$); 0.92 (t, $^3J_{HH}$ = 8.33 Hz, 4H, $H_{OCH2CH2TMS}$); -0.01 (s, 18H, H_{TMS}).

^{13}C NMR (125 MHz, 303 K, $CDCl_3$): δ [ppm] = 160.21 (2C); 158.89 (2C); 140.04 (2C); 133.11 (2C); 132.48 (4C); 131.77 (2C); 131.25 (2C); 130.19 (2C); 128.91 (4C); 128.66 (2C); 126.72 (2C); 125.82 (2C); 122.13 (2C); 121.91 (2C); 114.50 (4C); 113.82 (4C); 89.84 (2C); 89.50 (2C); 73.61 (2C); 66.83 (2C); 55.42 (2C); 55.32 (2C); 18.21 (2C); -1.35 (6C).

MS (ESI) *m/z*: calc. $[C_{60}H_6N_4O_6Si_2Br_2]^+$ = 1151.2627, found: 1151.2616

IR: ũ = 2951 (w), 2895 (w), 2835 (vw), 2361 (vw), 2333 (vw), 2205 (vw), 1614 (m), 1576 (w), 1560 (w), 1521 (s), 1489 (s), 1465 (m), 1442 (m), 1410 (w), 1362 (w), 1322 (w), 1294 (m), 1247 (vs), 1175 (s), 1085 (s), 1031 (s), 968 (w), 938 (w9, 915 (w), 834 (vs), 814 (vs), 750 (m), 696 (m), 670 (m), 630 (w), 602 (m), 538 (m), 403 (w), 384 (w).

2.5.3. 2,2'-((1,5-dibromonaphthalene-2,6-diyl)bis(ethyne-2,1-diyl))bis(4,5-bis(4-methoxyphenyl) -1-((2-(trimethylsilyl)ethoxy)methyl)-1H-imidazole) (13b)

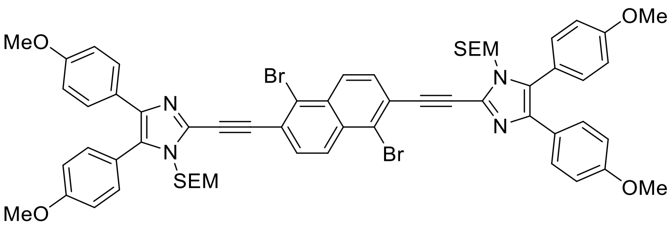

Starting from **12b** (96 mg, 0.20 mmol, 1.0 equiv.) and **7** (215 mg, 0.40 mmol, 2.0 equiv.) after purification by column chromatography (SiO_2, CH:DCM:EE = 3:0.8:0.8), the product was obtained as a yellow solid in 65% yield (150.1 mg, 0.13 mmol).

^{1}H NMR (500 MHz, 303 K, $CDCl_3$): δ [ppm] = 8.29 (d, $^3J_{HH}$ = 8.74 Hz, 2H, $H_{Naphtho}$); 7.79 (d, $^3J_{HH}$ = 8.80 Hz, 2H, $H_{Naphtho}$); 7,49 (d, $^3J_{HH}$ = 8.85 Hz, 4H, H_{PhOMe}); 7.39 (d, $^3J_{HH}$ = 8.73 Hz, 4H, H_{PhOMe}); 6.97 (d, $^3J_{HH}$ = 8.83 Hz, 4H, H_{PhOMe}); 6.79 (d, $^3J_{HH}$ = 8.90 Hz, 4H, H_{PhOMe}); 5.44 (s, 4H, H_{NCH2O}); 3.86 (s, 6H, H_{OMe}); 3.77 (s, 6H, H_{OMe}); 3.72 (t, $^3J_{HH}$ = 8.31 Hz, 4H, $H_{OCH2CH2TMS}$); 0.93 (t, $^3J_{HH}$ = 8.33 Hz, 4H, $H_{OCH2CH2TMS}$); -0.07 (s, 18H, H_{TMS}).

^{13}C NMR (125 MHz, 303 K, $CDCl_3$): δ [ppm] = 160.15 (2C); 158.82 (2C); 139.91 (2C); 132.49 (2C); 132.42 (4C); 131.17 (2C); 130.95 (2C); 130.10 (2C); 128.50 (4C); 127.65 (2C); 126.59 (2C); 125.95 (2C); 124.26 (2C); 121.84 (2C); 114.44 (4C); 113.71 (4C); 92.46 (2C); 86.20 (2C); 73.60 (2C); 66.80 (2C); 55.36 (2C); 55.25 (2C); 18.19 (2C); -1.33 (6C).

MS (ESI) *m/z*: calc. $[C_{60}H_{63}N_4O_6Si_2Br_2]^+$ = 1151.2627, found: 1151.2640, calc; $[C_{60}H_{64}N_4O_6Si_2Br_2]^{2+}$ = 576.1350, found: 576.1350.

IR: ũ = 2955 (w), 2839 (w), 2360 (w), 2206 (w), 1721 (vw), 1679 (vw), 1611 (m), 1578 (w), 1520 (m), 1500 (m), 1484 (m), 1461 (m), 1422 (m), 1410 (w), 1364 (w), 1322 (w), 1293 (m), 1247(vs), 1174 (s), 1079 (s), 1030 (s), 967 (vw), 940 (vw), 915 (vw), 835 (vs), 815 (s), 738 (m), 695 (m), 668 (w), 617 (vw), 603 (vw), 540 (m), 403 (w), 380 (w).

2.6. Annulation to heterocacene by Pd-catalyzed C-S-cross-coupling

The annulation precursor **8**, **13a** or **13b** from chapter 2.5 (1.0 equiv.), $Pd(dba)_2$ (0.1 equiv.), Xantphos (0.2 equiv.) or dippf (0.2 equiv.), K_3PO_4 (6.0 equiv.) and KSAc (4.0 equiv.) were weigh into a crimp sealed vial under Ar atmosphere. Degassed toluene (20 – 30 ml/mmol) and acetone (10 – 15 ml/mmol) were added to the mixture. The reaction was stirred at 120 °C overnight. After the mixture was allowed to cool to r.t., 20 ml of DCM was added and filtrated through a plug of SiO_2. The volatiles were removed and the pure product was isolated after column chromatography.

2.6.1. 2,6-bis(4,5-bis(4-methoxyphenyl)-1-((2-(trimethylsilyl)ethoxy)methyl)-1H-imidazol-2-yl)benzo[1,2-b:4,5-b']dithiophene (3)

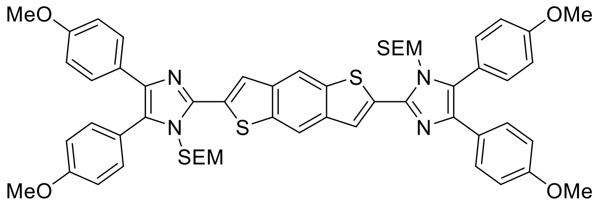

Starting from **8** (177 mg, 0.16 mmol, 1.0 equiv.) after purification by column chromatography (SiO_2, CH:EE:DCM = 2:0.5:1.8), the product was obtained as a dark orange solid in 86% yield (139 mg, 0.14 mmol).

^{1}H NMR (500 MHz, 303 K, $CDCl_3$): δ [ppm] = 8.27 (s, 2H, $H_{thiophen}$); 7.89 (s, 2H, H_{benzol}); 7,57 (d, $^3J_{HH}$ = 8.71 Hz, 4H, H_{PhOMe}); 7.36 (d, $^3J_{HH}$ = 8.21 Hz, 4H, H_{PhOMe}); 7.02 (d, $^3J_{HH}$ = 8.14 Hz, 4H, H_{PhOMe}); 6.83 (d, $^3J_{HH}$ = 8.62 Hz, 4H, H_{PhOMe}); 5.29 (s, 4H, H_{NCH2O}); 3.89 (s, 6H, H_{OMe}); 3.79 (s, 6H, H_{OMe}); 3.46 (t, $^3J_{HH}$ = 8.35 Hz, 4H, $H_{OCH2CH2TMS}$); 0.96 (t, $^3J_{HH}$ = 8.25 Hz, 4H, $H_{OCH2CH2TMS}$); -0.05 (s, 18H, H_{TMS}).

^{13}C NMR (125 MHz, 303 K, $CDCl_3$): δ [ppm] = 160.18 (2C); 158.61 (2C); 142.42 (2C); 138.83 (2C); 138.33 (2C); 137.58 (2C); 133.99 (2C); 132.82 (4C); 130.06 (2C); 128.36 (4C); 127.00 (2C); 122.56 (2C); 121.96 (2C); 117.00 (2C); 114.58 (4C); 113.69 (4C); 72.84 (2C); 66.13 (2C); 55.42 (2C); 55.28 (2C); 18.12 (2C); -1.27 (6C).

MS (ESI) *m/z*: calc. $[C_{56}H_{63}N_4O_6Si_2S_2]^+$ = 1007.3728, found: 1007.3740.

IR: ṽ = 2955 (w), 2837 (vw), 2361 (w), 2338 (w), 1669 (vw), 1611 (w), 1579 (vw), 1519 (m), 1494 (m), 1462 (w), 1440 (w), 1364 (w), 1327 (w), 1292 (w), 1249 (vs), 1174 (s), 1079 (s), 1033 (s), 972 (w), 935 (w), 914 (w), 861 (m), 834 (vs), 800 (m), 767 (m), 747 (m), 696 (m), 530 (w), 423 (vw).

2.6.2. 2,7-bis(4,5-bis(4-methoxyphenyl)-1-((2-(trimethylsilyl)ethoxy)methyl)-1H-imidazol-2-yl)naphtho[2,1-b:6,5-b']dithiophene (9a)

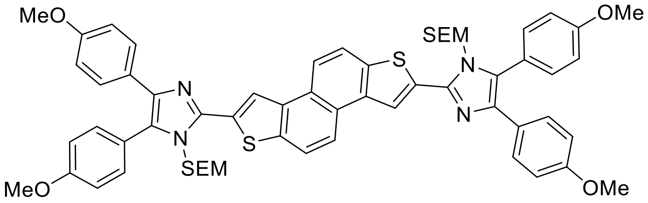

Starting from **13a** (154 mg, 0.13 mmol, 1.0 equiv.) after purification by column chromatography (SiO_2, CH:EE:DCM = 2:2:0.5), the product was obtained as a dark orange solid in 58% yield (82 mg, 0.08 mmol).

^{1}H NMR (500 MHz, 303 K, $CDCl_3$): δ [ppm] = 8.51 (s, 2H, $H_{thiophen}$); 8.27 (d, $^3J_{HH}$ = 8.82 Hz, 2H, $H_{naphtho}$); 8.02 (d, $^3J_{HH}$ = 8.69 Hz, 2H, $H_{Naphtho}$); 7,54 (d, $^3J_{HH}$ = 8.77 Hz, 4H, H_{PhOMe}); 7.38 (d, $^3J_{HH}$ = 8.65 Hz, 4H, H_{PhOMe}); 7.03 (d, $^3J_{HH}$ = 8.63 Hz, 4H, H_{PhOMe}); 6.82 (d, $^3J_{HH}$ = 8.87 Hz, 4H, H_{PhOMe}); 5.31 (s, 4H, H_{NCH2O}); 3.91 (s, 6H, H_{OMe}); 3.81 (s, 6H, H_{OMe}); 3.52 (t, $^3J_{HH}$ = 8.44 Hz, 4H, $H_{OCH2CH2TMS}$); 1.03 (t, $^3J_{HH}$ = 8.48 Hz, 4H, $H_{OCH2CH2TMS}$); 0.05 (s, 18H, H_{TMS}).

^{13}C NMR (125 MHz, 303 K, $CDCl_3$): δ [ppm] = 160.13 (2C); 158.60 (2C); 142.55 (2C); 138.15 (2C); 137.45 (2C); 137.26 (2C); 133.40 (2C); 132.78 (4C); 129.97 (2C); 128.35 (4C); 127.09 (2C); 126.79 (2C); 122.56 (2C); 121.24 (2C); 121.11 (2C); 120.77 (2C); 114.57 (4C); 113.72 (4C); 72.92 (2C); 65.98 (2C); 55.41 (2C); 55.29 (2C); 18.26 (2C); -1.22 (6C).

MS (ESI) *m/z*: calc. $[C_{60}H_{65}N_4O_6Si_2S_2]^+$ = 1057.3884, found: 1057.3887; calc. $[C_{60}H_{66}N_4O_6Si_2S_2]^+$ = 529.1976, found: 529.1984

IR: ṻ = 2951 (w), 2926 (w), 2834 (vw), 1737 (vw), 1714 (vw), 1665 (vw), 1612 (m), 1519 (s), 1494 (s), 1465 (m), 1439 (m), 1402 (m), 1364 (m), 1330 (w), 1292 (m), 1247 (vs), 1175 (s), 1077 (s), 1030 (s), 970 (w), 935 (w), 859 (s), 834 (vs), 800 (s), 747 (s), 718 (m), 695 (s), 654 (m), 594 (m), 536 (s), 459 (w), 410 (w).

2.6.3. 2,7-bis(4,5-bis(4-methoxyphenyl)-1-((2-(trimethylsilyl)ethoxy)methyl)-1H-imidazol-2-yl)naphtho[1,2-b:5,6-b']dithiophene (9b)

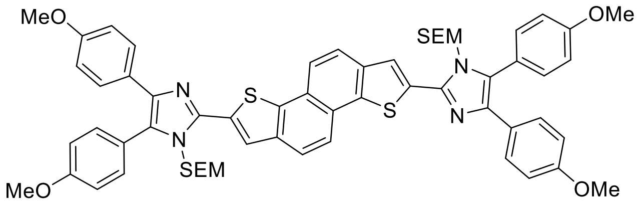

Starting from **13b** (150 mg, 0.13 mmol, 1.0 equiv.) after purification by column chromatography (SiO_2, CH:EE:DCM = 2:3:0.3) the product was obtained as a dark orange solid in 66% yield (91 mg, 0.09 mmol).

^{1}H NMR (500 MHz, 303 K, $CDCl_3$): δ [ppm] = 8.10 (d, $^3J_{HH}$ = 8.63 Hz, 2H, $H_{Naphtho}$); 8.00 (s, 2H, $H_{thiophen}$); 7.93 (d, $^3J_{HH}$ = 8.70 Hz, 2H, $H_{Naphtho}$); 7,59 (d, $^3J_{HH}$ = 8.60 Hz, 4H, H_{PhOMe}); 7.39 (d, $^3J_{HH}$ = 8.60 Hz, 4H, H_{PhOMe}); 7.04 (d, $^3J_{HH}$ = 8.58 Hz, 4H, H_{PhOMe}); 6.85 (d, $^3J_{HH}$ = 8.88 Hz, 4H, H_{PhOMe}); 5.31 (s, 4H, H_{NCH2O}); 3.90 (s, 6H, H_{OMe}); 3.82 (s, 6H, H_{OMe}); 3.47 (t, $^3J_{HH}$ = 8.28 Hz, 4H, $H_{OCH2CH2TMS}$); 0.96 (t, $^3J_{HH}$ = 8.30 Hz, 4H, $H_{OCH2CH2TMS}$); 0.05 (s, 18H, H_{TMS}).

^{13}C NMR (125 MHz, 303 K, $CDCl_3$): δ [ppm] = 160.15 (2C); 158.60 (2C); 142.45 (2C); 139.00 (2C); 138.21 (2C); 138.16 (2C); 132.83 (4C); 132.63 (2C); 129.86 (2C); 128.38 (4C); 127.04 (2C); 126.05 (2C); 124.03 (2C); 123.11 (2C); 122.61 (2C); 121.62 (2C); 114.58 (4C); 113.71 (4C); 72.83 (2C); 66.12 (2C); 55.41 (2C); 55.28 (2C); 18.11 (2C); -1.27 (6C).

MS (ESI) *m/z*: calc. $[C_{60}H_{65}N_4O_6Si_2S_2]^+$ = 1057.3884, found: 1057.3907.

IR: ῦ = 2952 (w), 2925 (w), 2899 (vw), 2833 (vw), 2362 (vw), 1612 (m), 1573 (w), 1518 (s), 1493 (s), 1459 (m), 1442 (m), 1385 (w), 1367 (w), 1323 (m), 1294 (m), 1248 (vs), 1175 (s), 1083 (s), 1033 (s), 971 (w), 921 (w), 860 (s), 832 (vs), 806 (s), 771 (s), 746 (m), 716 (m), 689 (s), 648 (w), 635 (w), 613 (w), 519 (m), 553 (m), 536 (w), 389 (vw).

2.7. Deprotection of SEM-group and oxidation of deprotected precursors with Fe^{3+} to quinoidal form

2.7.1. General deprotection procedure of SEM group

The deprotection of the SEM-group was performed in THF with excessive load of HCl (1.5 M in dioxane, 0.02 ml/mg) at 50 °C overnight. After cooling to r.t., a yellow to light orange precipitation was formed and the precipitate was isolated and rinsed with solvent (DCM:CH = 2:1) two times. Then the precipitate was centrifuged, filtered and dried in vaccum to afford a pale yellow to brown solid. Due to the very poor solubility of the products in organic solvents, NMR-data cannot be recorded but MS spectrometry confirmed the successful deprotection. The products were used in the oxidation step without further purification.

2.7.1.1. 2,6-bis(4,5-bis(4-methoxyphenyl)-1H-imidazol-2-yl)benzo[1,2-b:4,5-b']dithiophene (SI5)

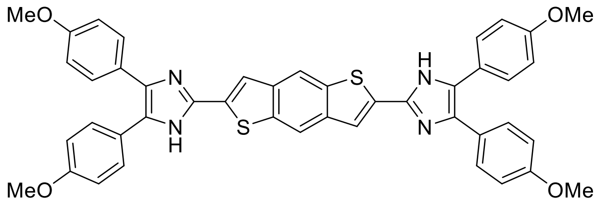

Starting from **3** (95 mg, 0.09 mmol, 1.0 equiv.), the product was obtained as a yellow solid in 84% yield (59.2 mg, 0.08 mmol).

MS (ESI) *m/z*: calc. $[C_{44}H_{35}N_4O_4S_2]^+$ = 747.2100, found: 747.2120; calc. $[C_{44}H_{36}N_4O_4S_2]^{2+}$ = 374.1083, found: 374.1092.

IR: ῦ = 3351 (b), 2562 (b), 1637 (s), 1612 (s), 1575 (m), 1518 (m), 1493 (s), 1440 (m), 1299 (m), 1252 (vs), 1175 (s), 1025 (s), 875 (s), 831 (vs), 801 (m), 573 (m), 531 (m), 516(s), 418 (m), 399 (m).

2.7.1.2. 2,7-bis(4,5-bis(4-methoxyphenyl)-1H-imidazol-2-yl)naphtho[2,1-b:6,5-b']dithiophene (SI6)

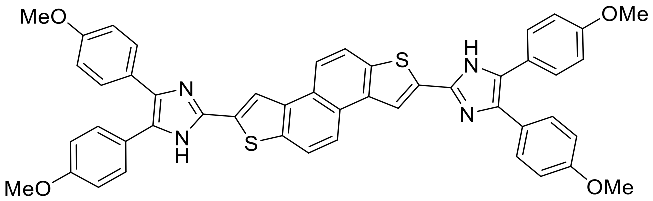

Starting from **9a** (112 mg, 0.11 mmol, 1.0 equiv.), the product was obtained as a yellow solid in 73% yield (61.7 mg, 0.08 mmol).

MS (ESI) *m/z*: calc. $[C_{48}H_{37}N_4O_4S_2]^+$ = 797.2256, found: 797.2294; calc. $[C_{48}H_{38}N_4O_4S_2]^{2+}$ = 399.1662, found: 399.1187.

IR: ῦ = 3384 (b), 3056 (vw), 2937 (w), 2836 (w), 2516 (b), 1638 (s), 1612 (s), 1575 (m), 1529 (m), 1503 (s), 1478 (s), 1464 (s), 1409 (m), 1299 (s), 1251 (vs), 1177 (s), 1113 (m), 1089 (w), 1071 (w), 1027 (s), 877 (m), 832 (vs), 803 (s), 731 (m), 709 (m), 696 (m), 646 (w), 608 (w), 575 (w), 535 (s), 519 (s), 466 (m), 410 (m), 384 (s).

2.7.1.3. 2,7-bis(4,5-bis(4-methoxyphenyl)-1H-imidazol-2-yl)naphtho[1,2-b:5,6-b']dithiophene (SI7)

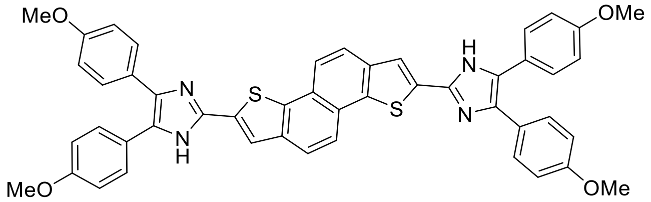

Starting from **9b** (67 mg, 0.06 mmol. 1.0 equiv.), the product was obtained as a yellow solid in 80% yield (41 mg, 0.05 mmol).

MS (ESI) *m/z*: calc. [$C_{48}H_{37}N_4O_4S_2$]$^+$ = 797.2256, found: 797.2267; calc. [$C_{48}H_{38}N_4O_4S_2$]$^{2+}$ = 399.1662, found: 399.1174.

IR: ῦ = 3394 (b), 2936 (w), 2835 (w), 2534 (b), 1637 (s), 1612 (s), 1577 (m), 155 (m), 1528 (m), 1501 (s), 1465 (s), 1440 (s), 1418 (m), 1406 (m), 1374 (w), 1299 (s), 1252 (vs), 1178 (s), 1113 (m), 1084 (m), 1072 (s), 1029 (s), 884 (w), 861 (w), 833 (s), 802 (s), 740 (w9, 730 (w), 709 (w), 698 (w), 685 (m), 644 (w), 624 (w), 609 (w), 580 (w), 533 (s), 520 (s), 4100 (w), 388 (m).

2.7.2. General procedure for oxidation of precursor to quinoids

The bis(imidazoles) **SI5**, **SI6** or**SI7** were added to dioxane (0.07 – 0.35 ml/mg). The suspension was sonicated for 10 min. After cooling to 0 °C a 1 M solution of NaOH (aq) was added. Then the mixture was flushed with nitrogen gas and a 0.04 M solution of $K_3[Fe(CN)_6]$ (aq) was added. A dark green to black-blue precipitate was formed instantly. The mixture was stirred for 0.5 – 3 h at ambient temperature and the precipitate was isolated by centrifugation and dried in vacuum.

2.7.2.1. 2,6-bis(4,5-bis(4-methoxyphenyl)-2H-imidazol-2-ylidene)-2,6-dihydrobenzo[1,2-b:4,5-b']dithiophene (14)

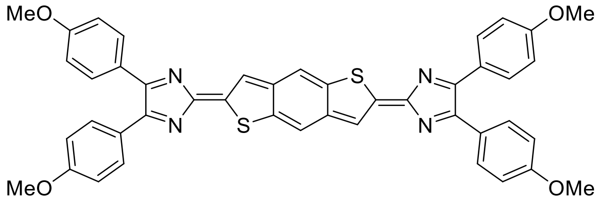

Starting from **SI5** (50 mg, 0.07 mmol, 1.0 equiv.), the product was obtained as a black solid in 83% yield (41 mg, 0.06 mmol).

MS (ESI) *m/z*: calc. $[C_{44}H_{33}N_4O_4S_2]^+$ = 745.1943, found: 745.1965; calc. $[C_{44}H_{34}N_4O_4S_2]^{2+}$ = 373.1005, found: 373.1029 .

IR: ũ = 3645 (b), 2934 (vw), 2831 (vw), 1597 (s), 1517 (m), 1497 (m), 1460 (m), 1395 (m), 1300 (m), 1244 (vs), 1169 (vs), 1110 (m), 1027 (s), 1002 (s), 890 (s), 832 (s), 721 (w), 628 (m), 603 (m), 573 (w), 528 (m), 417 (m), 380 (w).

2.7.2.2. 2,7-bis(4,5-bis(4-methoxyphenyl)-2H-imidazol-2-ylidene)-2,7-dihydronaphtho[2,1-b:6,5-b']dithiophene (15a)

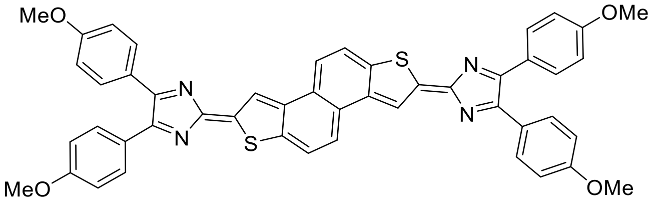

Starting from **SI6** (60 mg, 0.08 mmol, 1.0 equiv.), the product was obtained as a dark brown solid in 83% yield (52 mg, 0.06 mmol).

MS (ESI) *m/z*: calc. $[C_{48}H_{35}N_4O_4S_2]^+$ = 795.2100, found: 795.2155; calc. $[C_{48}H_{38}N_4O_4S_2]^{2+}$ = 398.1083, found: 398.1089.

IR: ũ = 2933 (vw), 2836 (vw), 1600 (s), 1540 (w), 1517 (m), 1495 (m), 1464 (m), 1439 (w), 1399 (m), 1364 (m), 1338 (w), 1303 (m), 1250 (vs), 1175 (vs), 1110 (m), 1029 (s), 970 (w), 943 (w), 878 (w), 834 (vs), 802 (m), 748 (m), 724 (m), 695 (m), 645 (m), 606 (m), 585 (m), 538 (s), 429 (w), 389 (w).

2.7.2.3. 2,7-bis(4,5-bis(4-methoxyphenyl)-2H-imidazol-2-ylidene)-2,7-dihydronaphtho[1,2-b:5,6-b']dithiophene (15b)

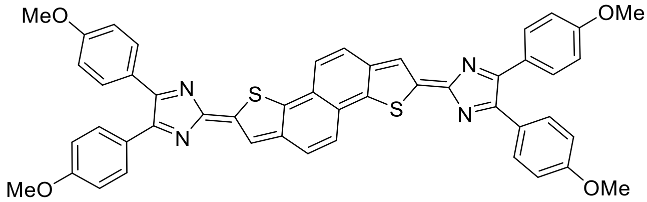

Starting from **SI7** (40 mg, 0.05 mmol, 1.0 equiv.), the product was obtained as a black solid in 82% yield (33 mg, 0.04 mmol).

MS (ESI) *m/z*: calc. $[C_{48}H_{35}N_4O_4S_2]^+$ = 795.2100, found: 795.2097; calc. $[C_{48}H_{38}N_4O_4S_2]^{2+}$ = 398.1083, found: 398.1082.

IR: ṽ = 2894 (vw), 2834 (vw), 1599 (s), 1511 (m), 1494 (m), 1462 (w), 1440 (w), 1373 (m), 1303 (m), 1249 (s), 1172 (s), 1109 (w), 1027 (s), 970 (w), 893 (w), 833 (s), 803 (m), 745 (w), 723 (w), 687 (w), 625 (w), 590 (w), 528 (m), 395 (w).

3. Characterization

3.1. NMR-spectra

SI1 in DMSO, ^{1}H NMR (303 K, 500 MHz)

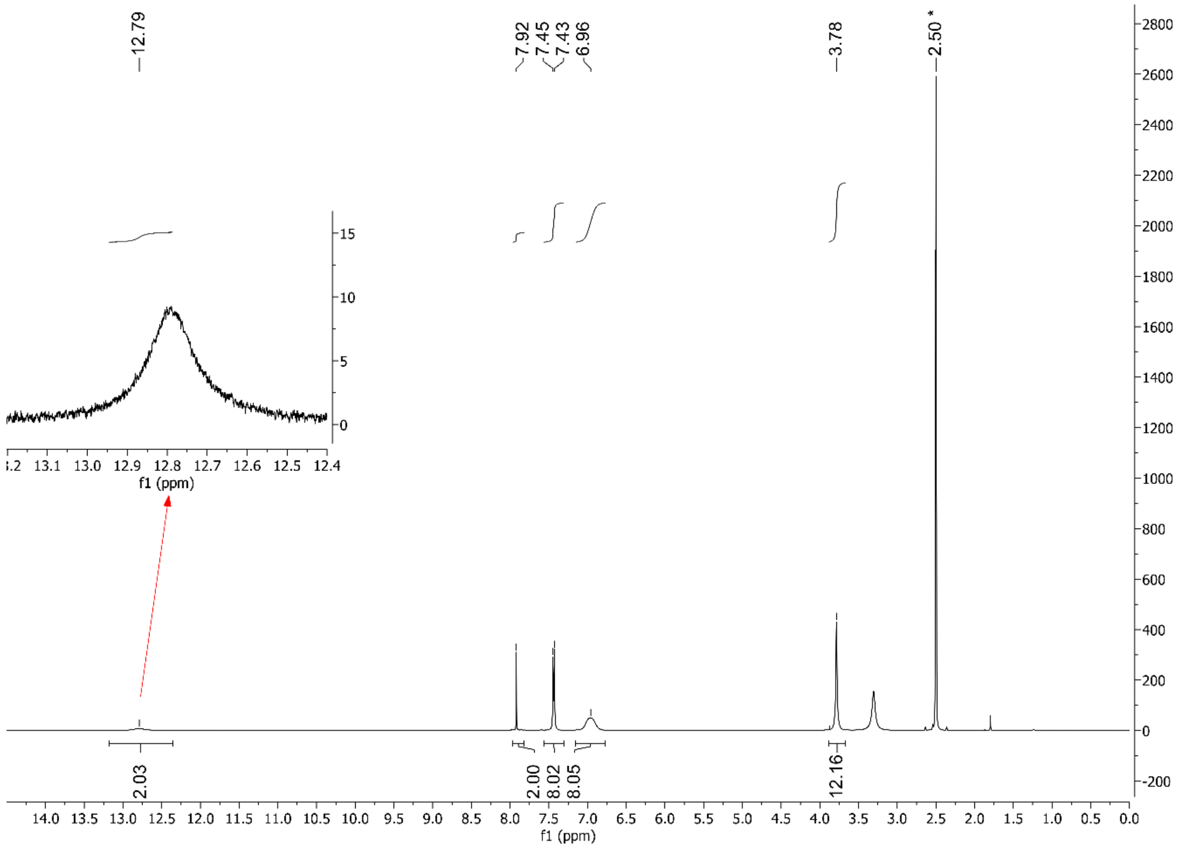

1 in $CDCl_3$, ^{1}H NMR (303 K, 300 MHz)

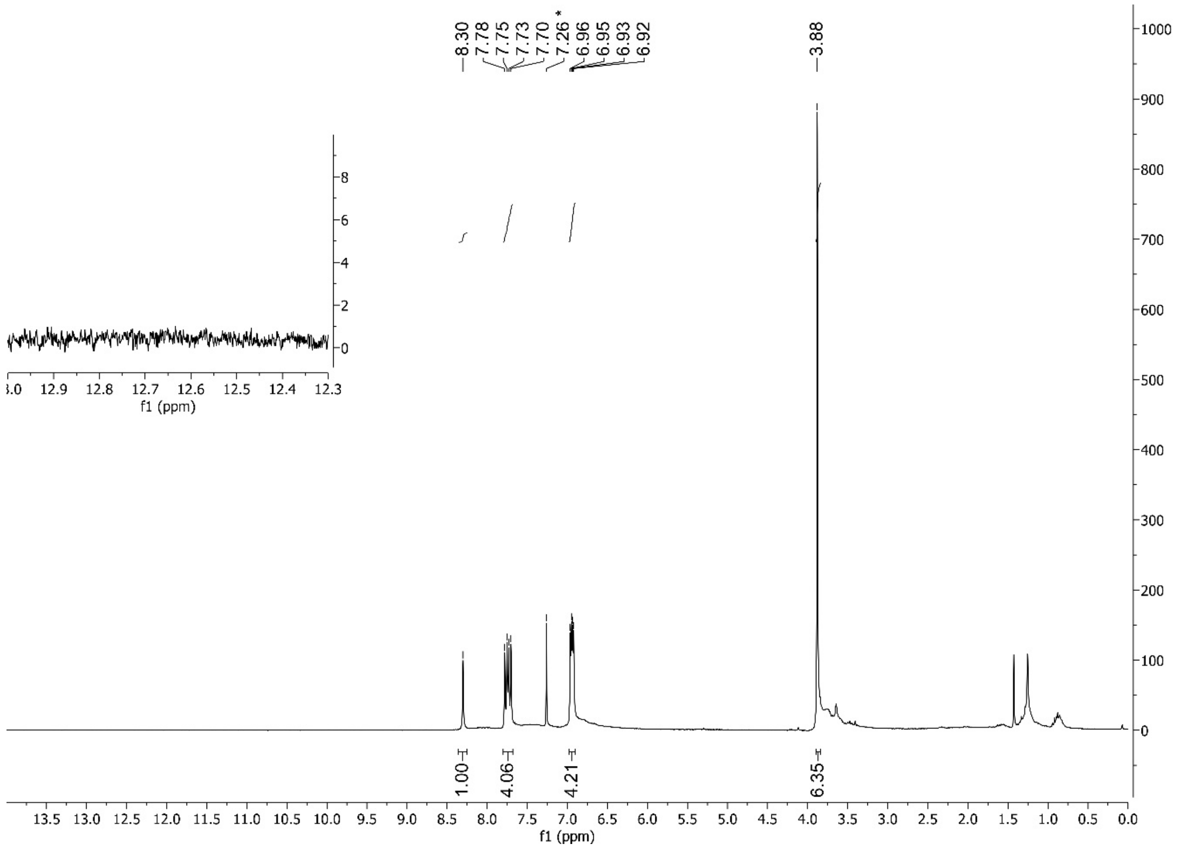

1 in $CDCl_3$, ^{13}C NMR (303 K, 125 MHz)

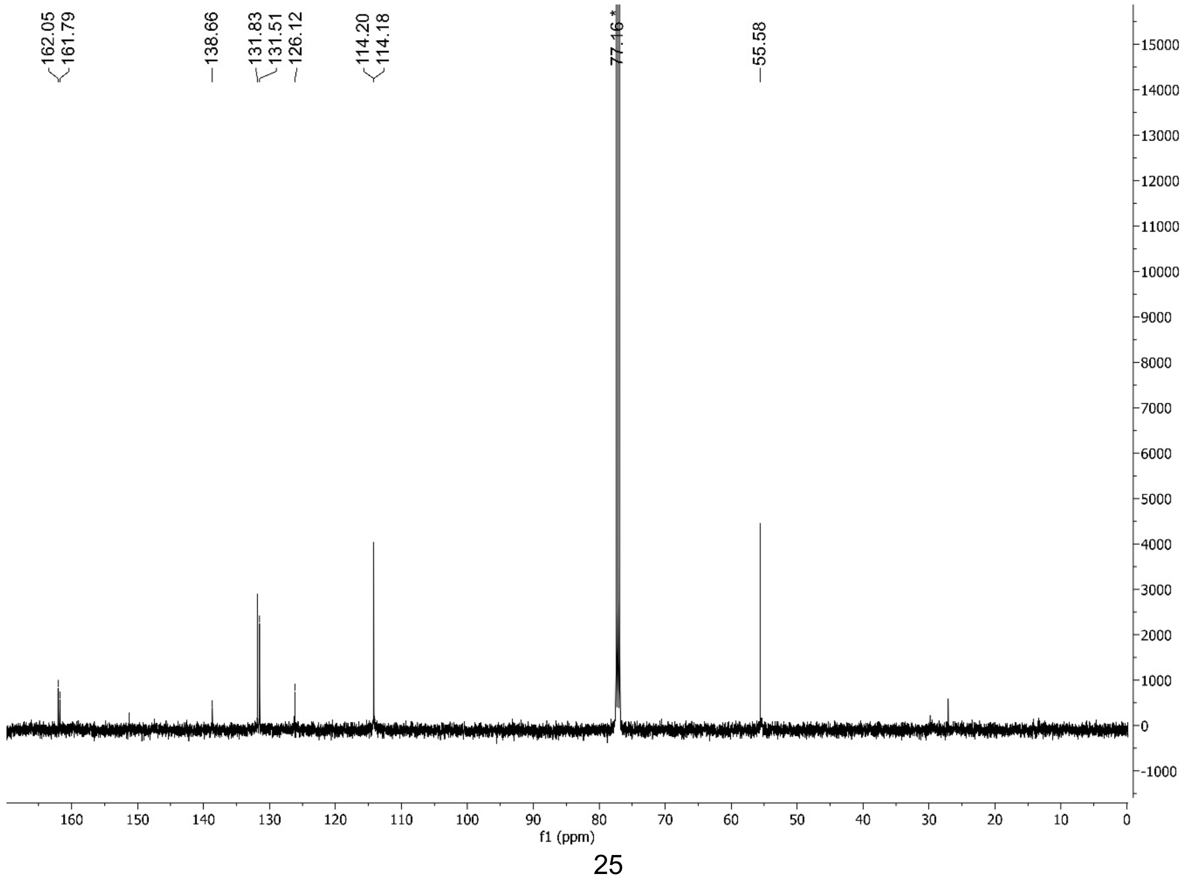

SI2 in DMSO, ^{1}H NMR (303 K, 300 MHz)

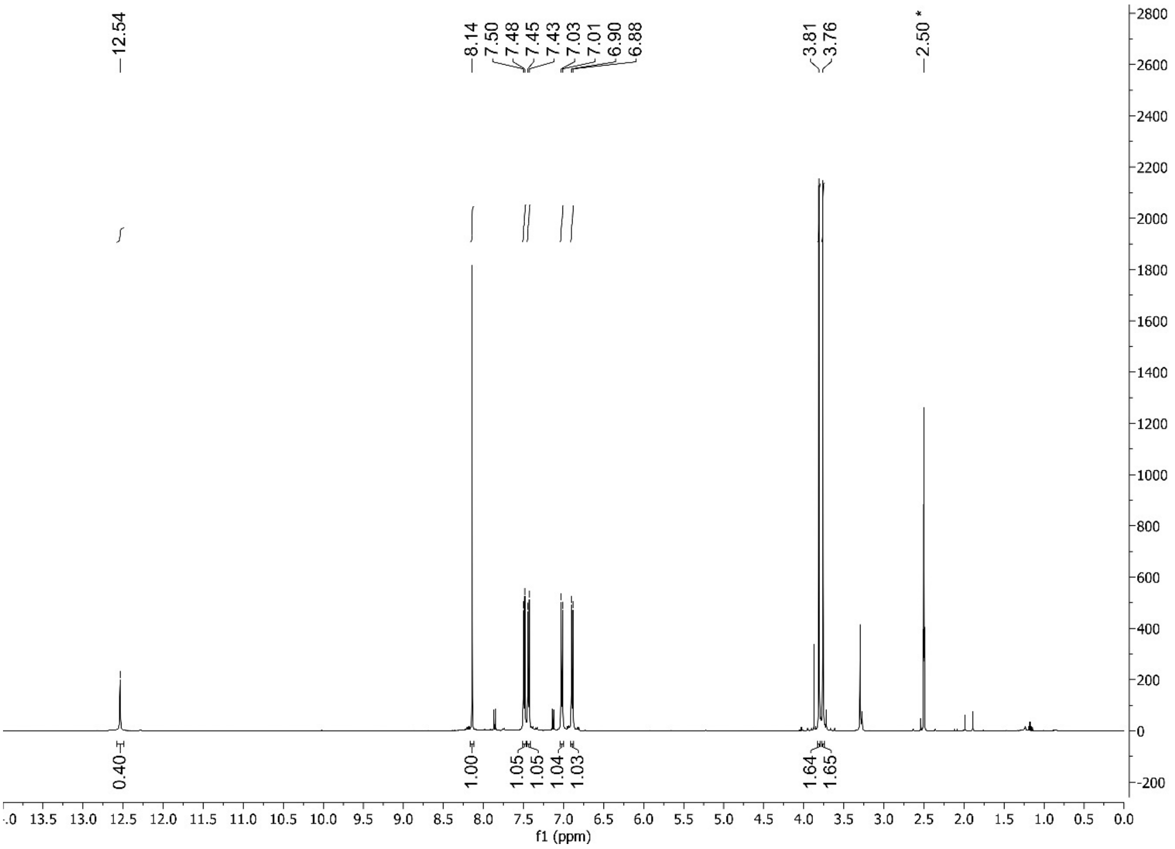

SI2 in DMSO, ^{13}C NMR (303 K, 125 MHz)

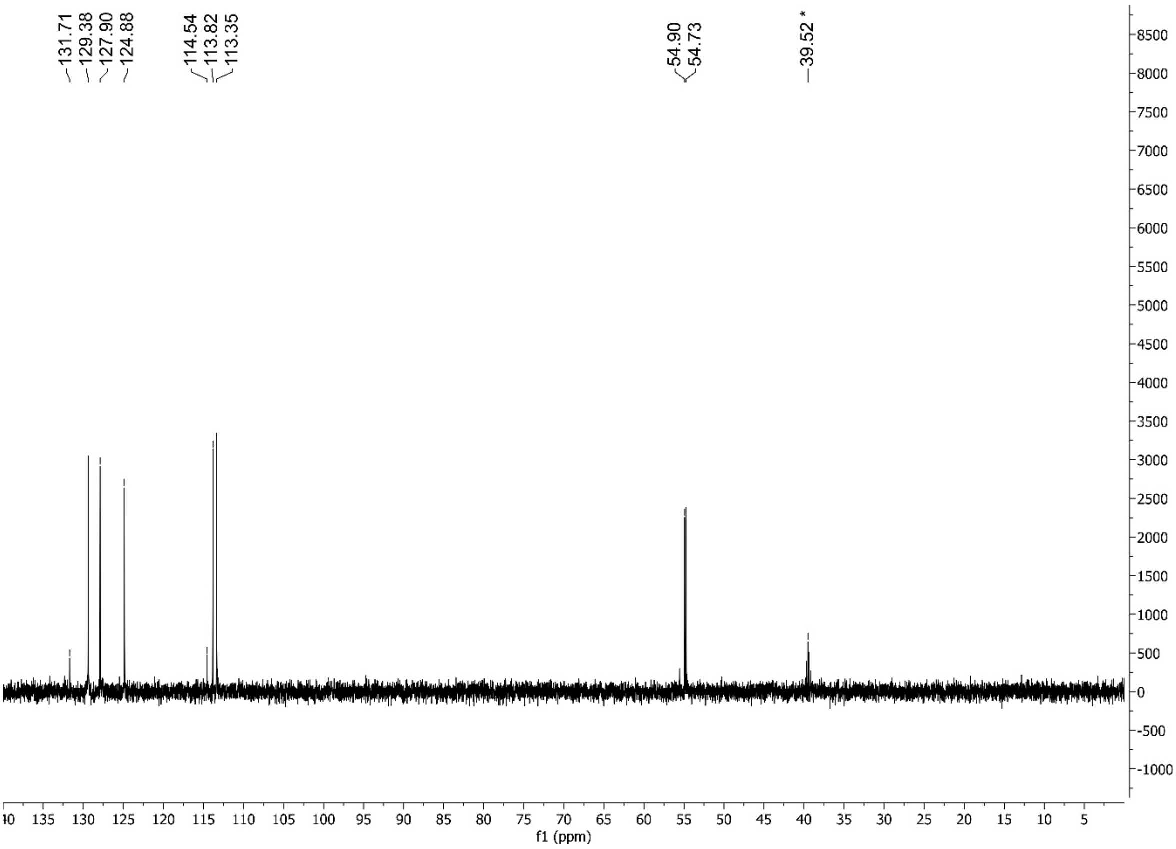

2 in $CDCl_3$, ^{1}H NMR (303 K, 700 MHz)

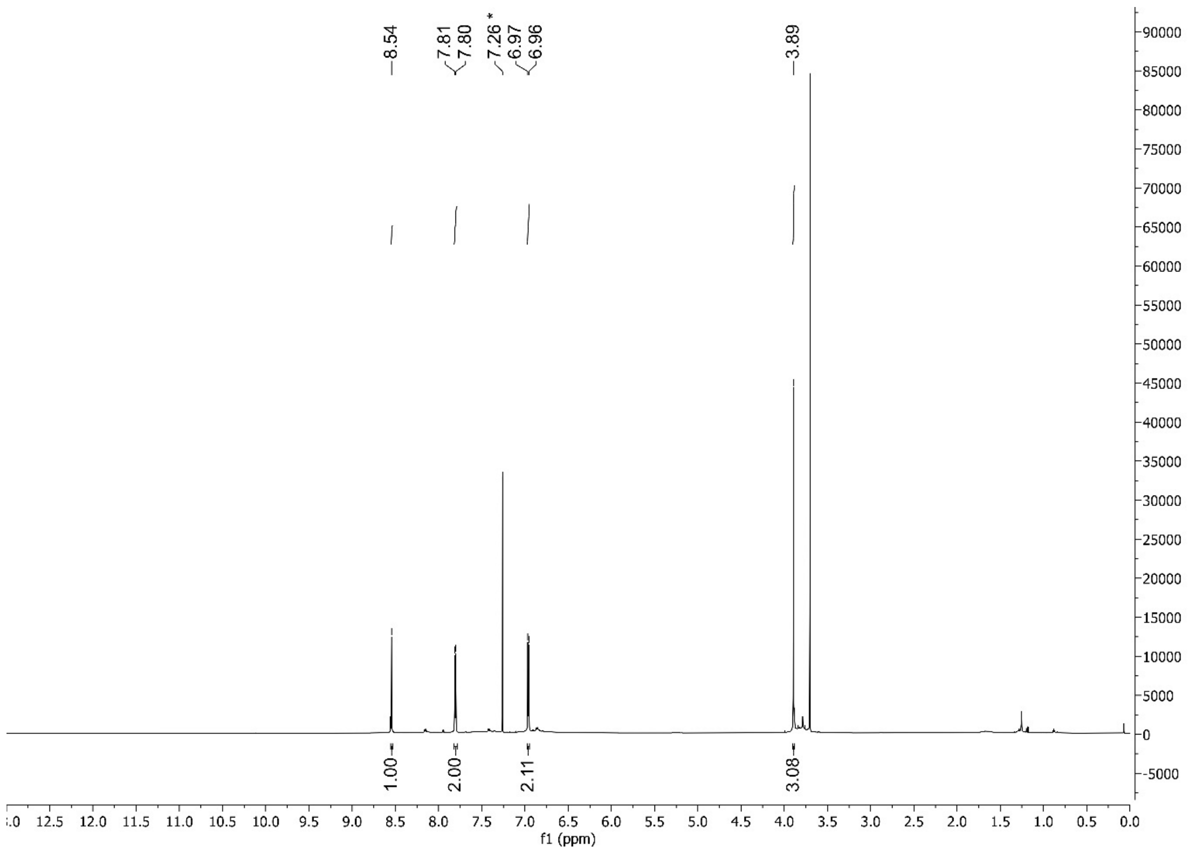

2 in $CDCl_3$, ^{13}C NMR (303 K, 176 MHz)

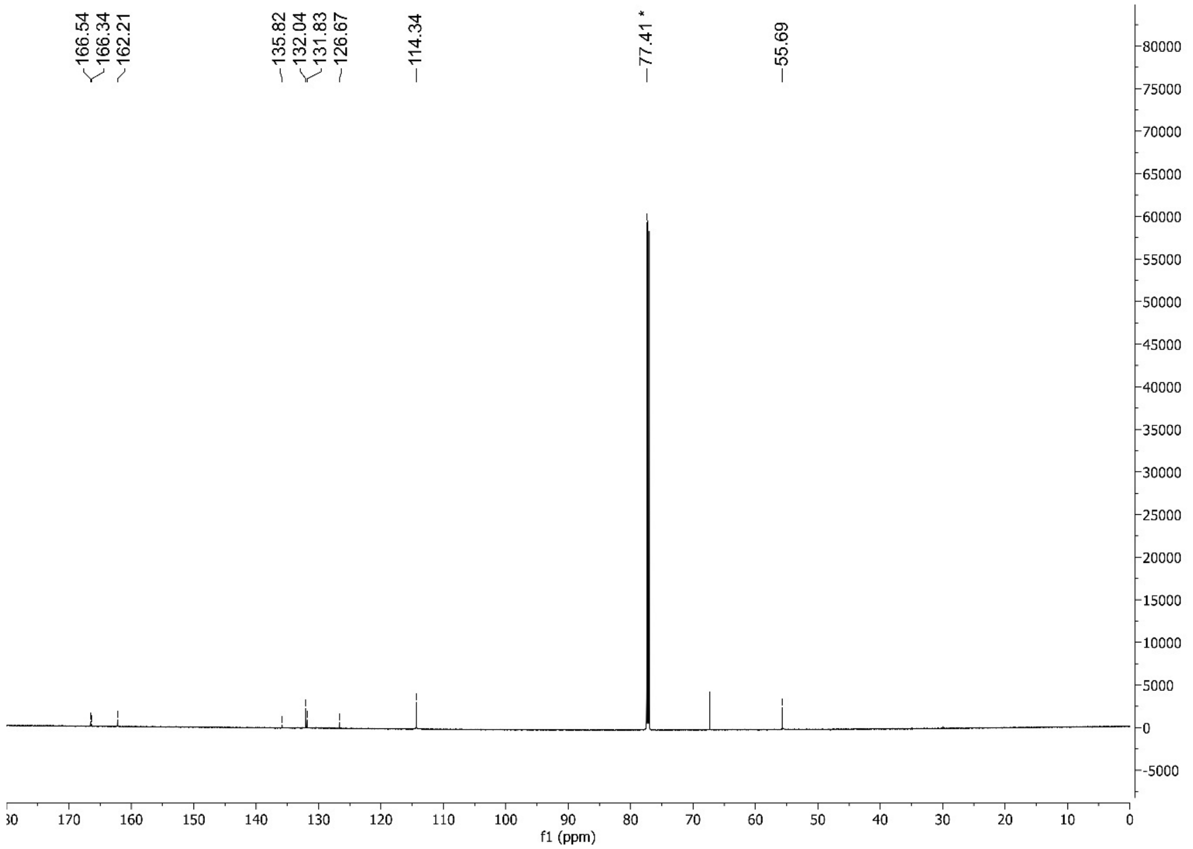

6 in $CDCl_3$, ^{1}H NMR (303 K, 500 MHz)

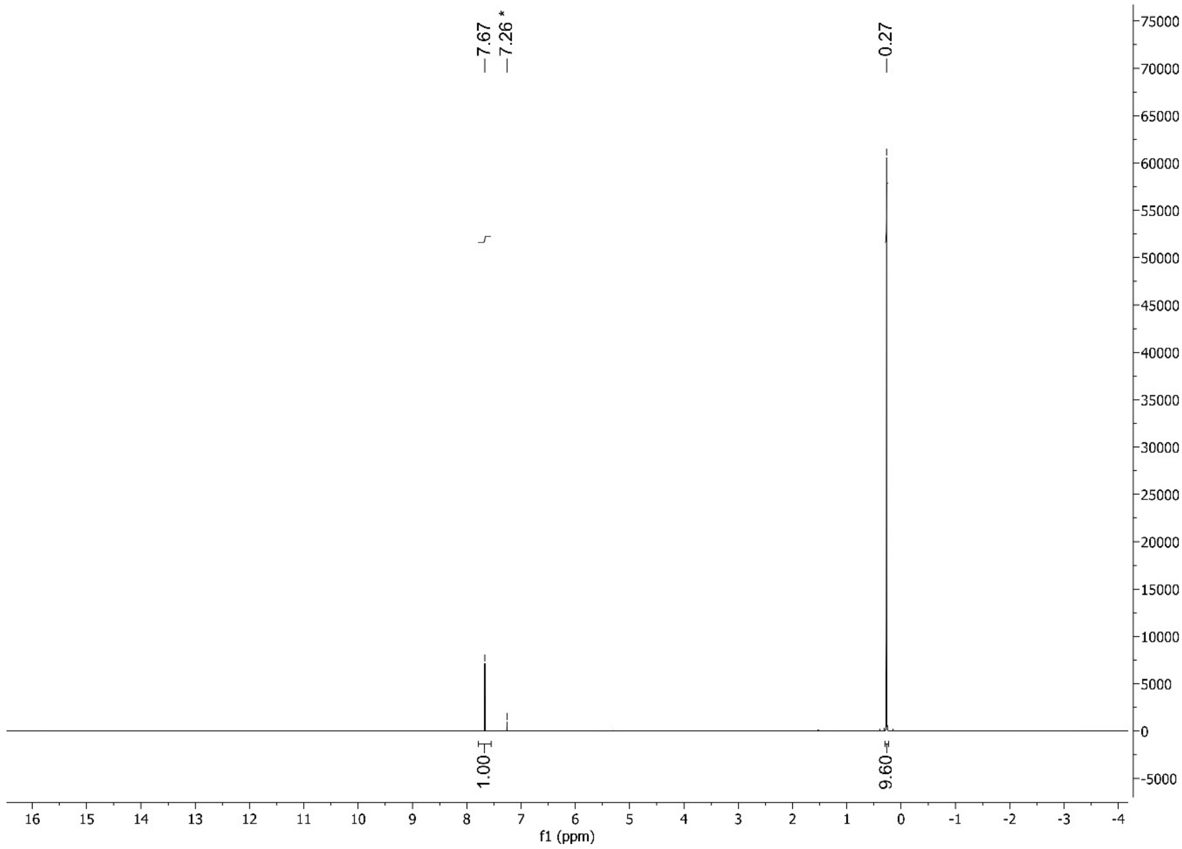

6 in $CDCl_3$, ^{13}C NMR (303 K, 125 MHz)

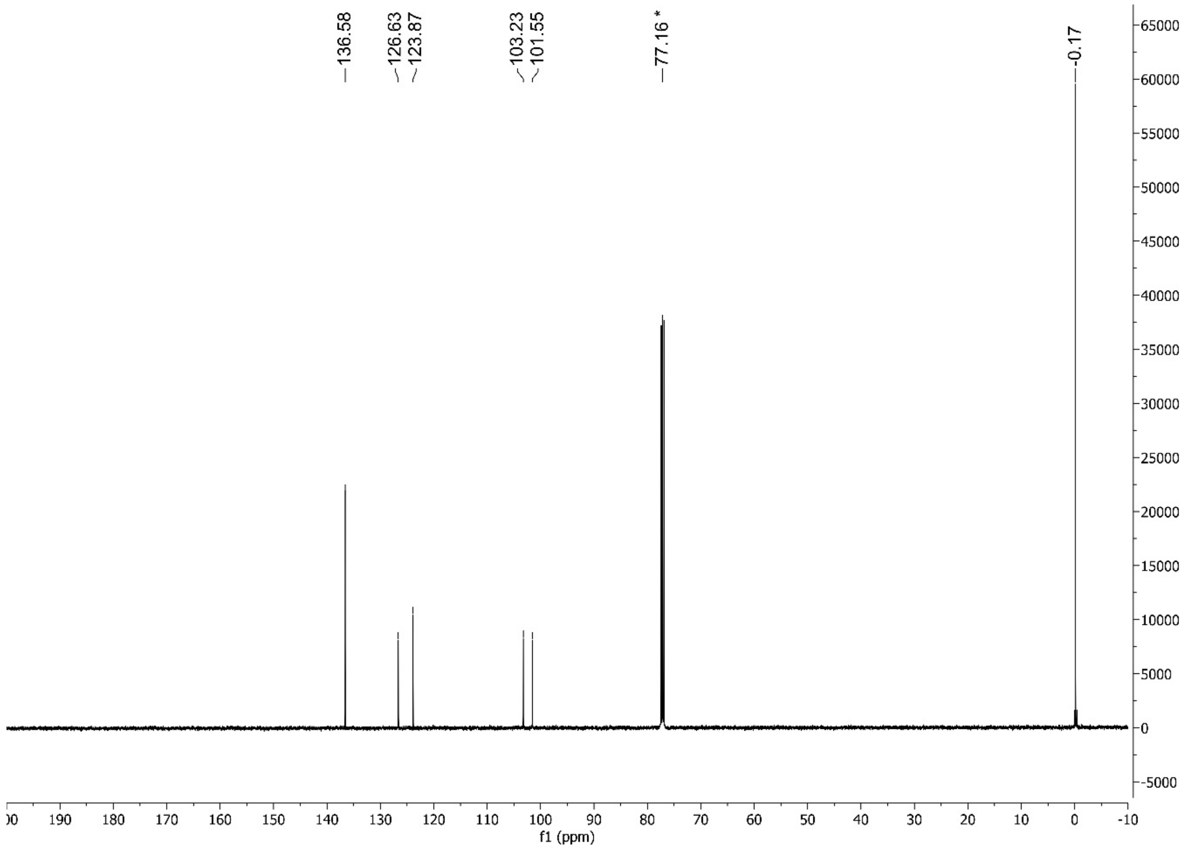

11a in $CDCl_3$, ^{1}H NMR (303 K, 500 MHz)

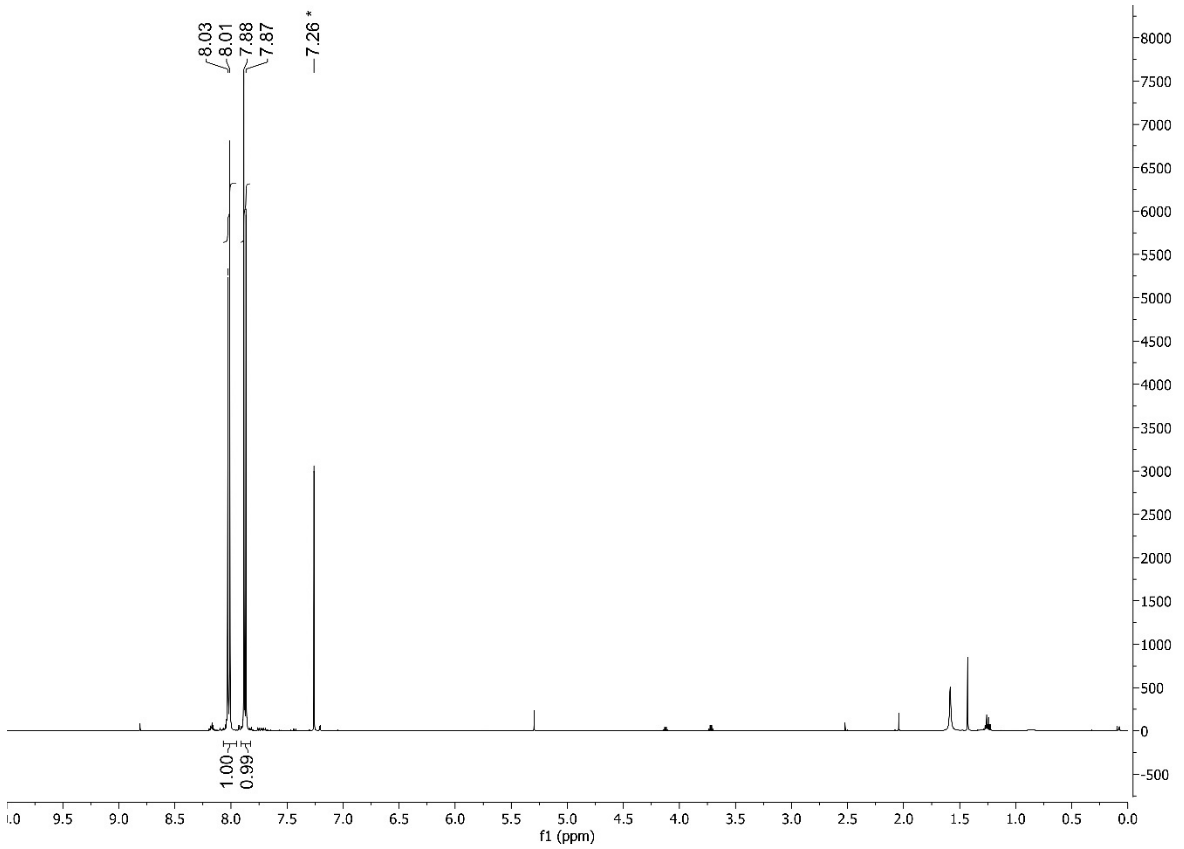

11a in $CDCl_3$, ^{13}C NMR (303 K, 125 MHz)

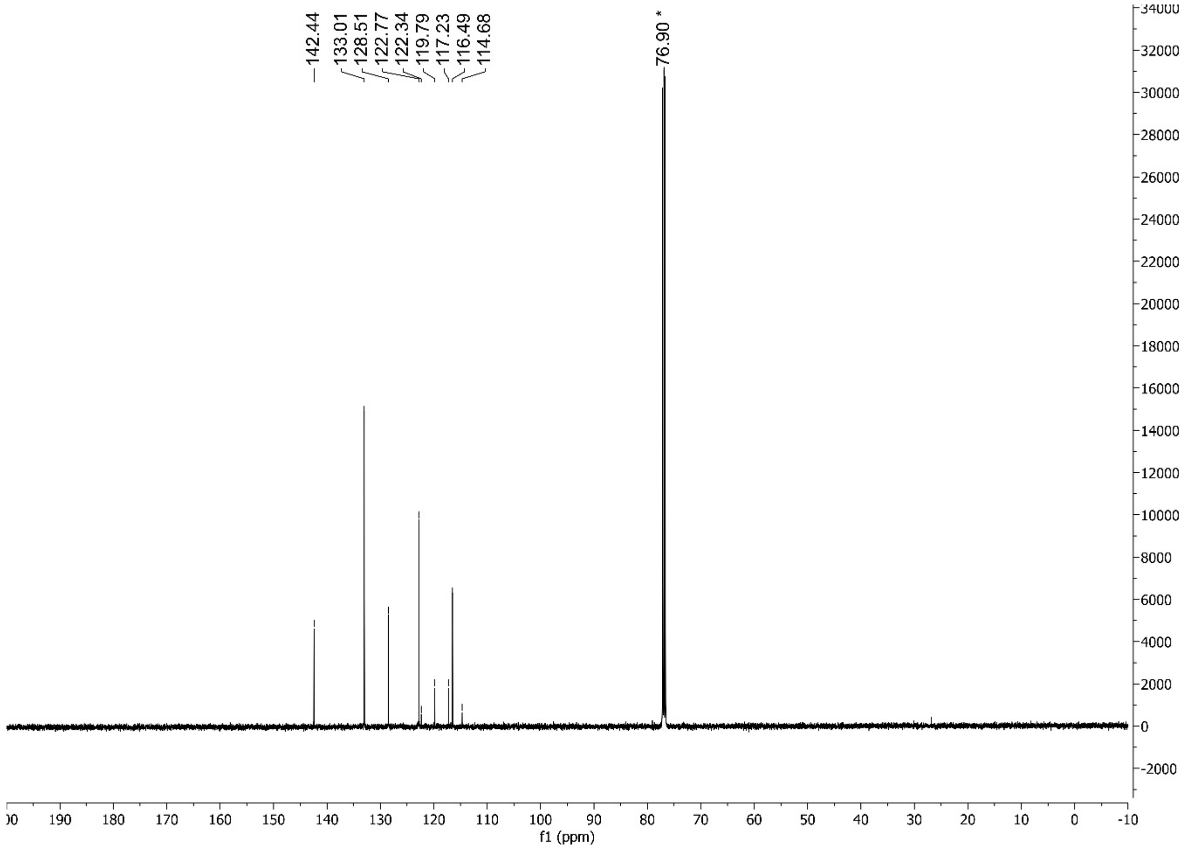

11b in $CDCl_3$, ^1H NMR (303 K, 500 MHz)

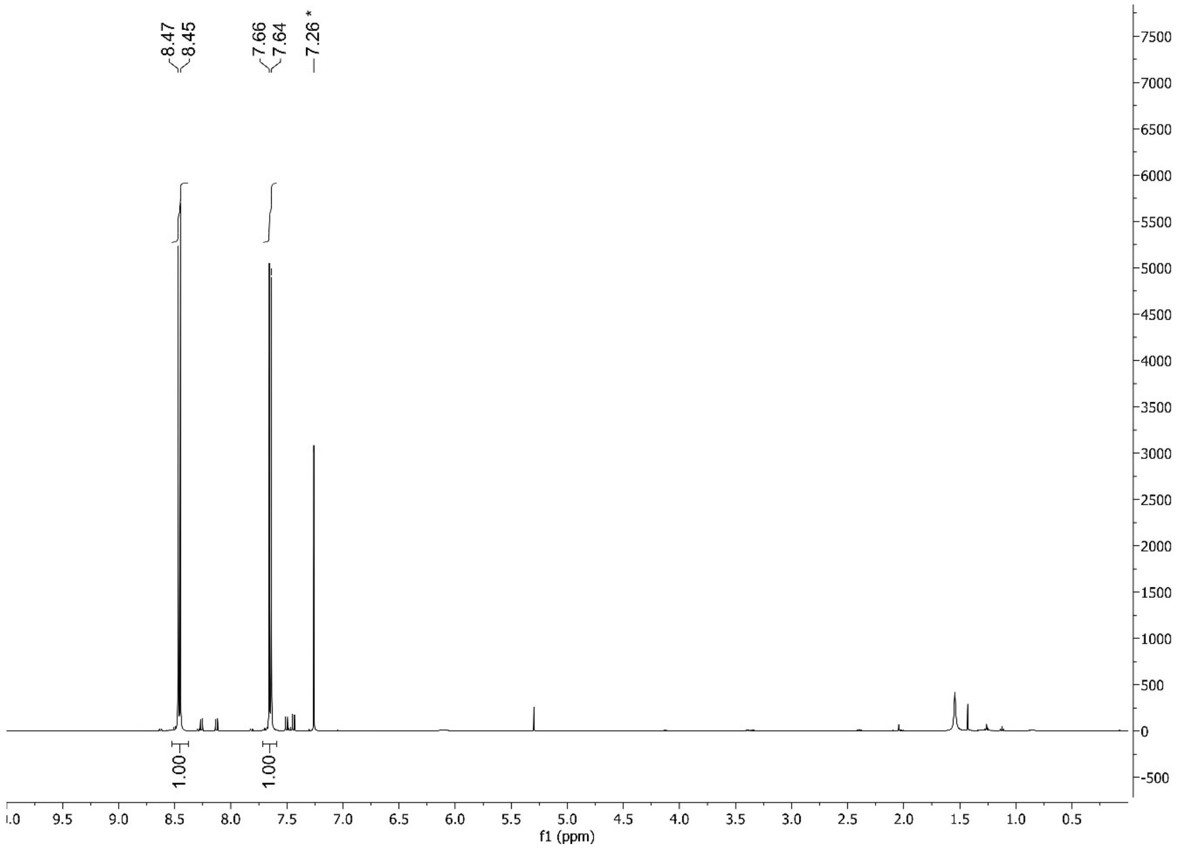

11b in $CDCl_3$, ^{13}C NMR (303 K, 125 MHz)

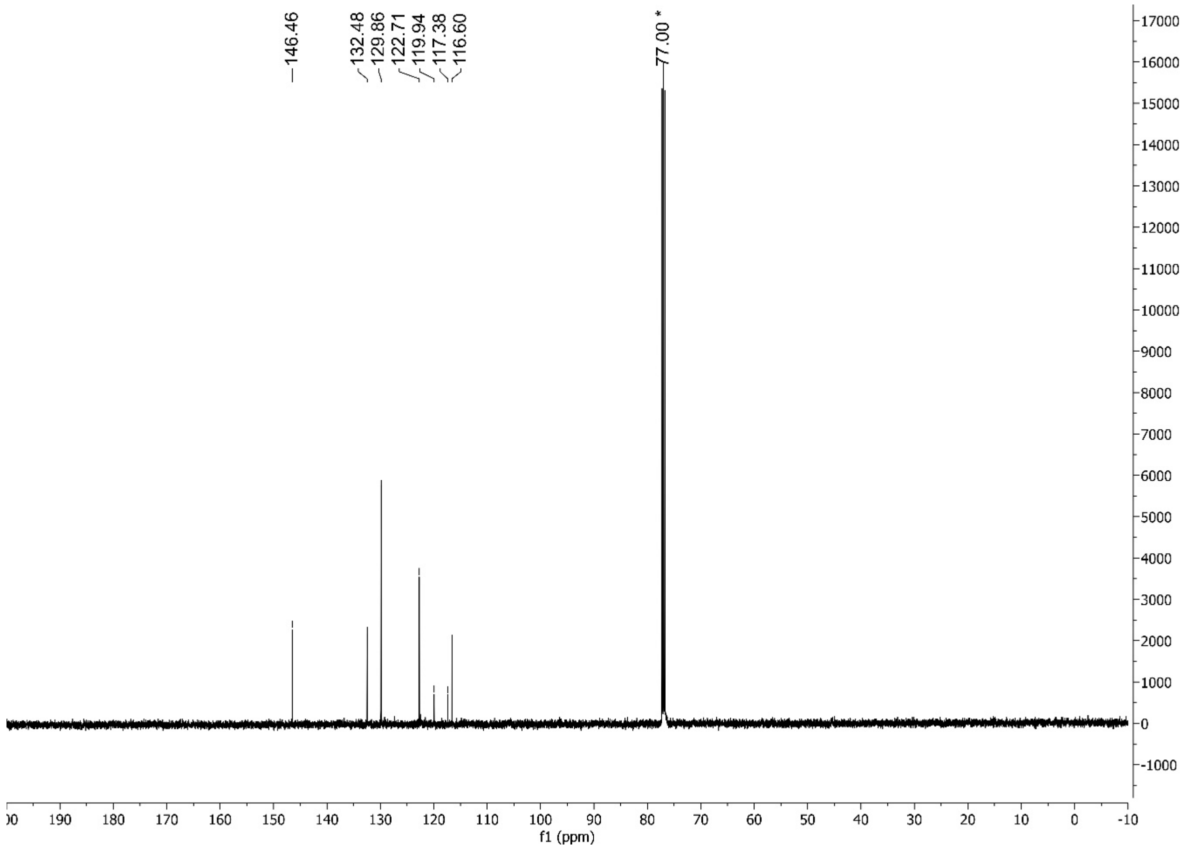

12a in $CDCl_3$, ^1H NMR (303 K, 500 MHz)

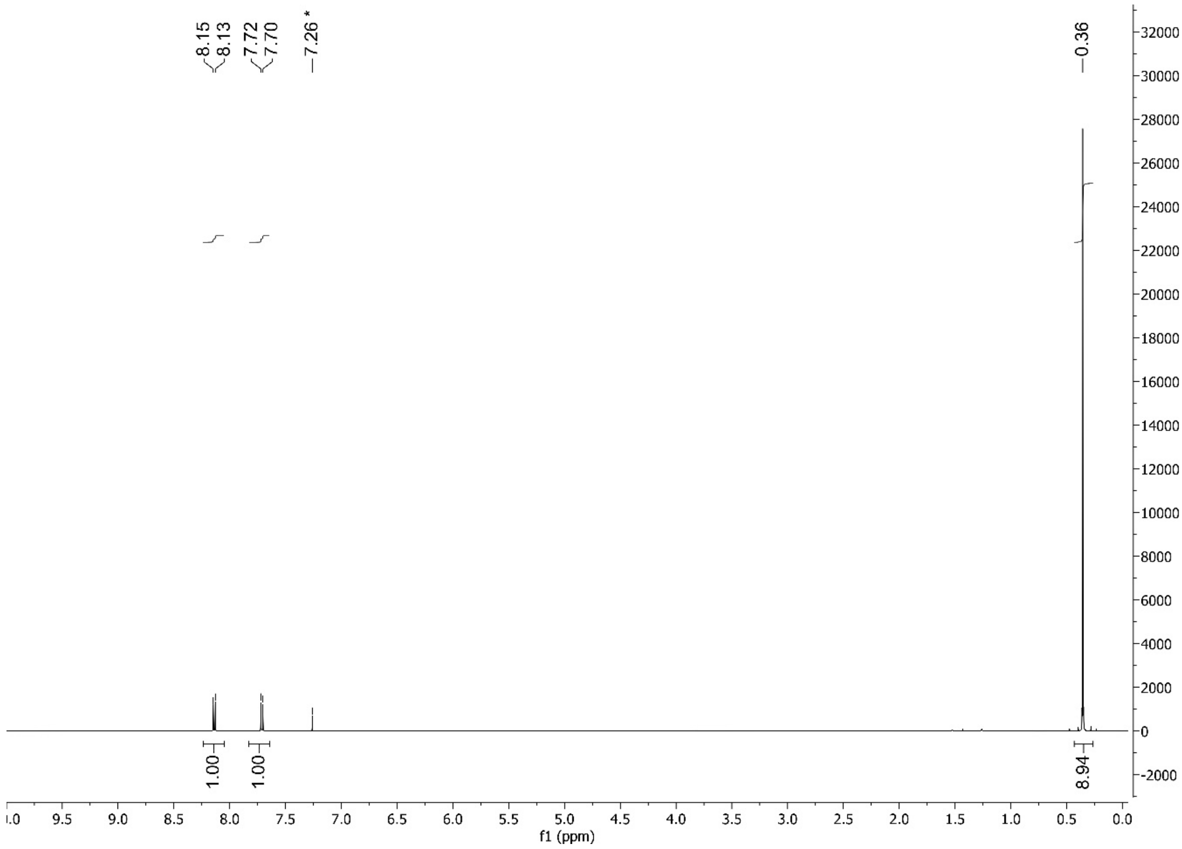

12a in $CDCl_3$, ^{13}C NMR (303 K, 125 MHz)

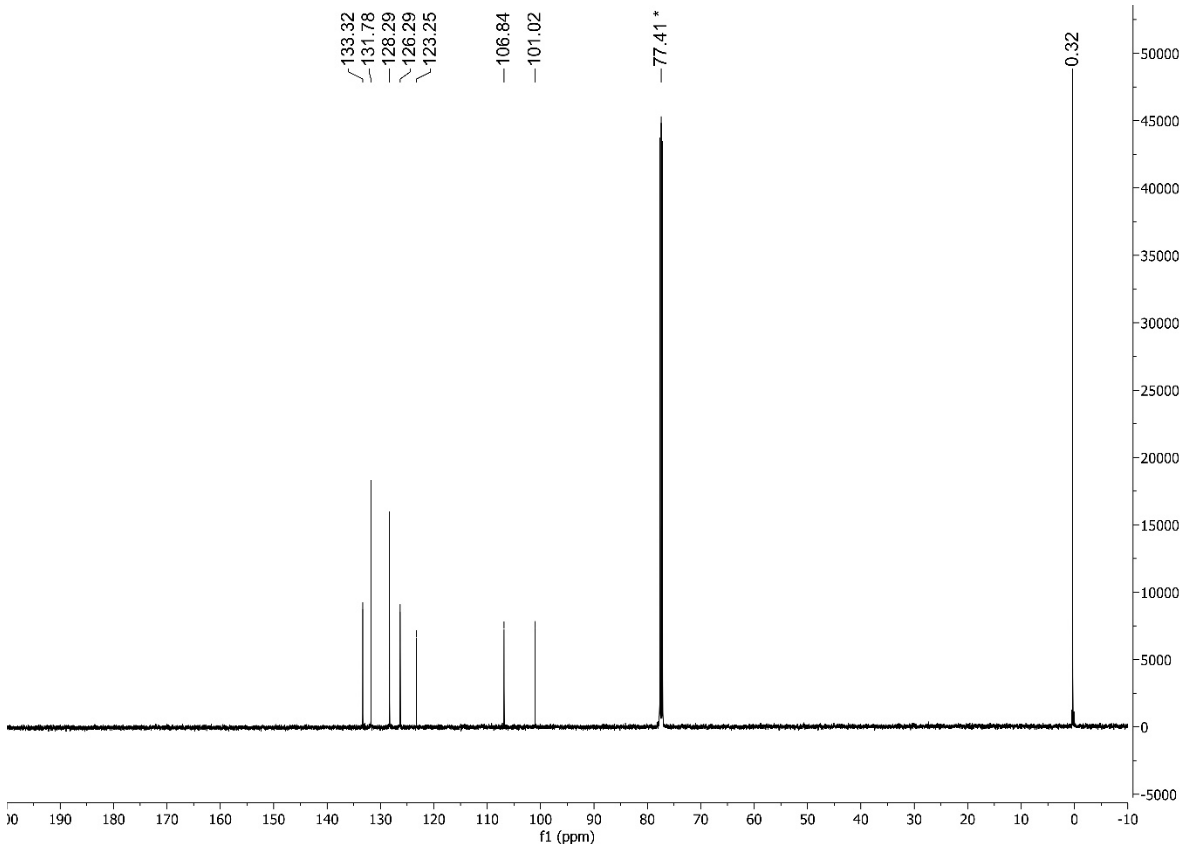

12b in $CDCl_3$, ^1H NMR (303 K, 500 MHz)

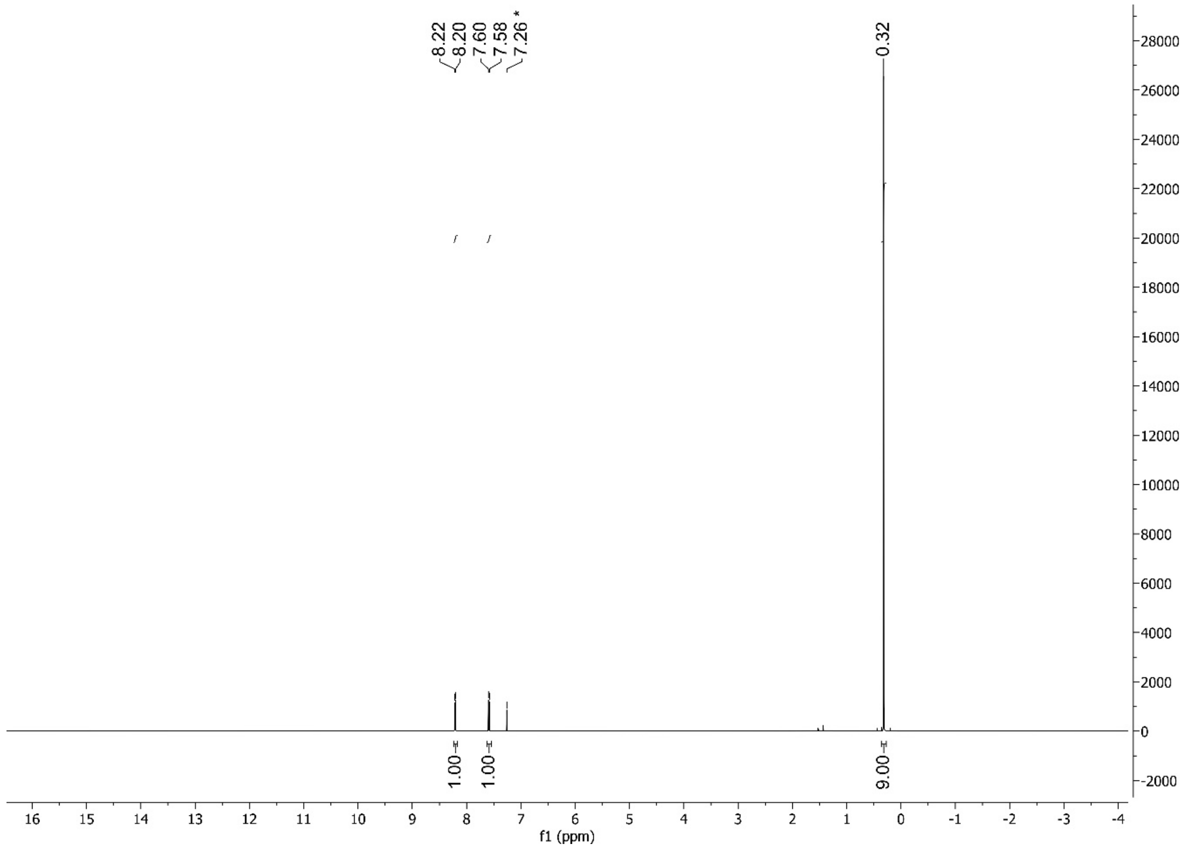

12b in $CDCl_3$, ^{13}C NMR (303 K, 125 MHz)

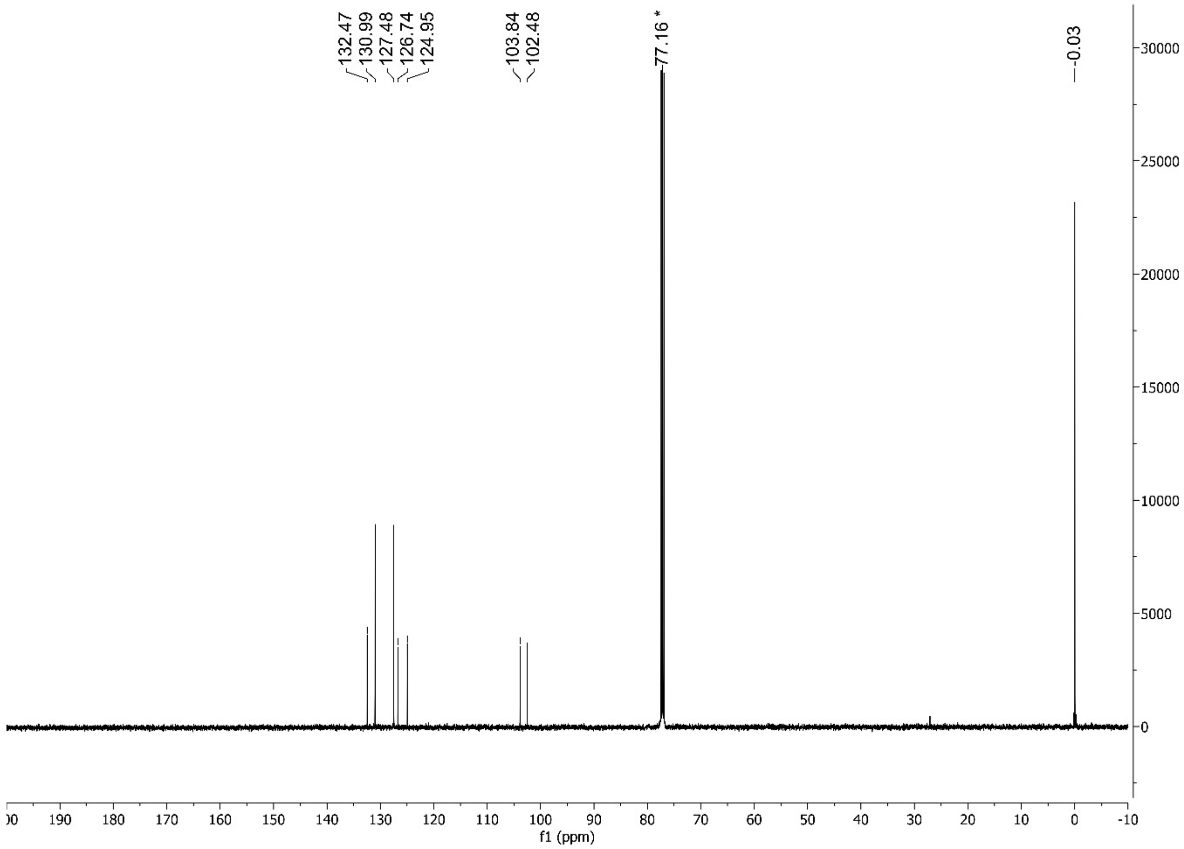

SI3 in DMSO, ^{1}H NMR (303 K, 500 MHz)

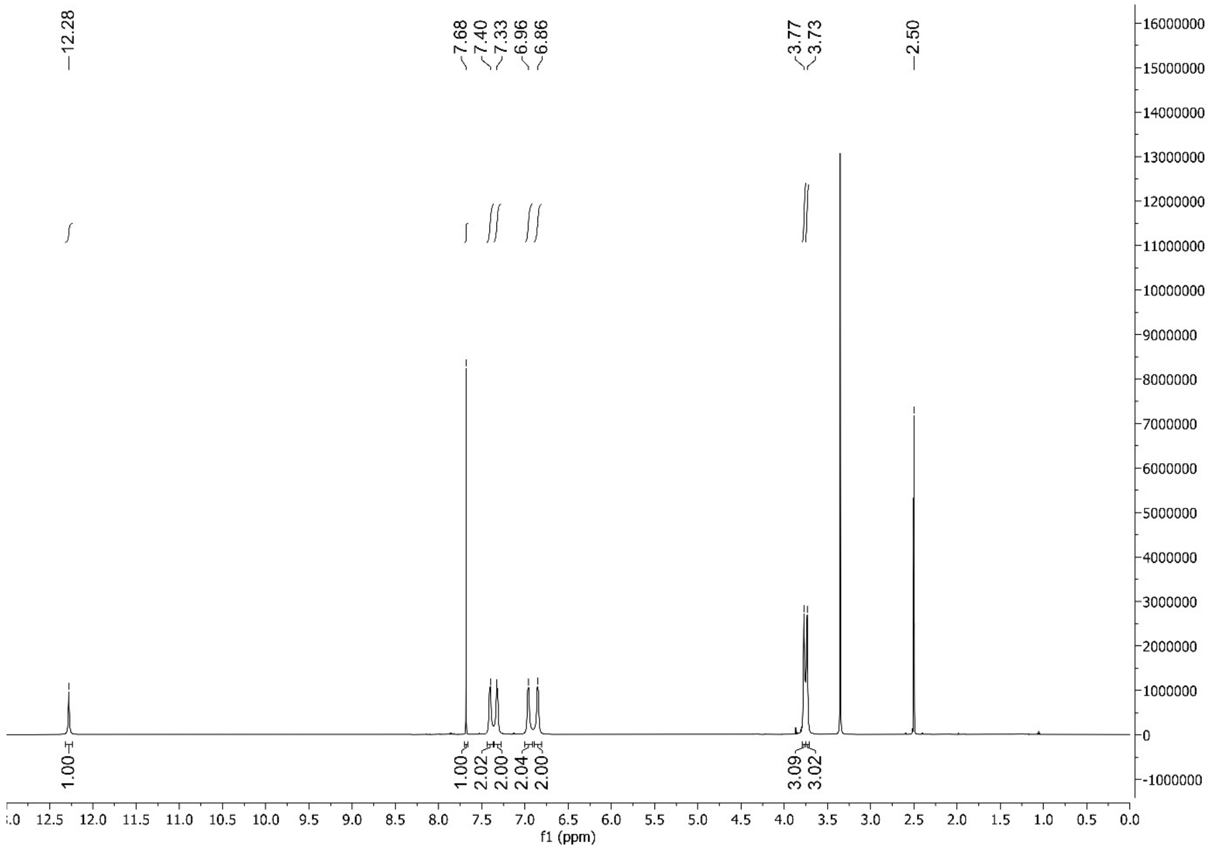

SI3 in DMSO, ^{13}C NMR (303 K, 125 MHz)

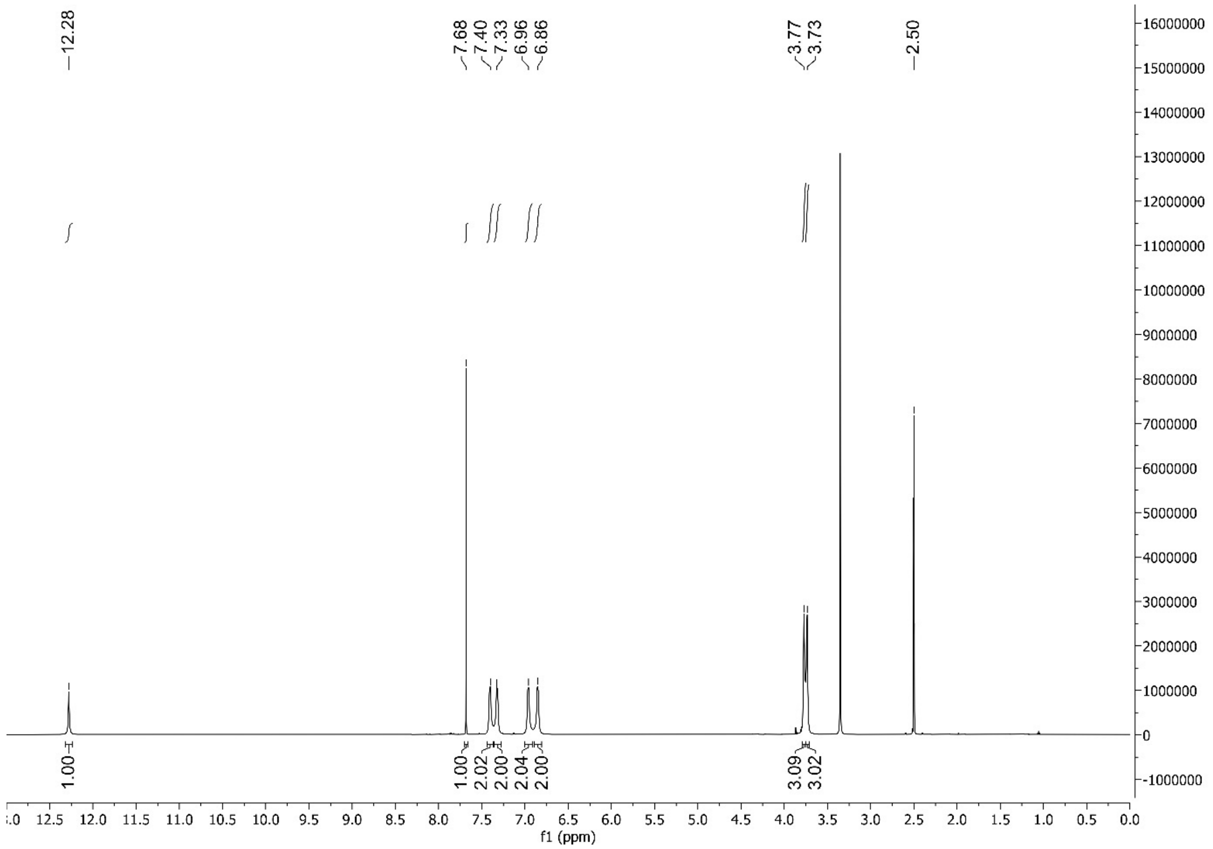

SI4 in $CDCl_3$, ^{1}H NMR (303 K, 500 MHz)

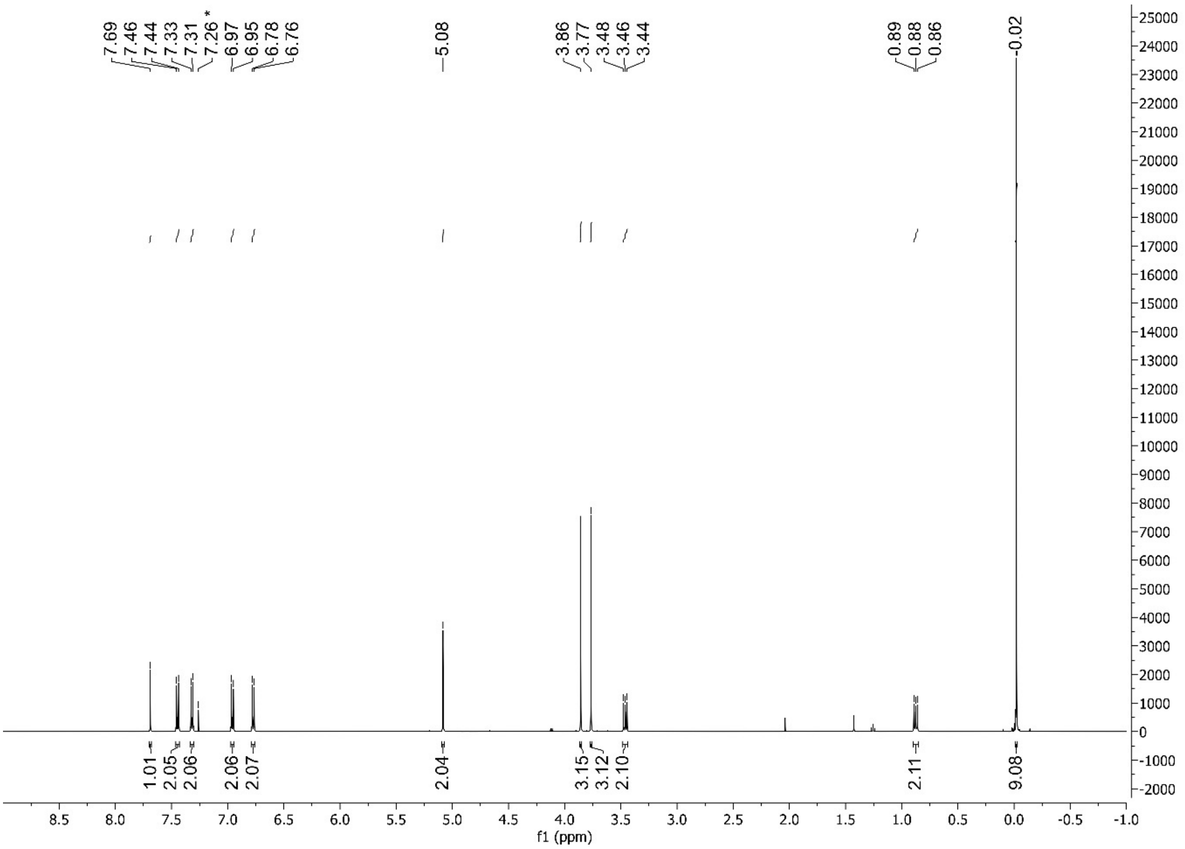

SI4 in $CDCl_3$, ^{13}C NMR (303 K, 125 MHz)

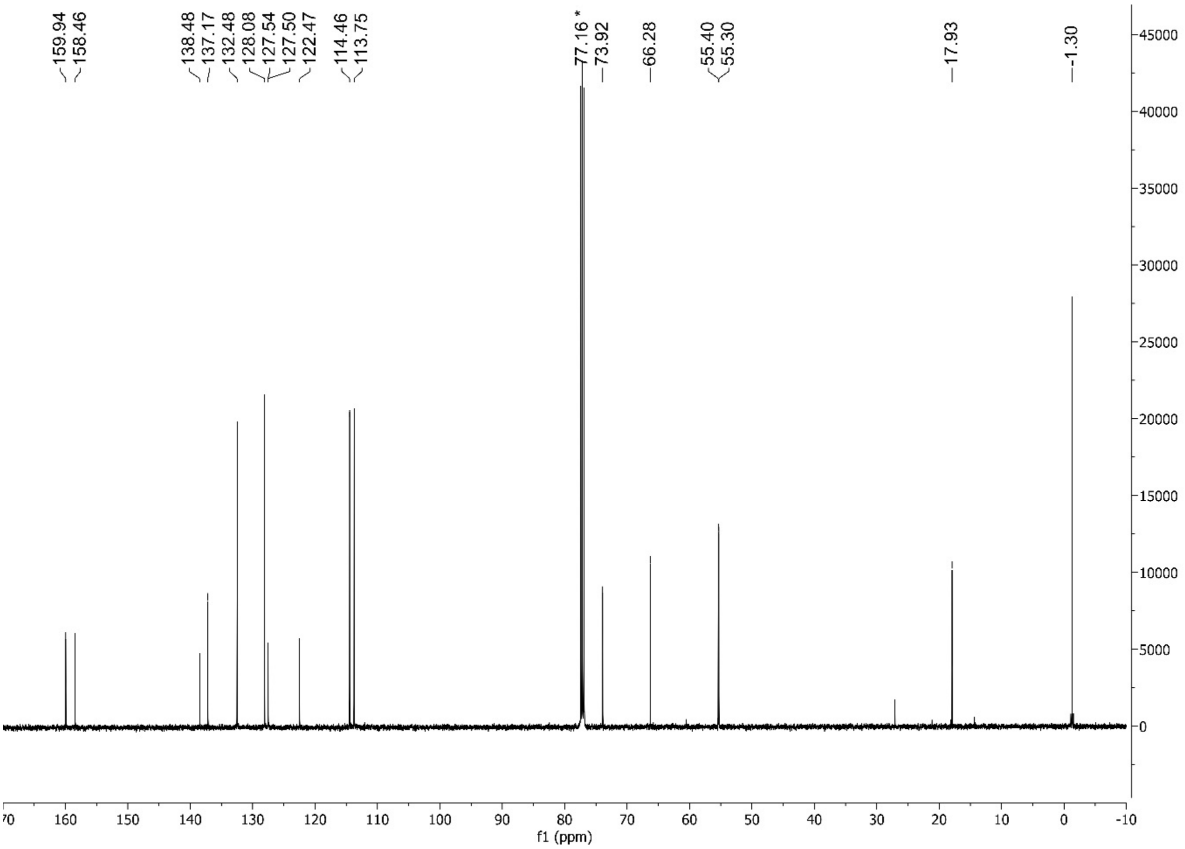

7 in $CDCl_3$, ^{1}H NMR (303 K, 500 MHz)

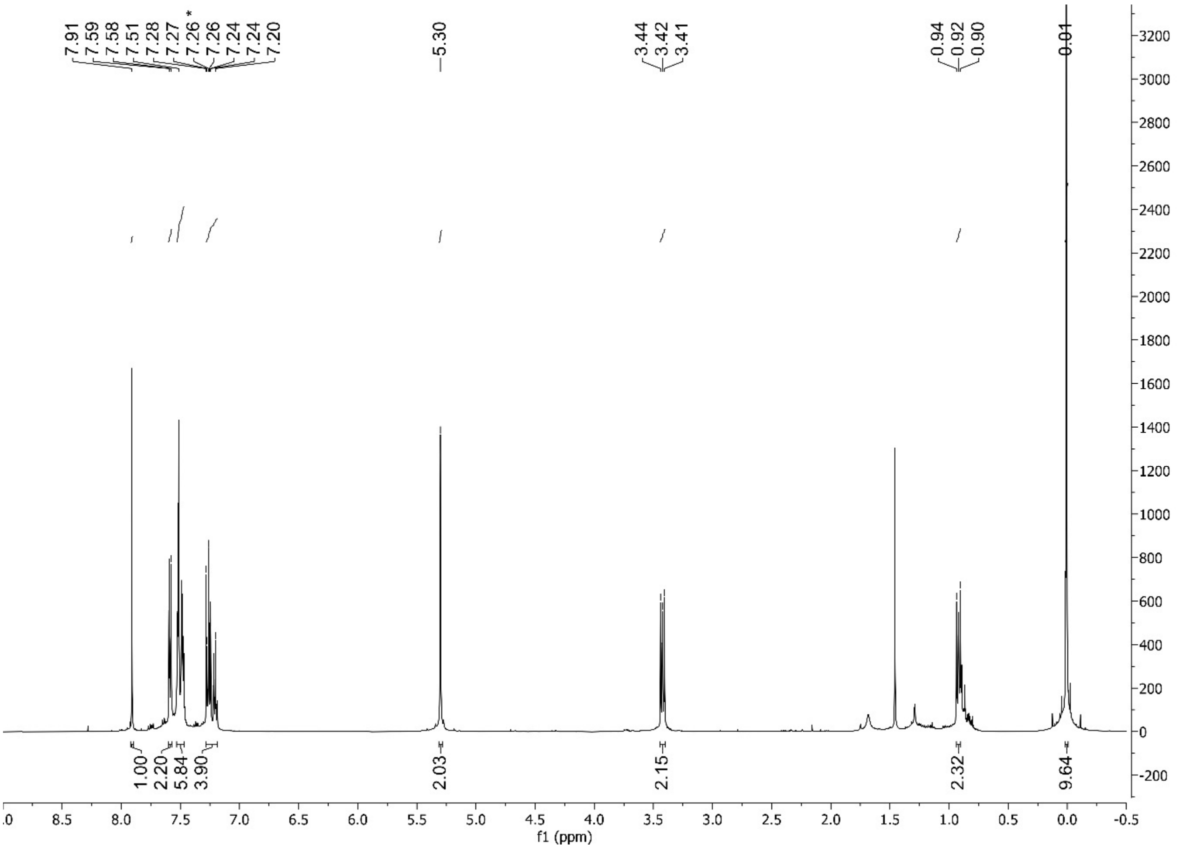

7 in $CDCl_3$, ^{13}C NMR (303 K, 125 MHz)

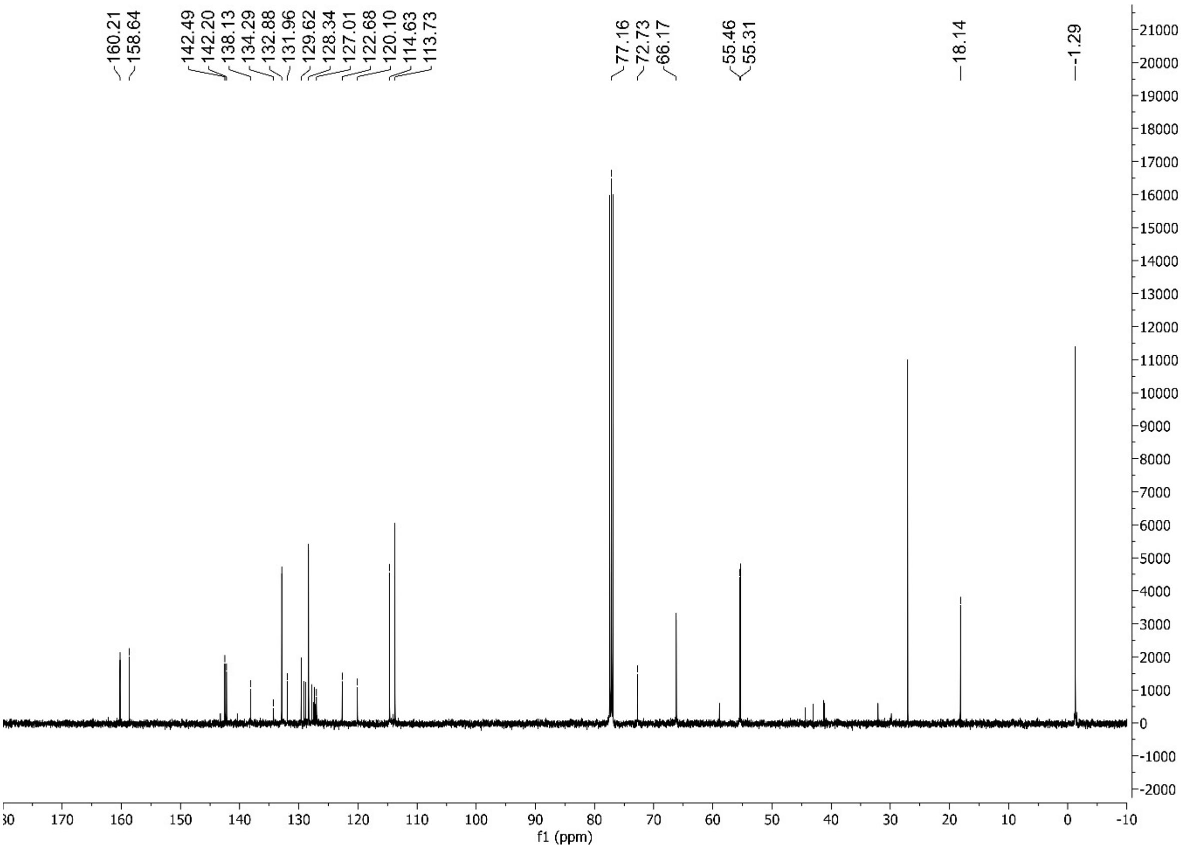

8 in $CDCl_3$, ^{1}H NMR (303 K, 500 MHz)

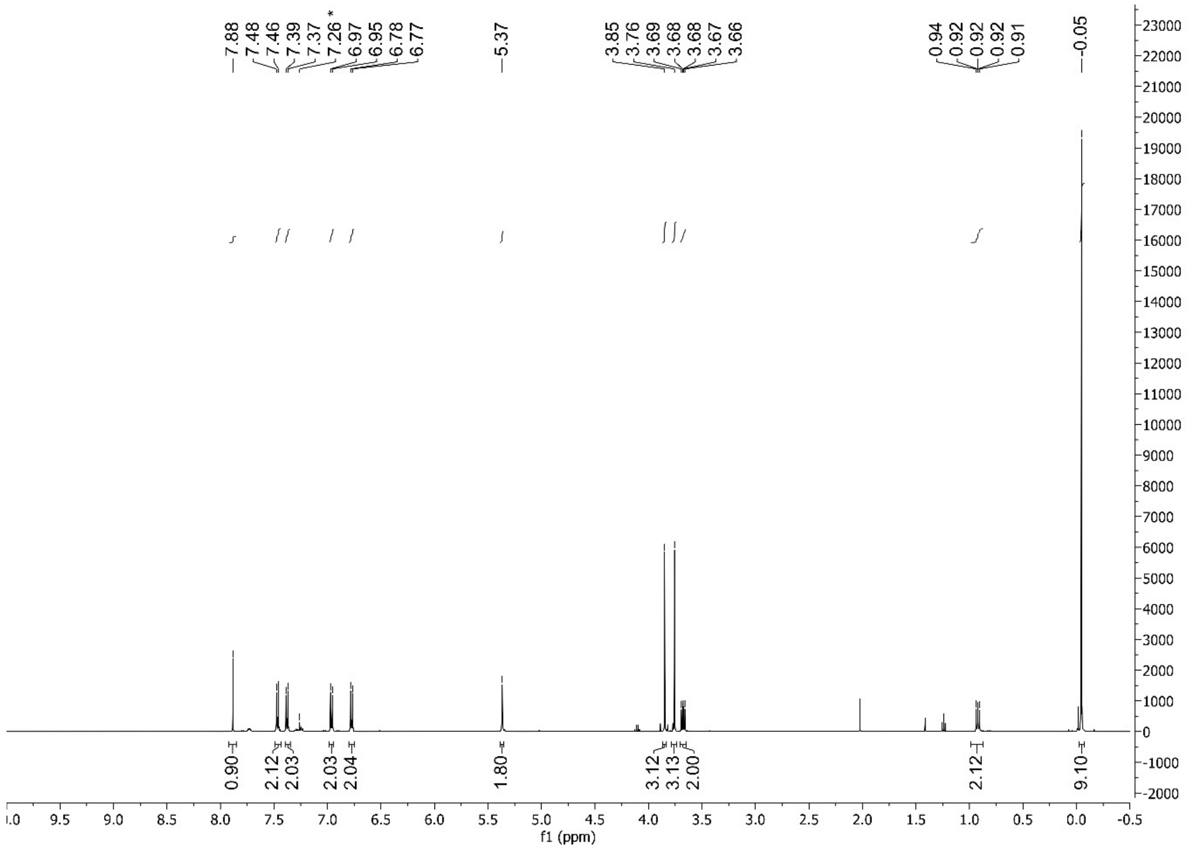

8 in $CDCl_3$, ^{13}C NMR (303 K, 125 MHz)

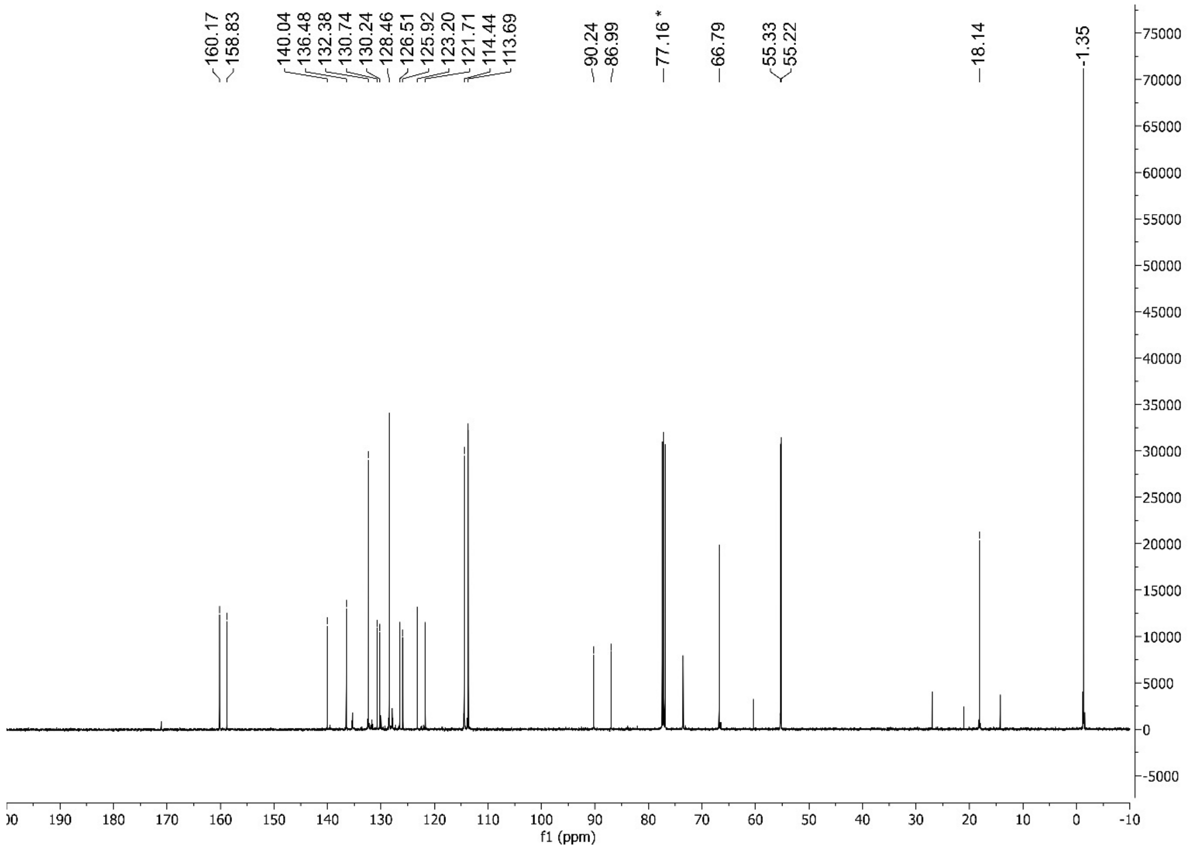

13a in $CDCl_3$, ^1H NMR (303 K, 500 MHz)

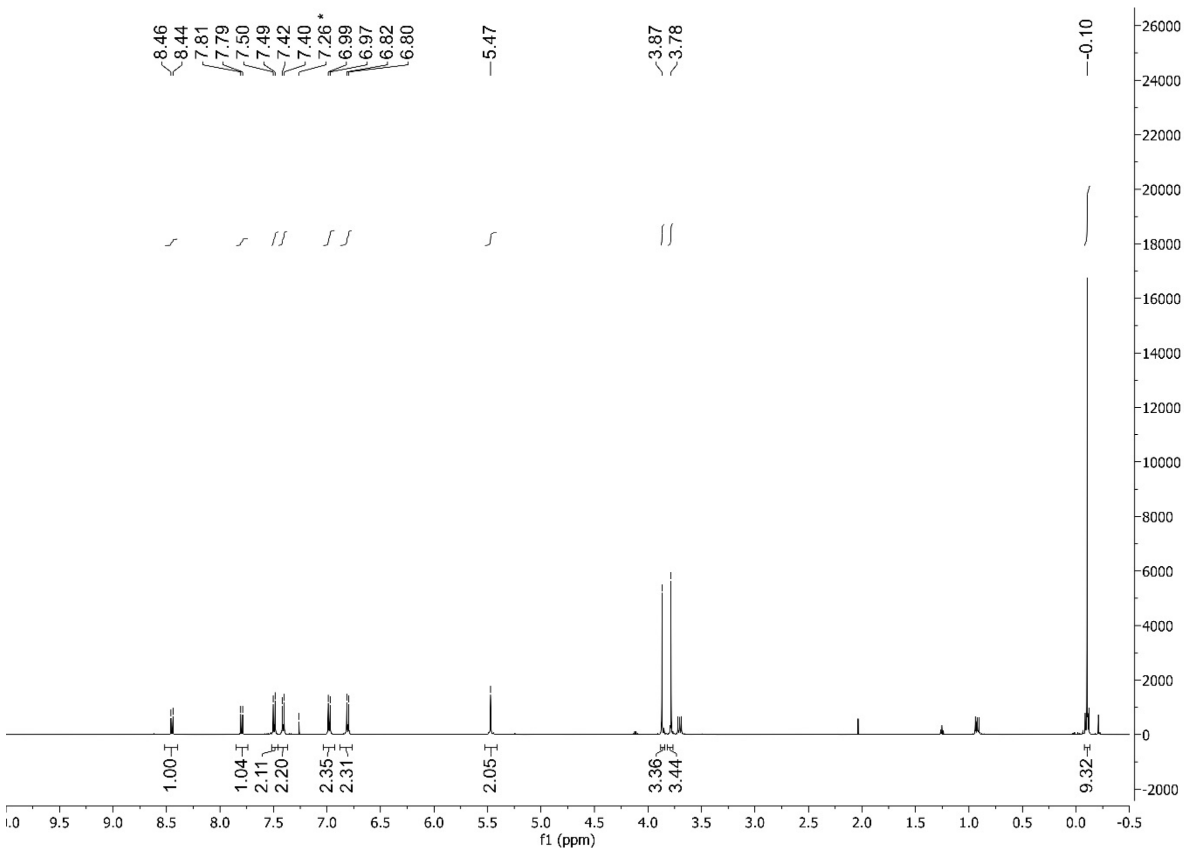

13a in $CDCl_3$, ^{13}C NMR (303 K, 125 MHz)

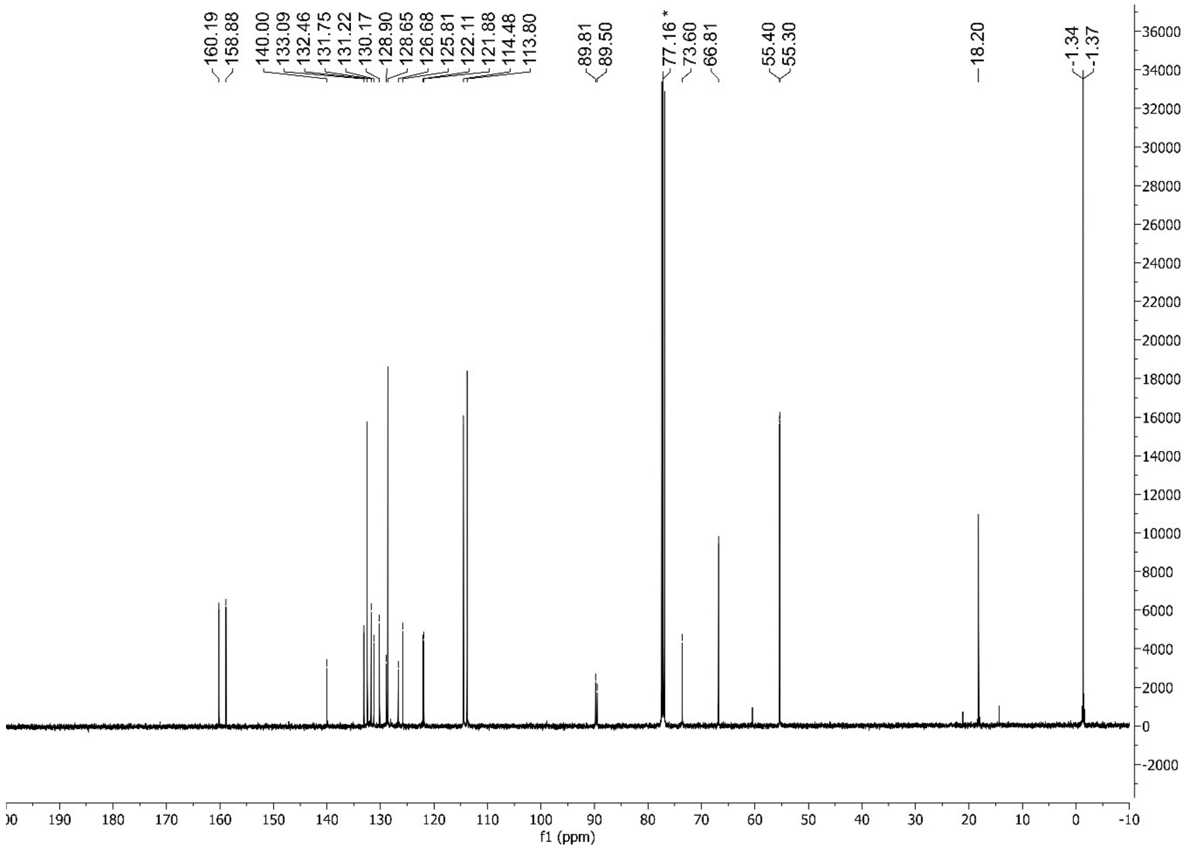

13b in $CDCl_3$, ^1H NMR (303 K, 500 MHz)

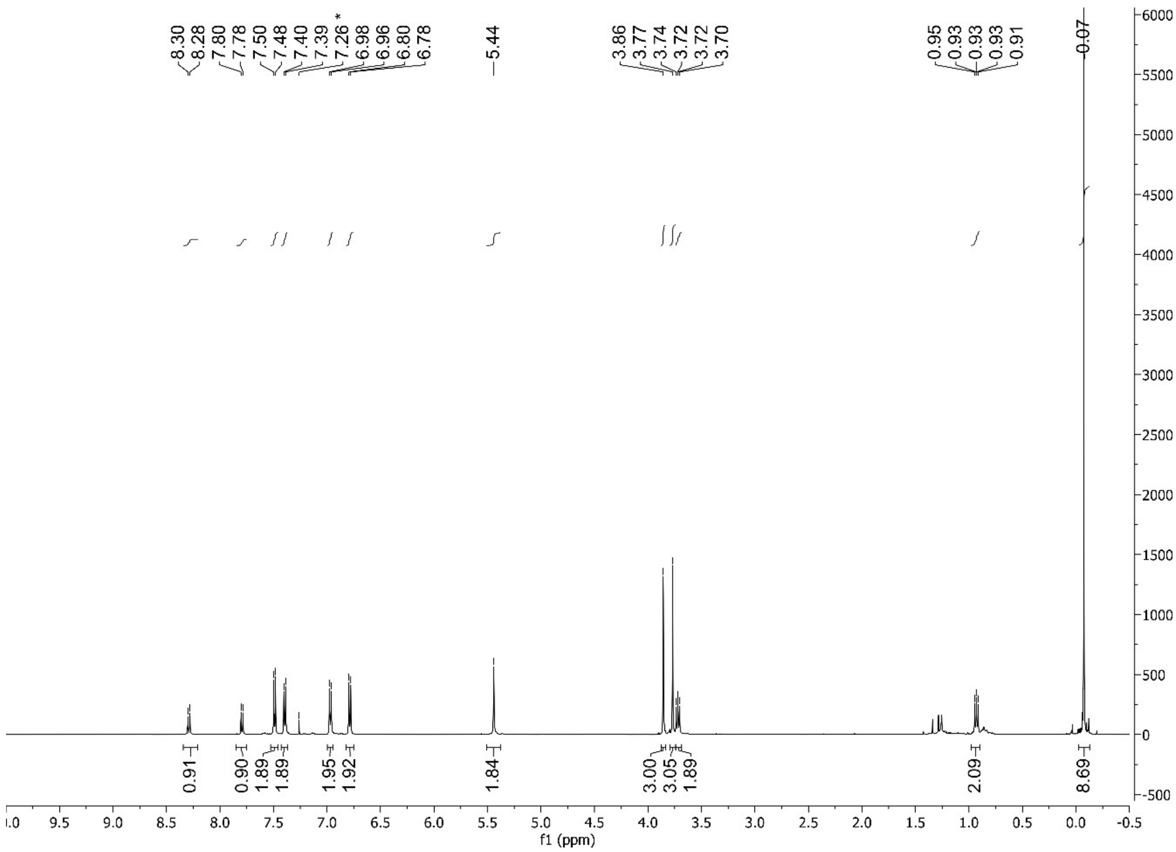

13b in $CDCl_3$, ^{13}C NMR (303 K, 125 MHz)

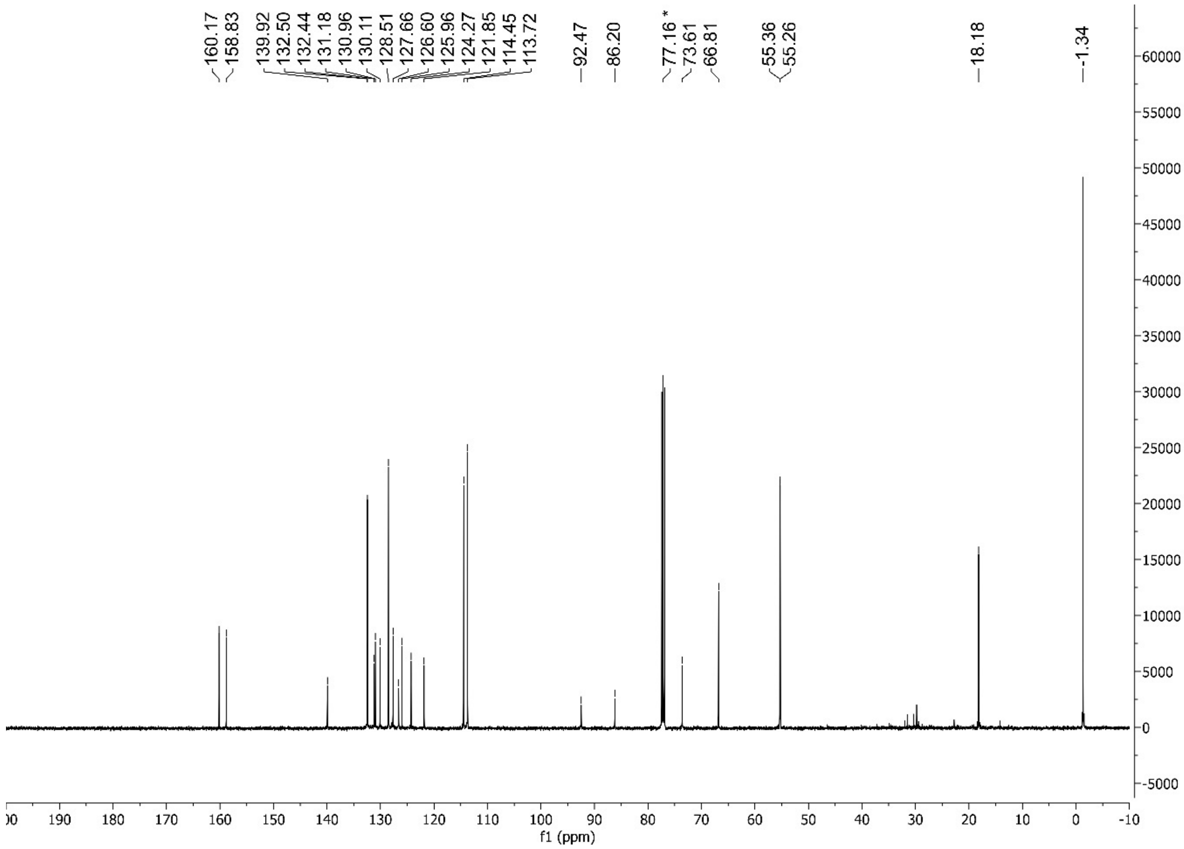

3 in CDCl3, ^{1}H NMR (303 K, 500 MHz)

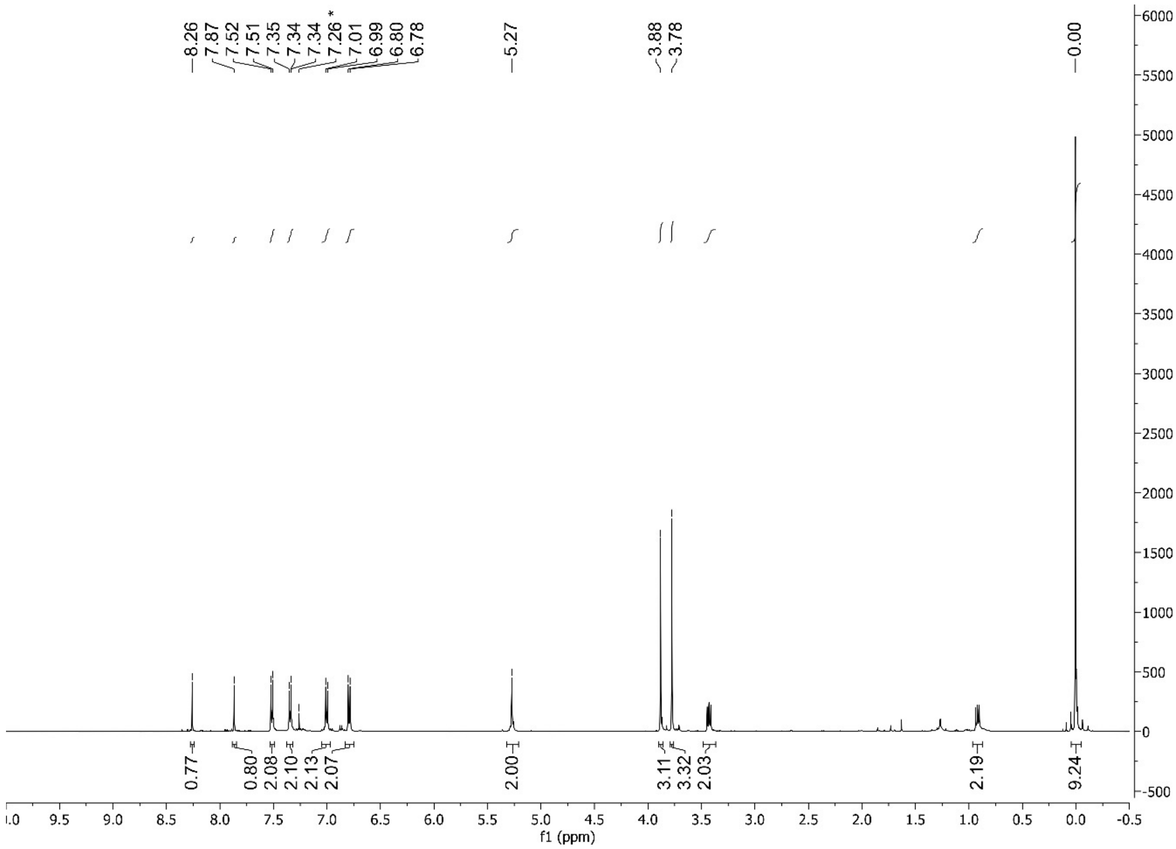

3 in CDCl3, ^{13}C NMR (303 K, 125 MHz)

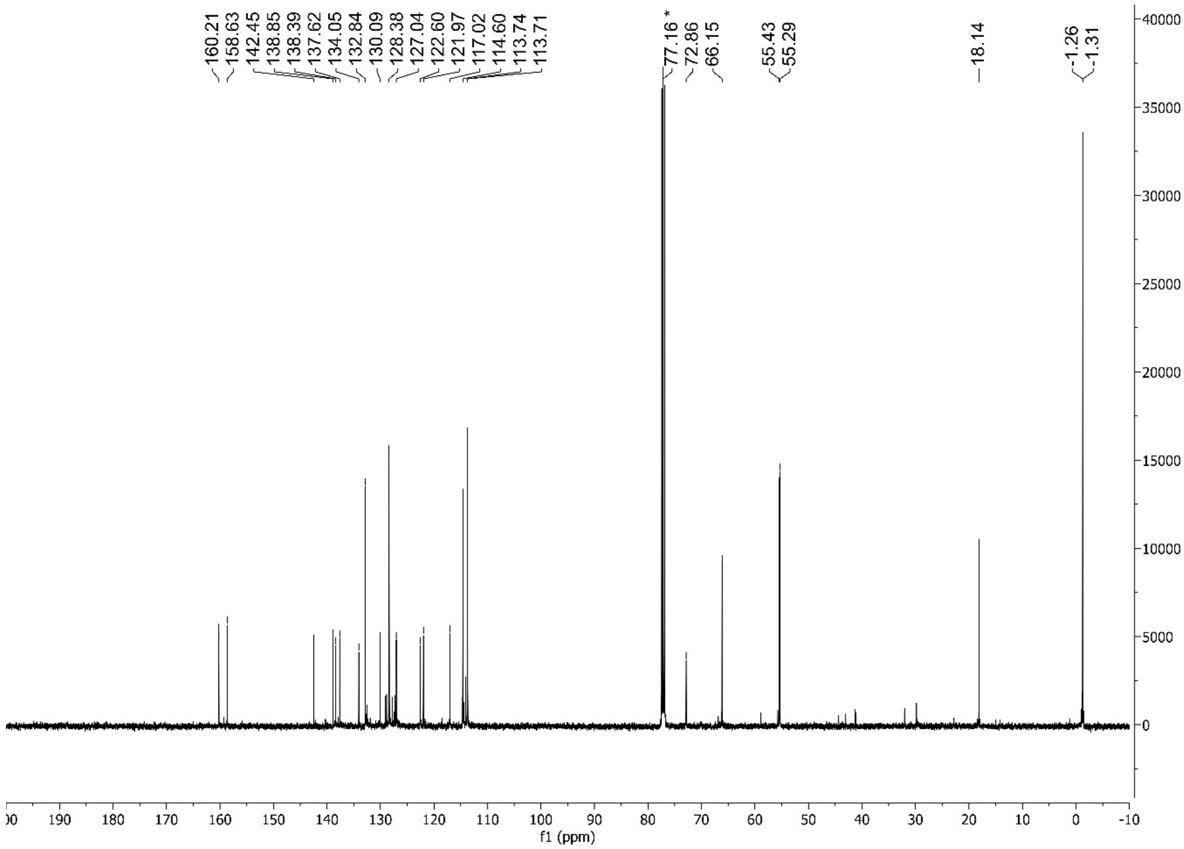

9a in $CDCl_3$, ^{1}H NMR (303 K, 500 MHz)

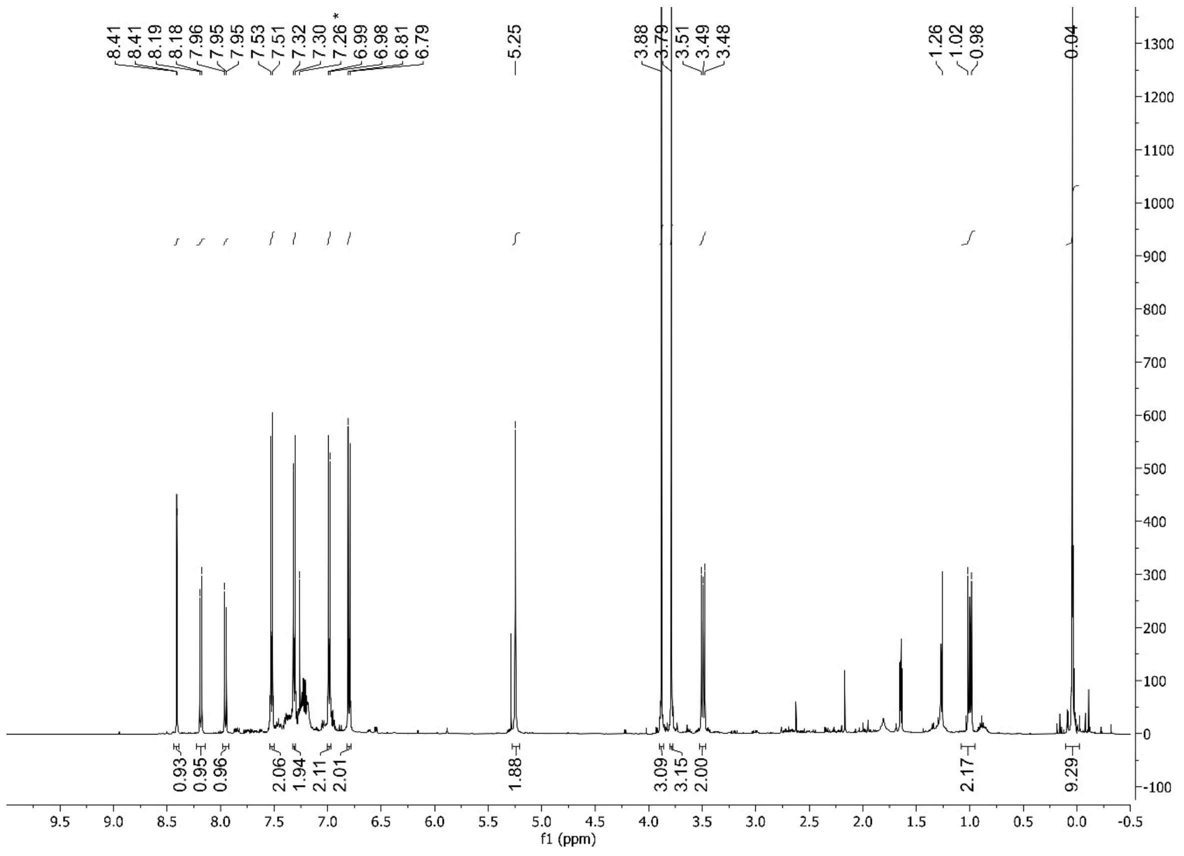

9a in $CDCl_3$, ^{13}C NMR (303 K, 125 MHz)

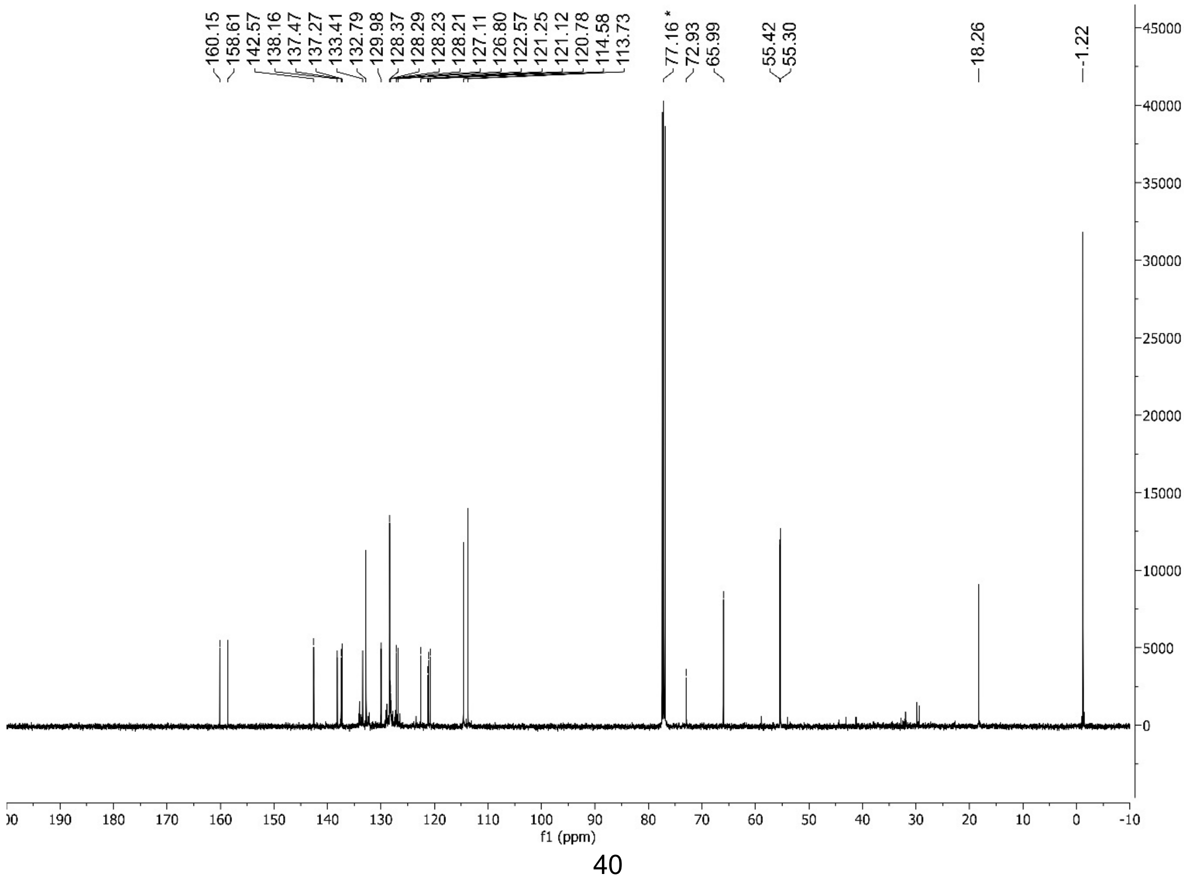

9b in $CDCl_3$, ^1H NMR (303 K, 500 MHz)

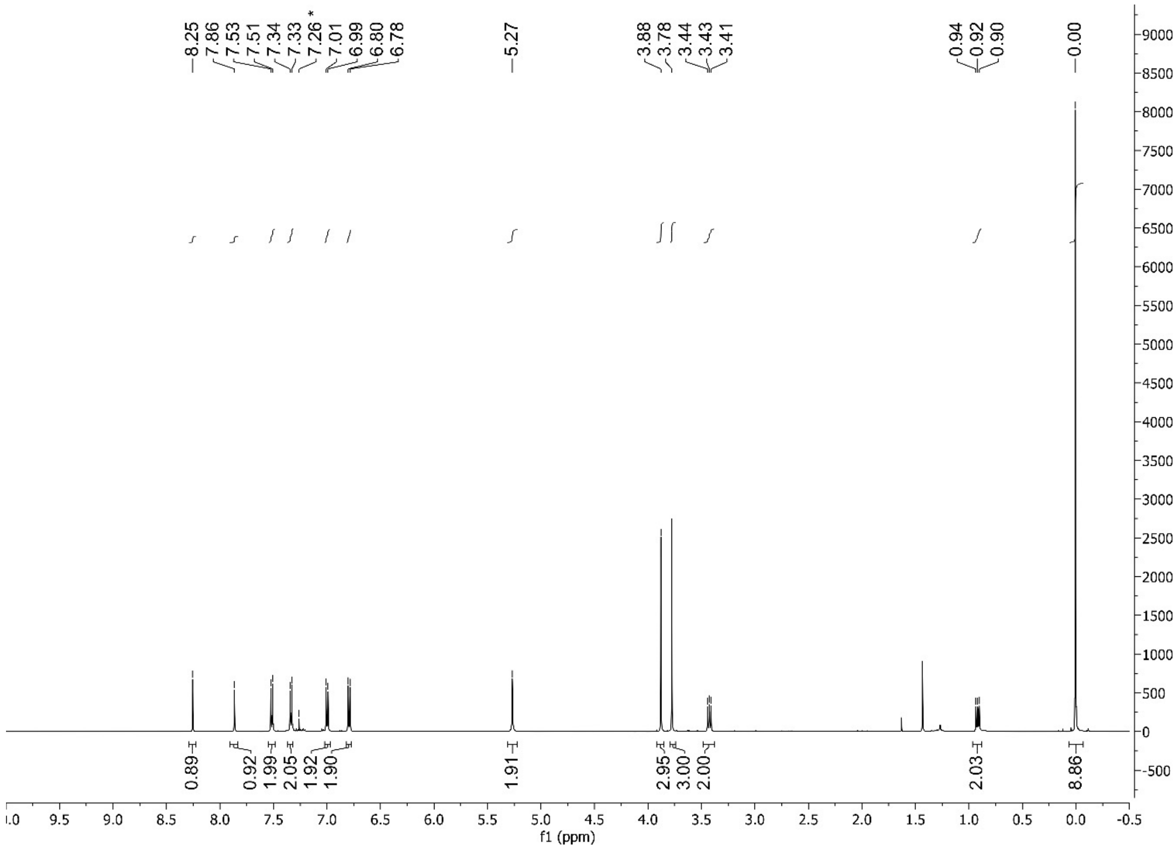

9b in $CDCl_3$, ^{13}C NMR (303 K, 125 MHz)

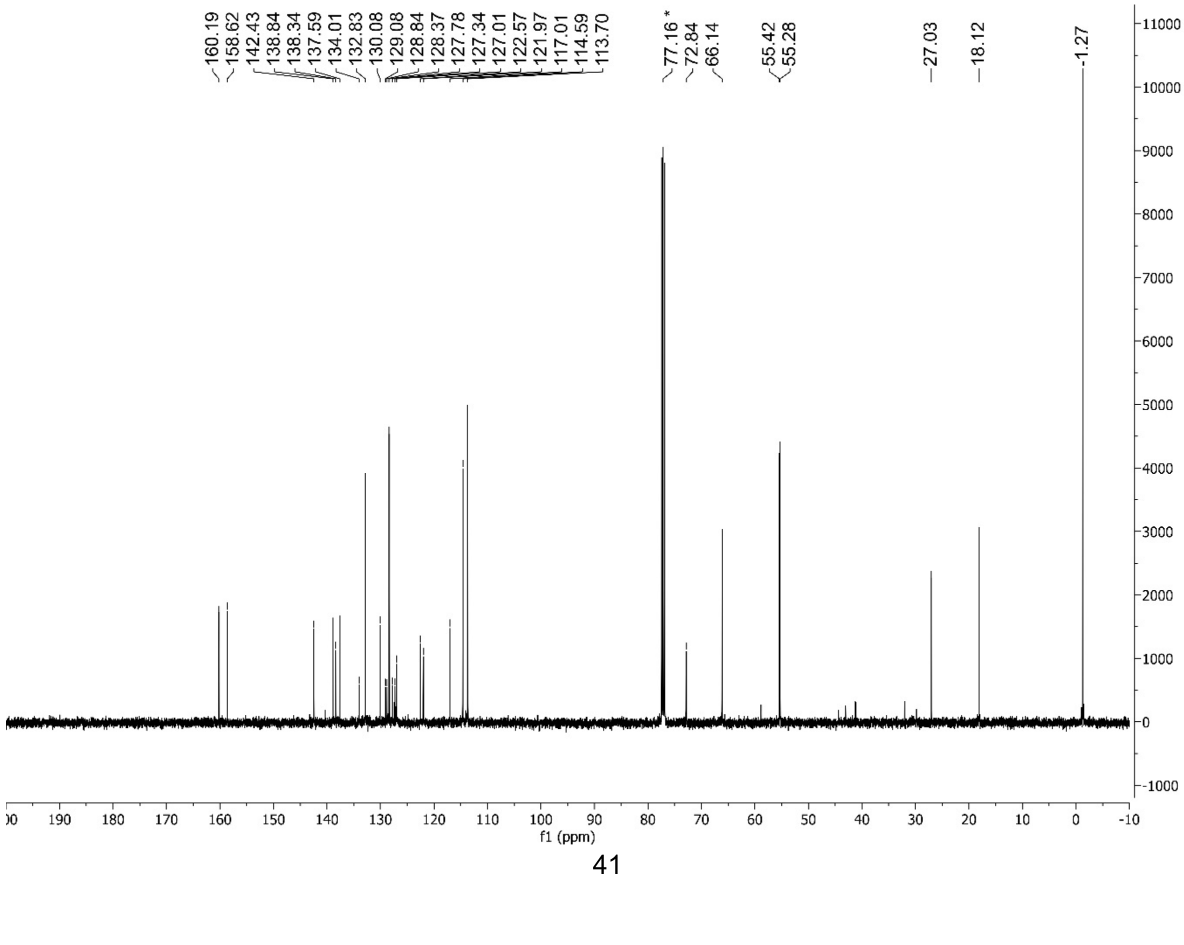

3.2. Mass spectra

SI1 MS (ESI) *m/z*: calc. $[C_{38}H_{33}N_4O_4S]^+$ = 641.2218, found: 641.2267

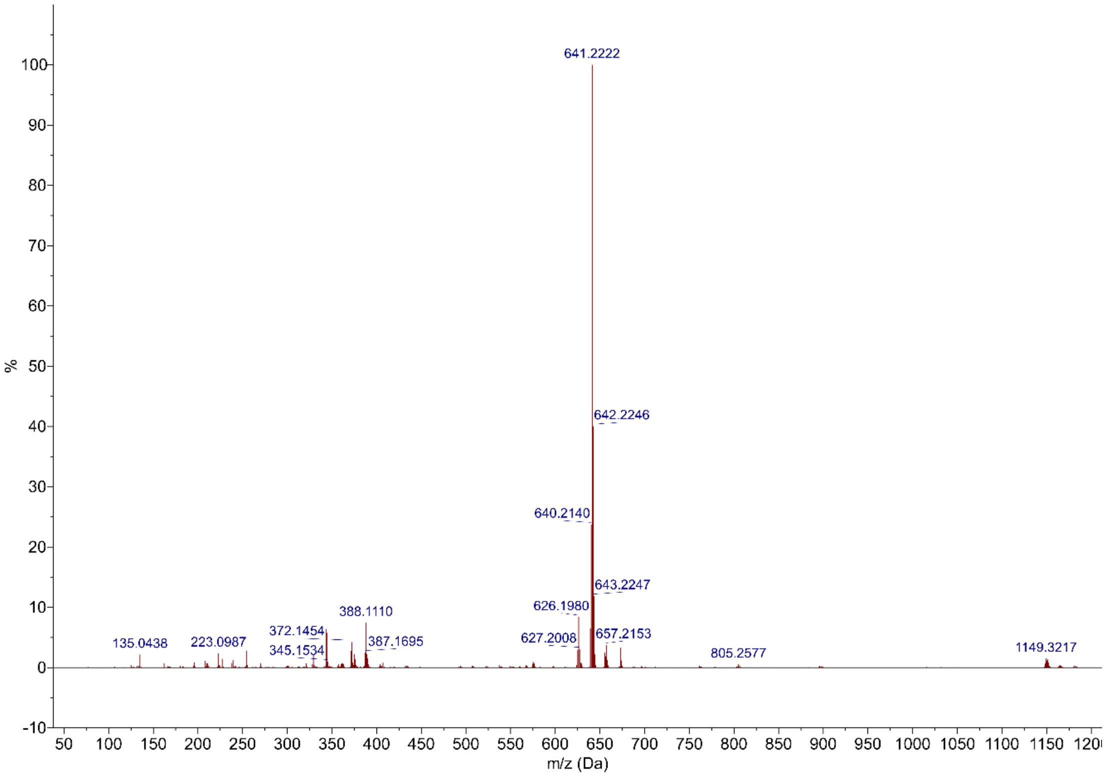

1 MS (ESI) *m/z*: calc. $[C_{38}H_{31}N_4O_4S]^+$ = 639.2061, found: 639.2205

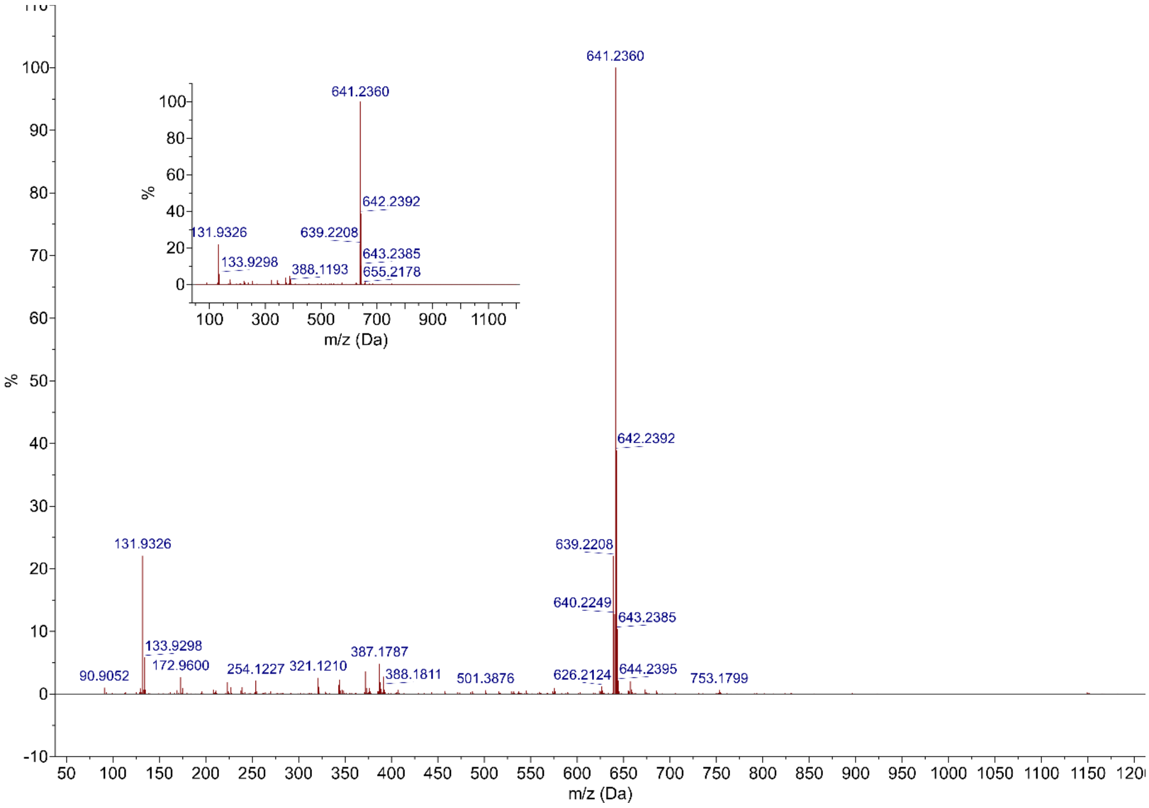

SI2 MS (ESI) *m/z*: calc. $[C_{40}H_{35}N_4O_4]^+$ = 635.2653, found: 635.2668

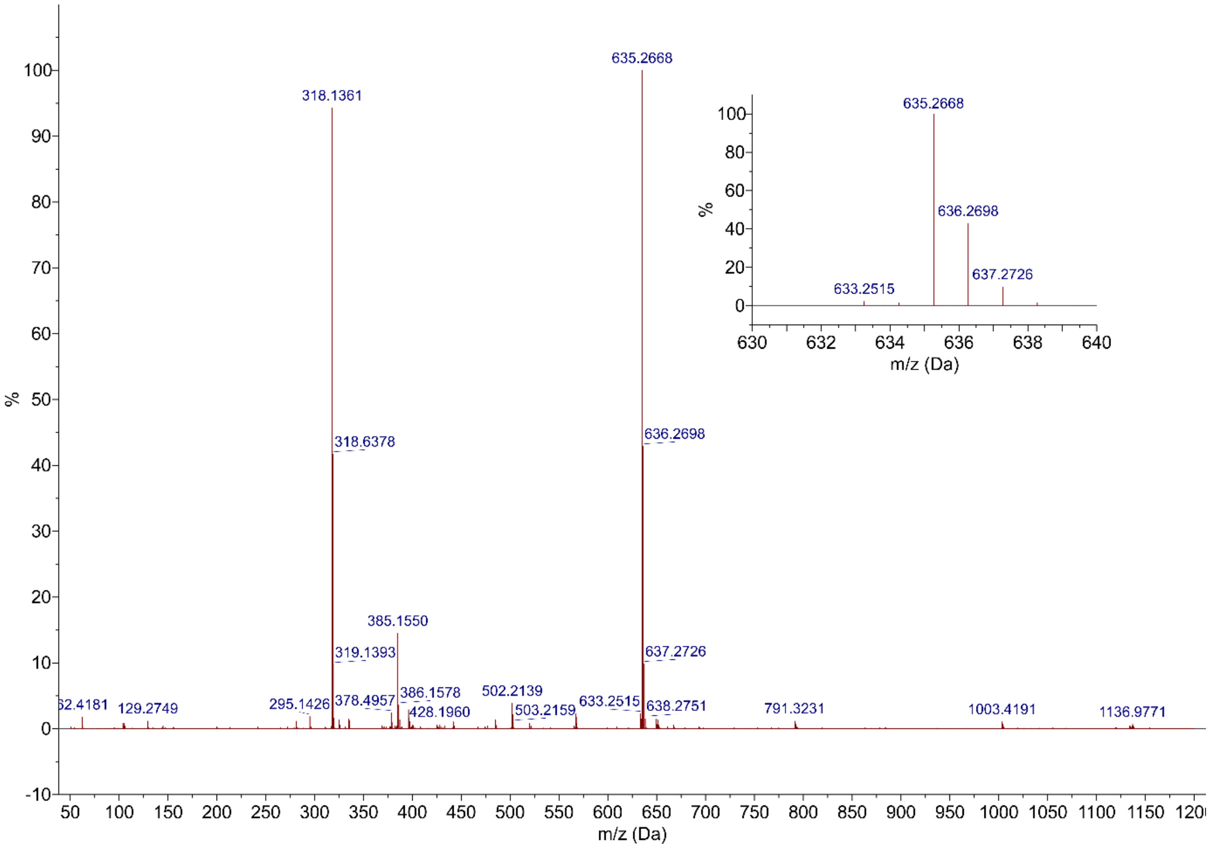

2 MS (ESI) *m/z*: calc. $[C_{38}H_{33}N_4O_4S]^+$ = 633.2497, found: 633.2504

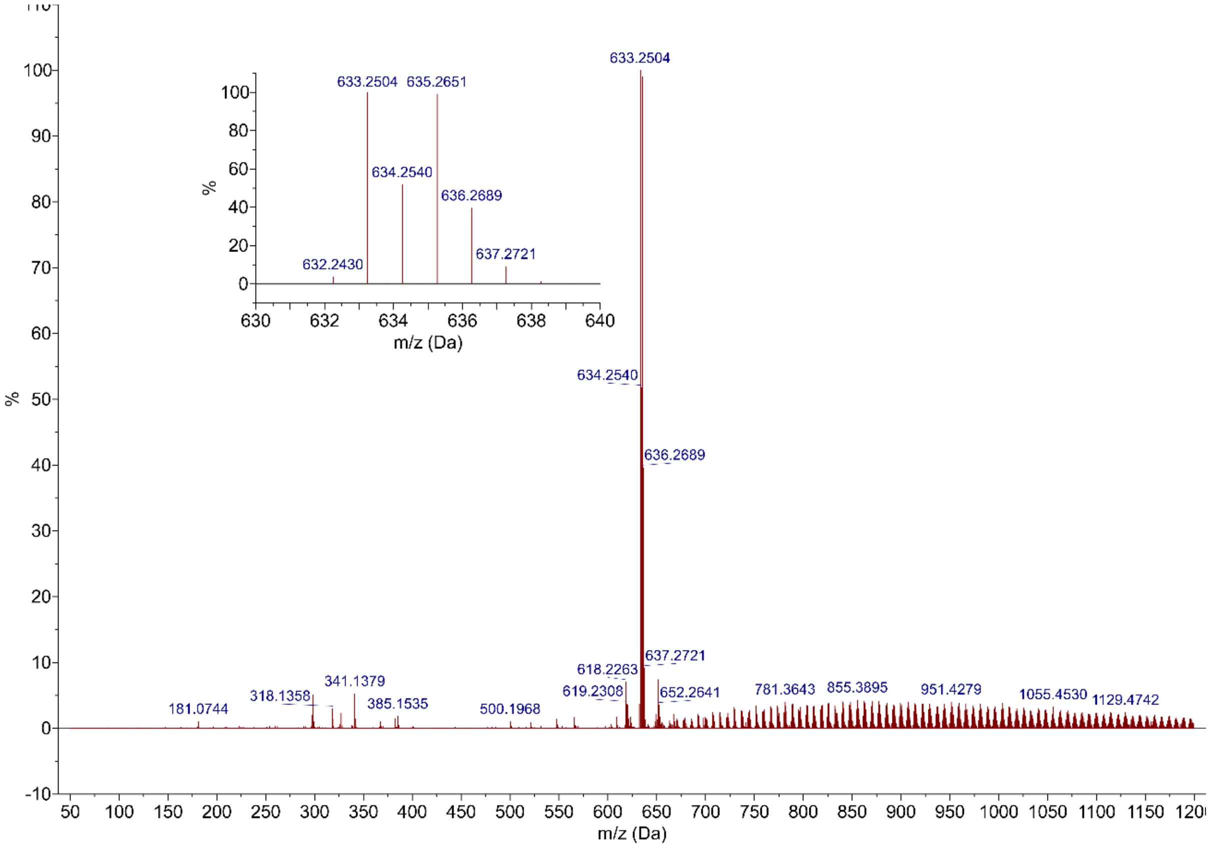

6 MS (ESI) *m/z*: calc. [$C_{16}H_{19}Br_2Si_2$] = 427.9450, found: 427.9446

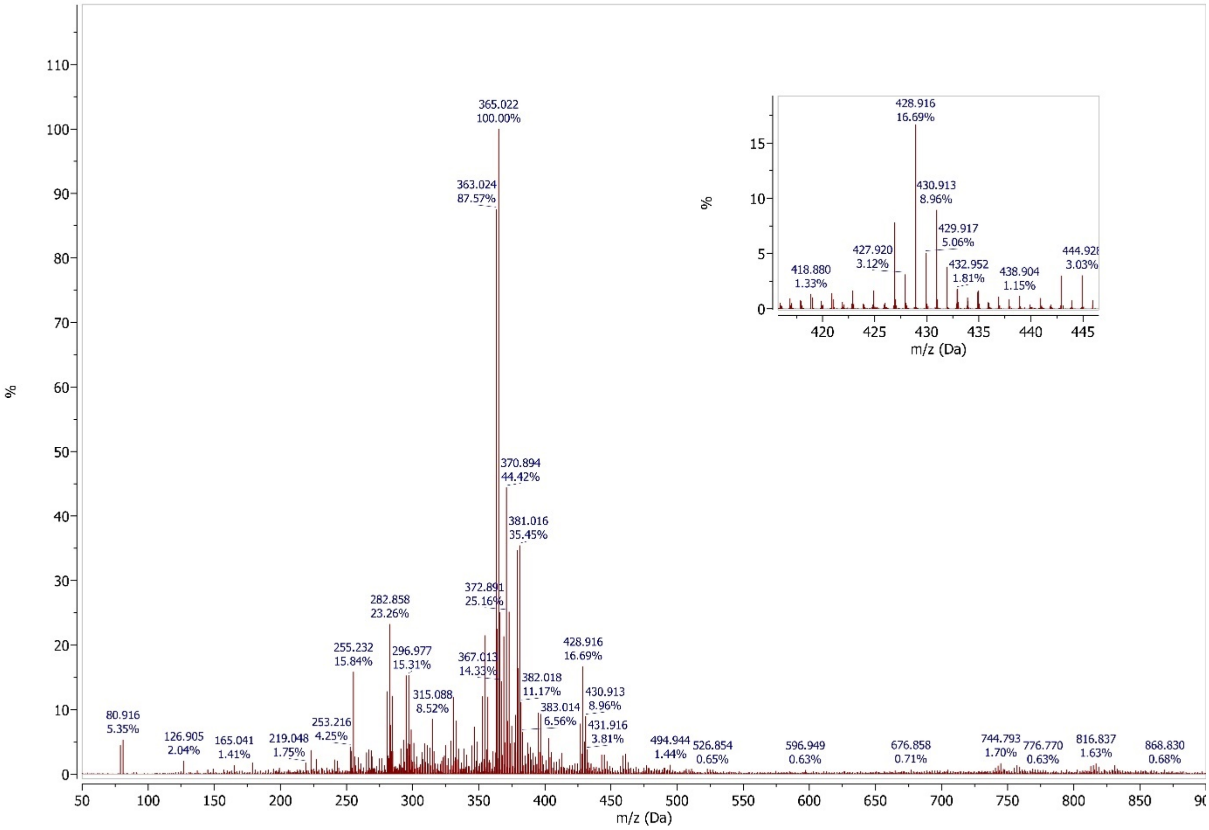

11a HRMS (EI) *m/z*: calc. [$C_{12}H_4Br_2F_6O_6S_2$] = 581.7700, found: 581.7275

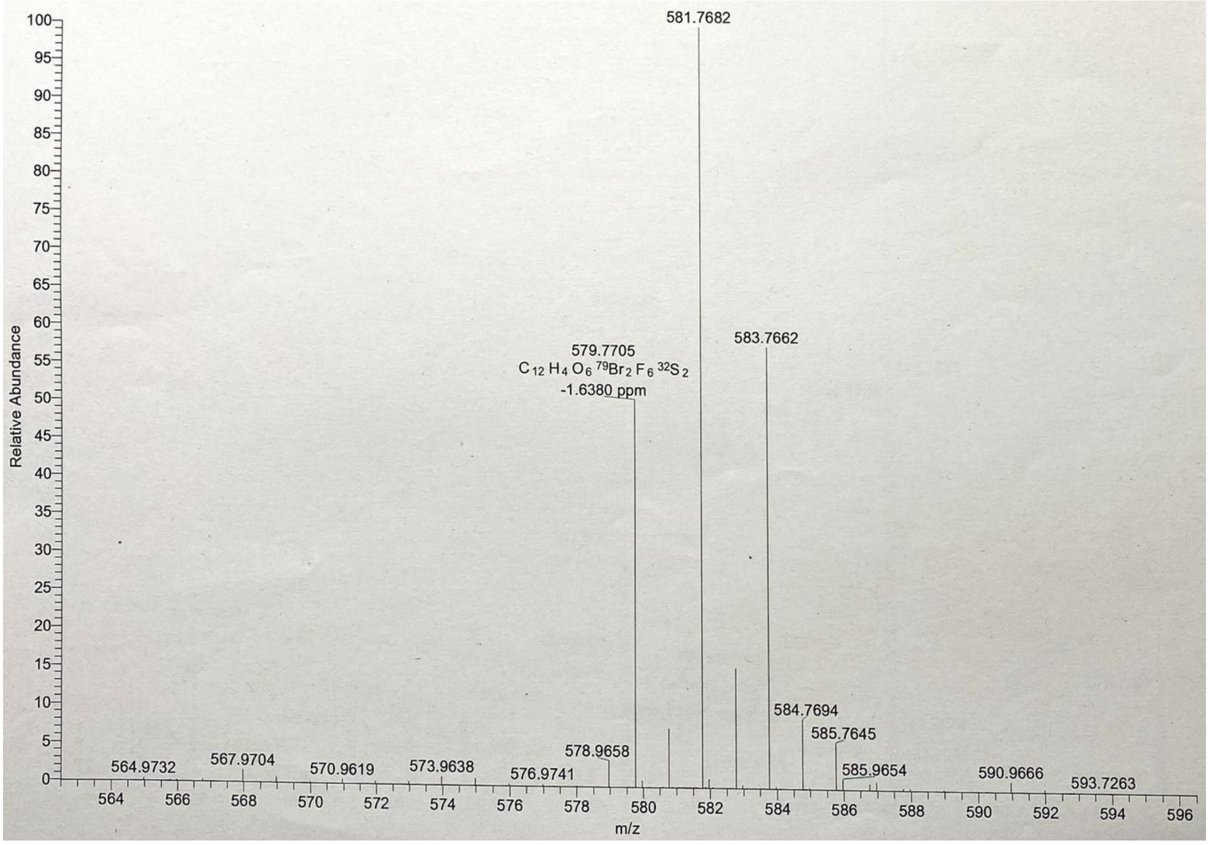

11b HRMS (EI) *m/z:* calc. [$C_{12}H_4Br_2F_6O_6S_2$] = 581.7700, found: 581.7690

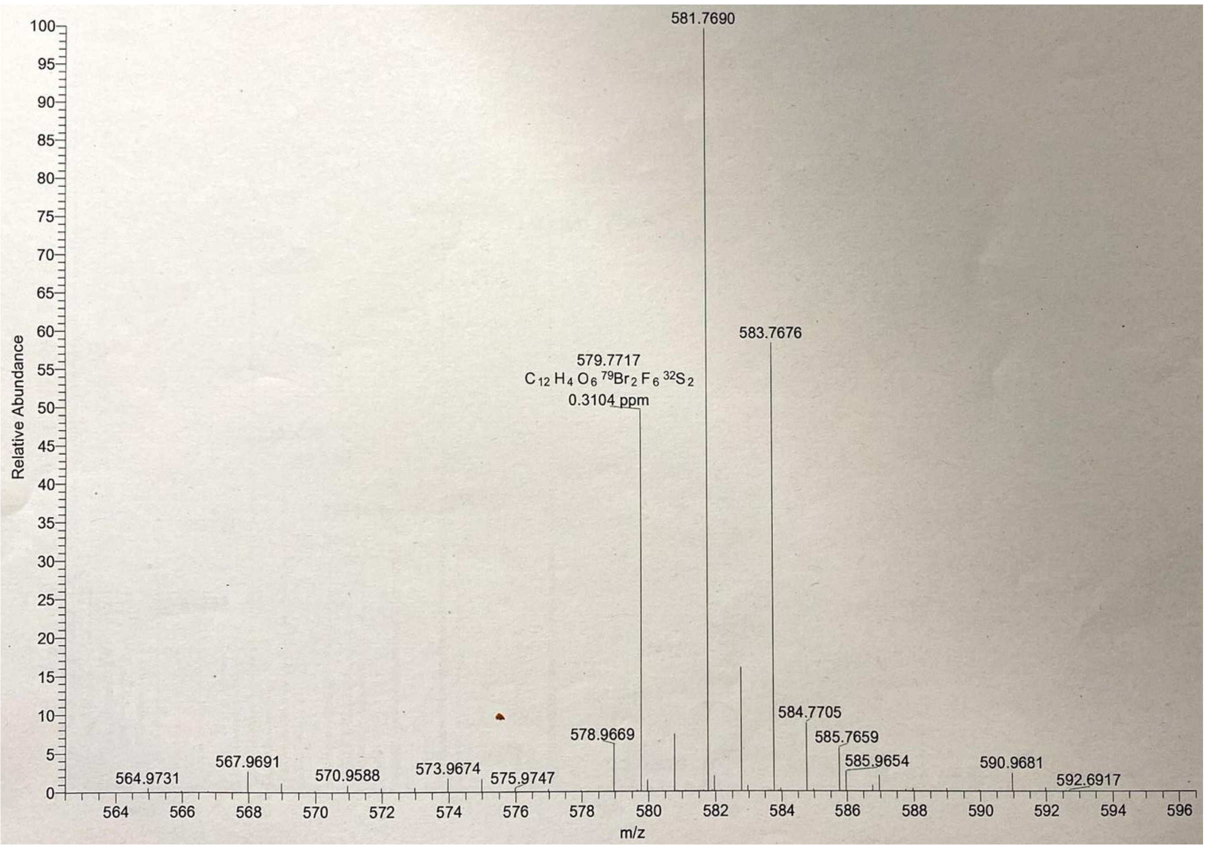

12a HRMS (EI) *m/z*: calc. [$C_{20}H_{22}Br_2Si_2$] = 477.9606, found: 477.9607

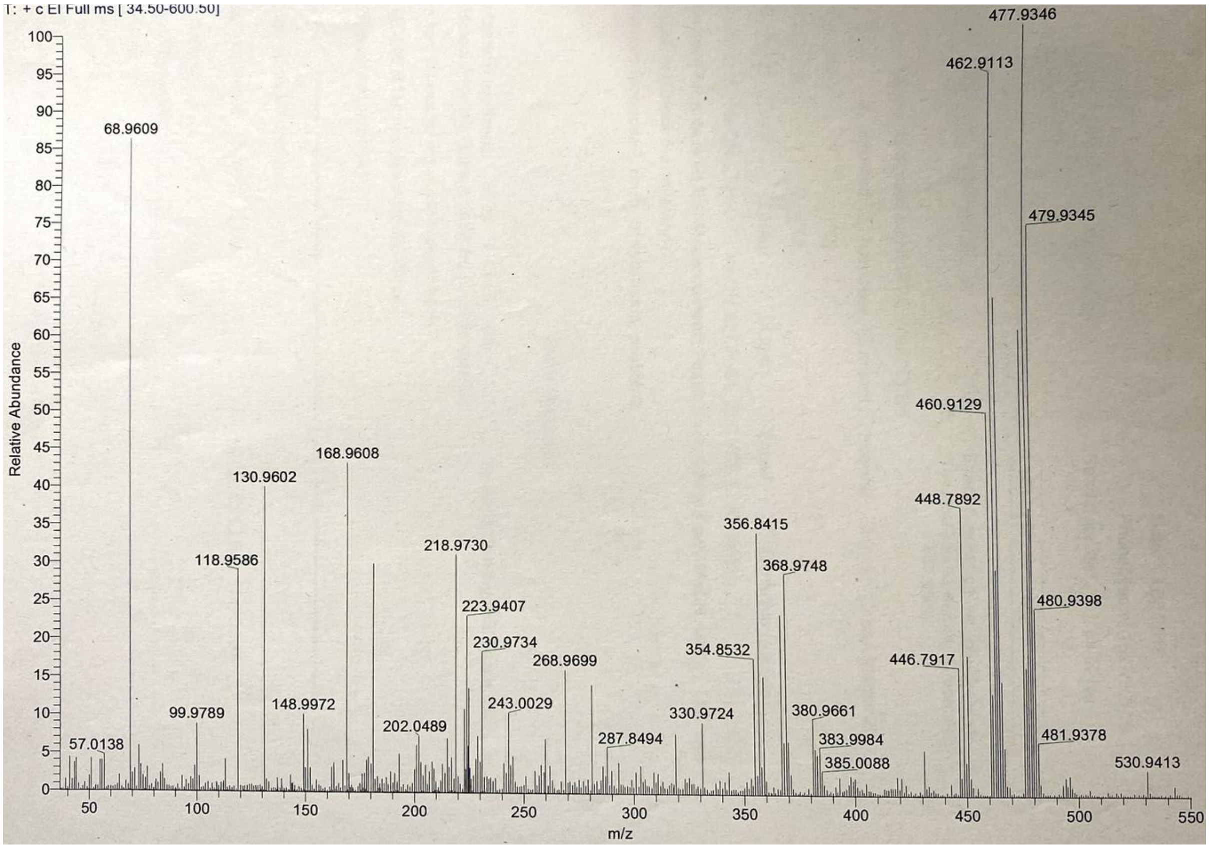

12b HRMS (EI) *m/z*: calc. [$C_{20}H_{22}Br_2Si_2$] = 477.9606, found: 477.9607

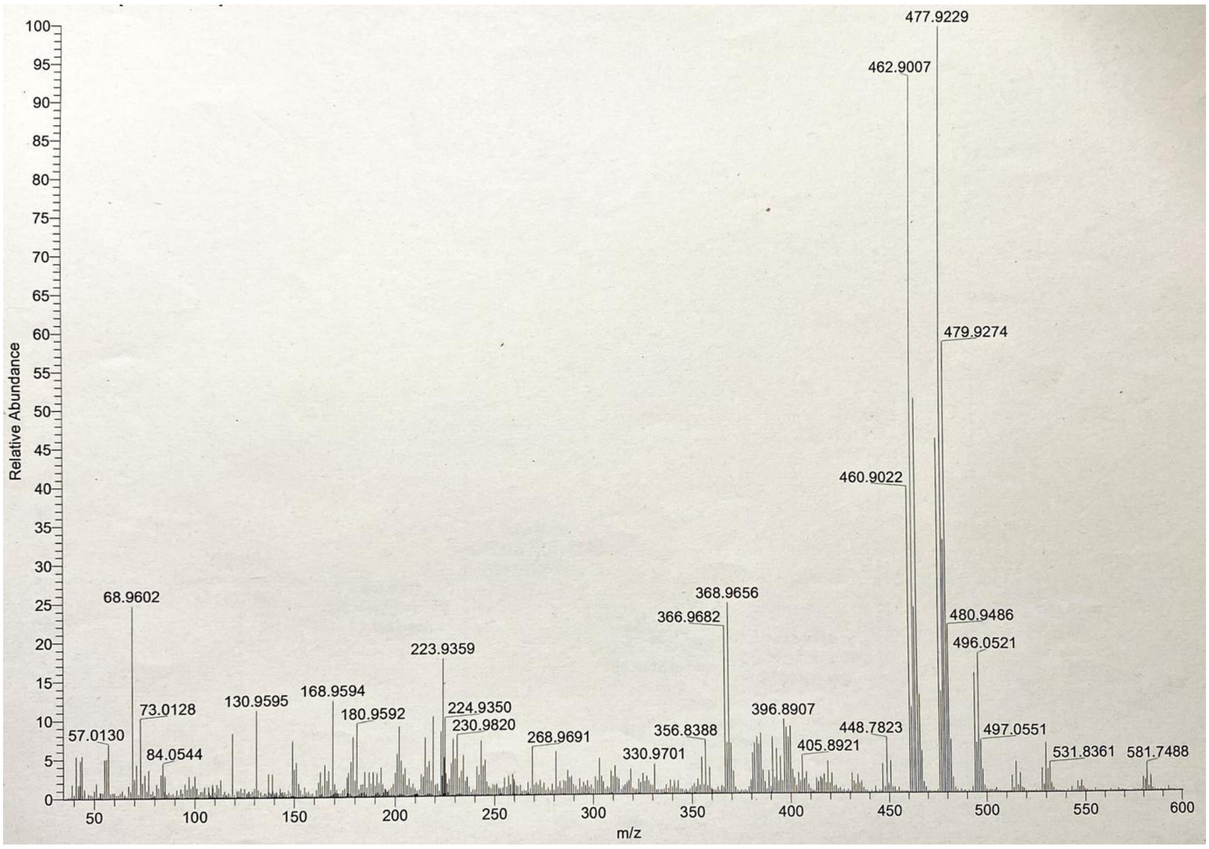

SI3 MS (ESI) *m/z*: calc. [$C_{17}H_{17}N_2O_2$]$^+$ = 281.1290, found: 281.1300

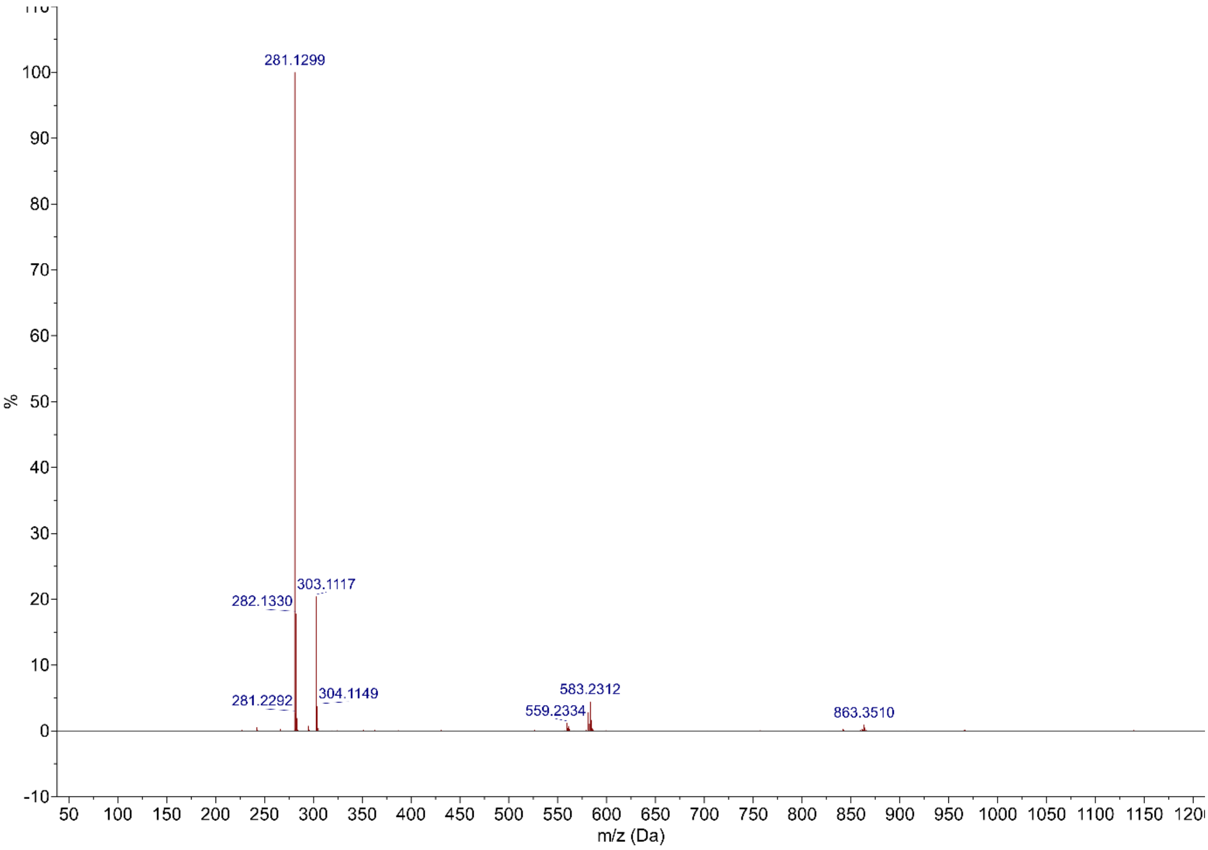

SI4 MS (ESI) *m/z*: calc. $[C_{23}H_{31s}N_2O_3Si]^+$ = 411.2104, found: 411.2128

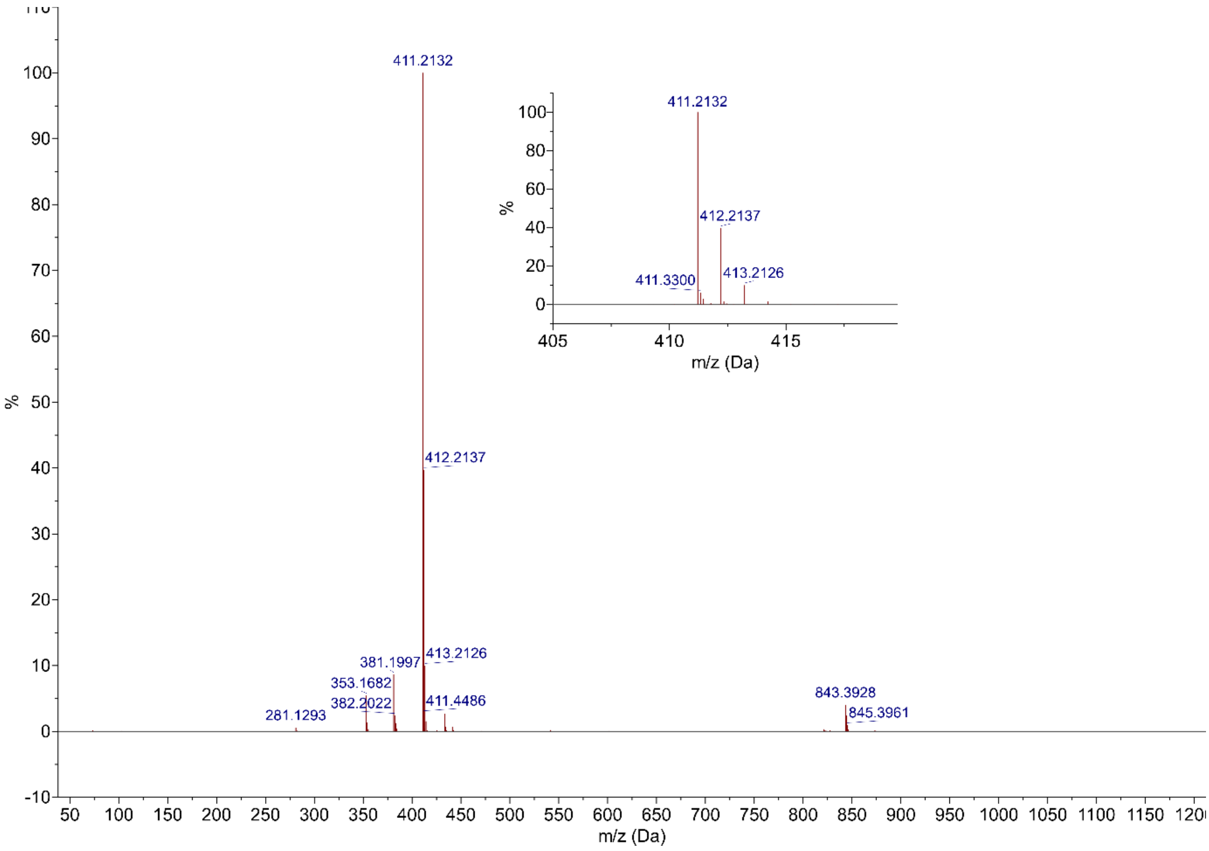

7 MS (ESI) *m/z*: calc. $[C_{23}H_{30}N_2O_3SiI]^+$ = 537.1070, found: 537.1051

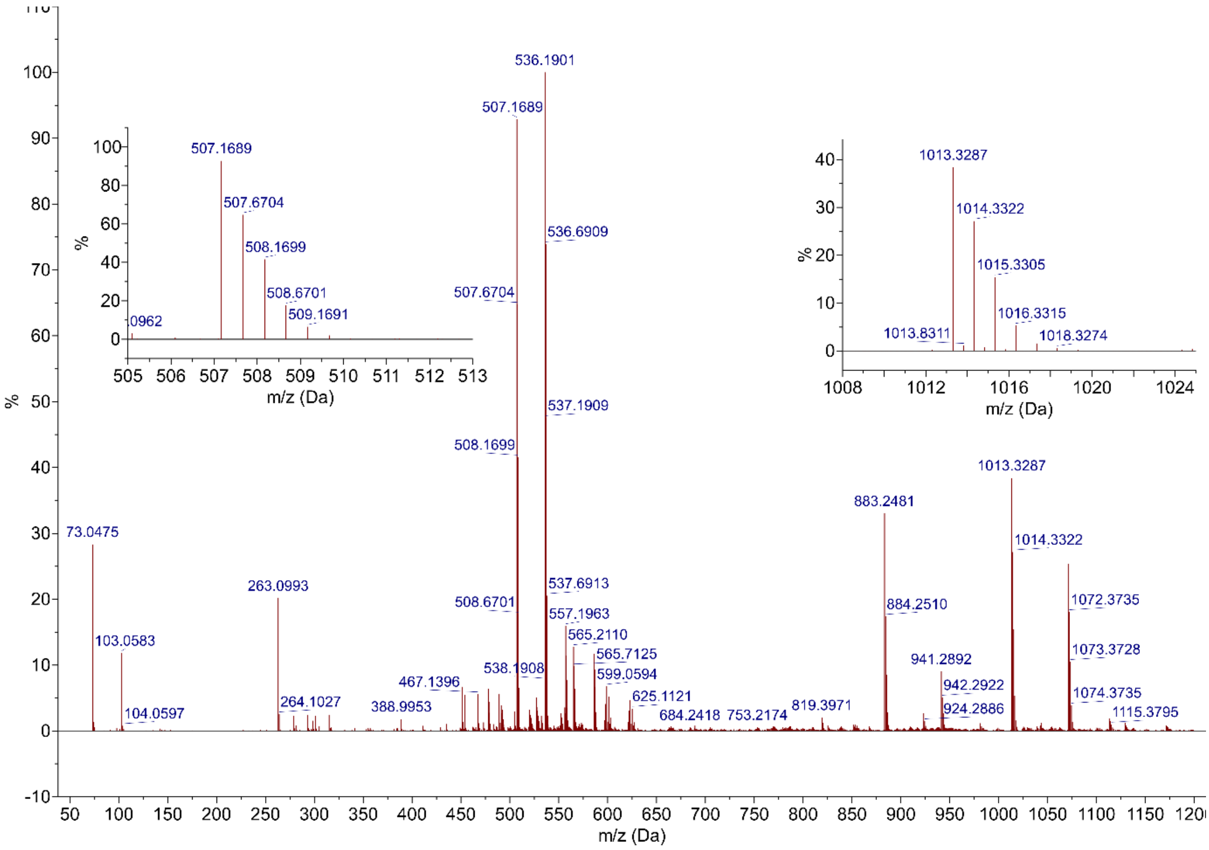

8 MS (ESI) *m/z*: calc. $[C_{56}H_{61}N_4O_6Si_2Br_2]^+$ = 1099.2496, found: 1101.2485

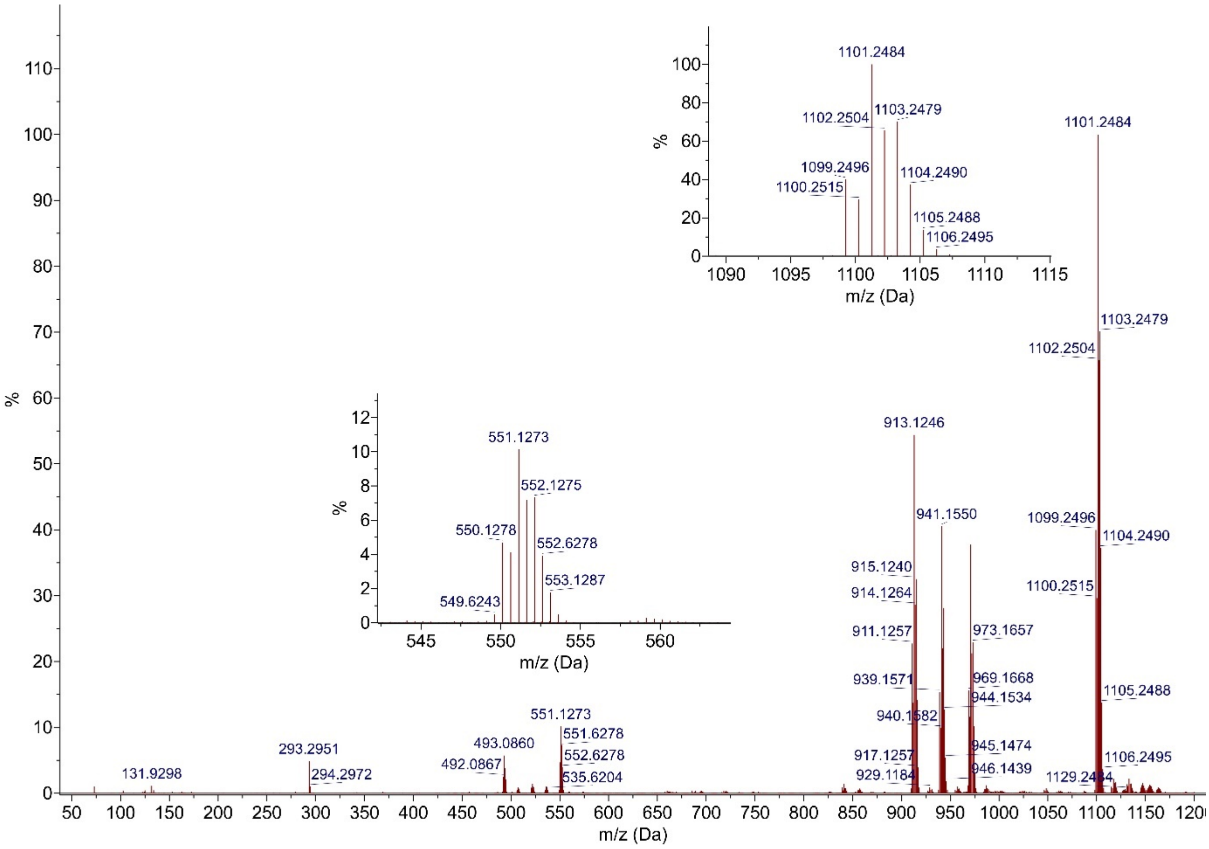

13a MS (ESI) *m/z*: calc. $[C_{60}H_6N_4O_6Si_2Br_2]^+$ = 1151.2627, found: 1151.2616

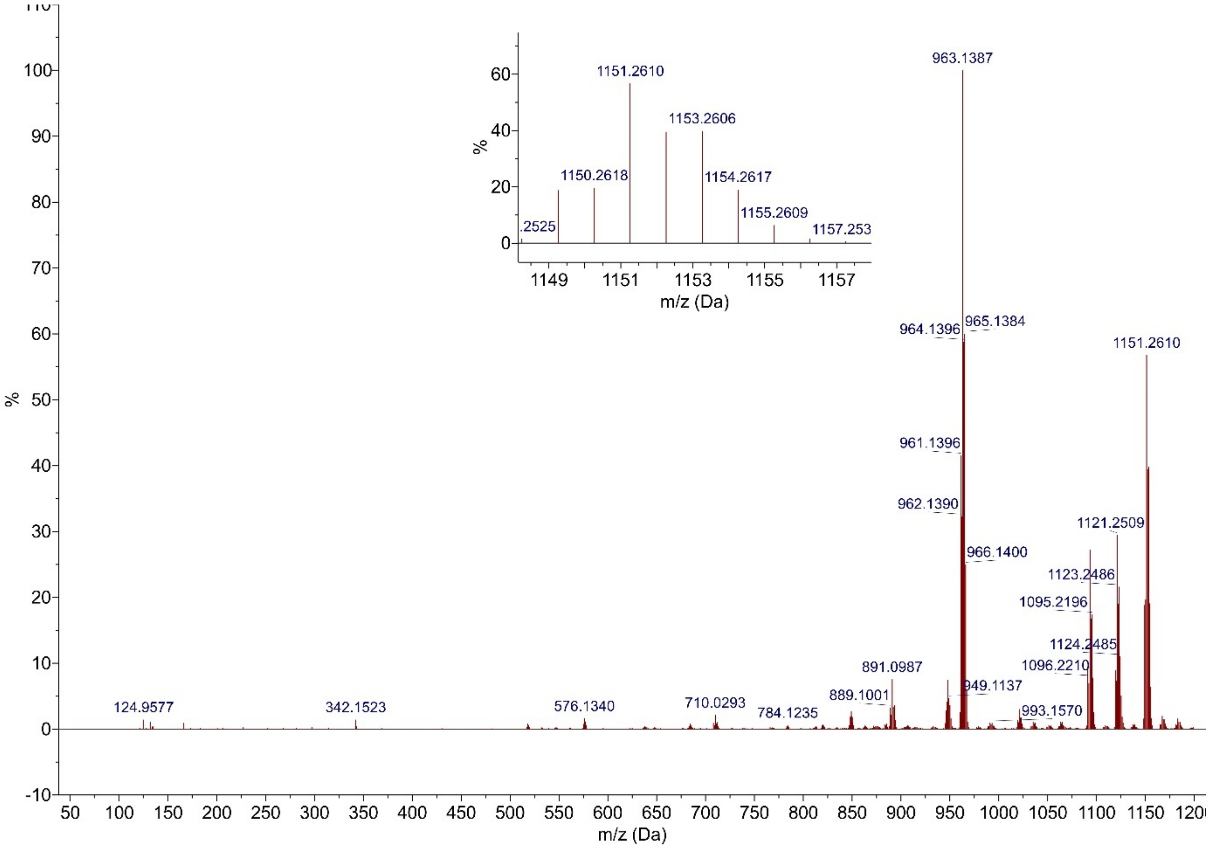

13b MS (ESI) *m/z*: calc. $[C_{60}H_{63}N_4O_6Si_2Br_2]^+$ = 1151.2627, found: 1151.2640

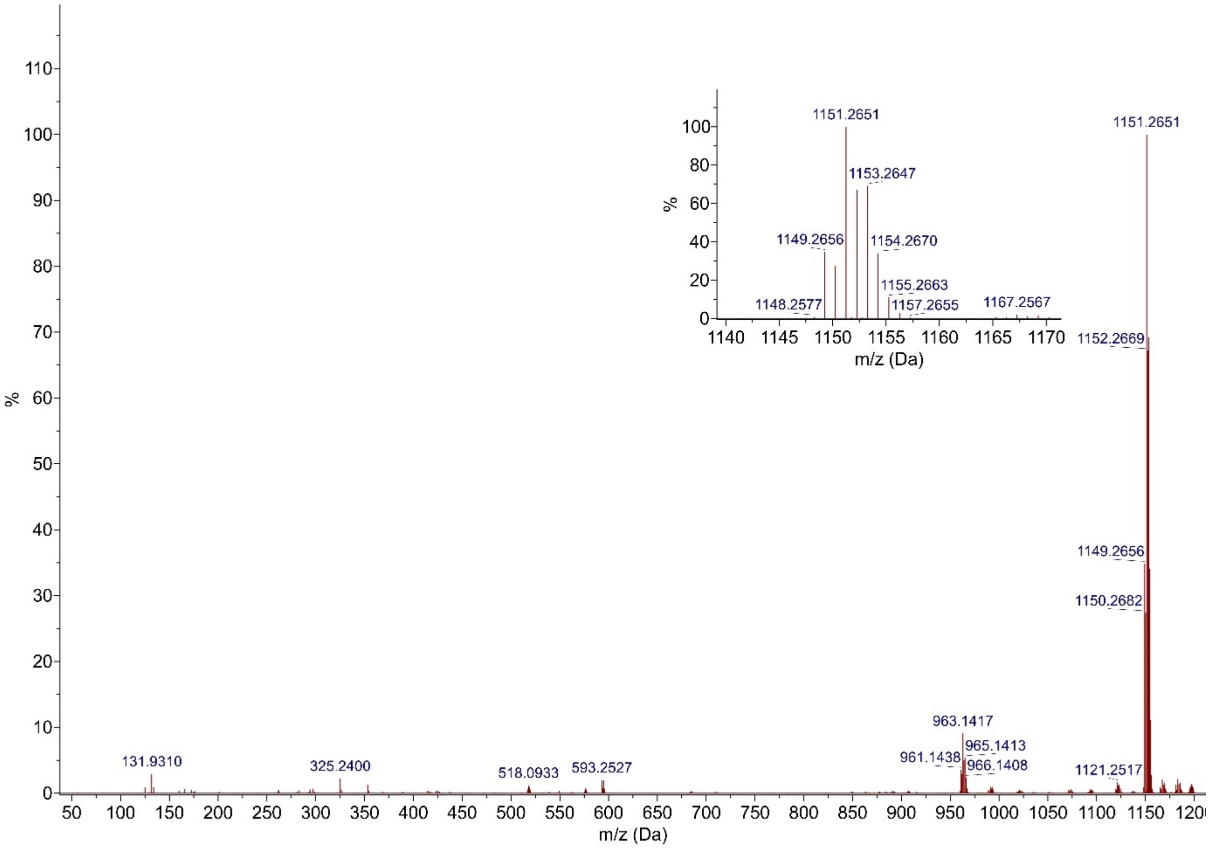

3 MS (ESI) *m/z*: calc. $[C_{56}H_{63}N_4O_6Si_2S_2]^+$ = 1007.3728, found: 1007.3740

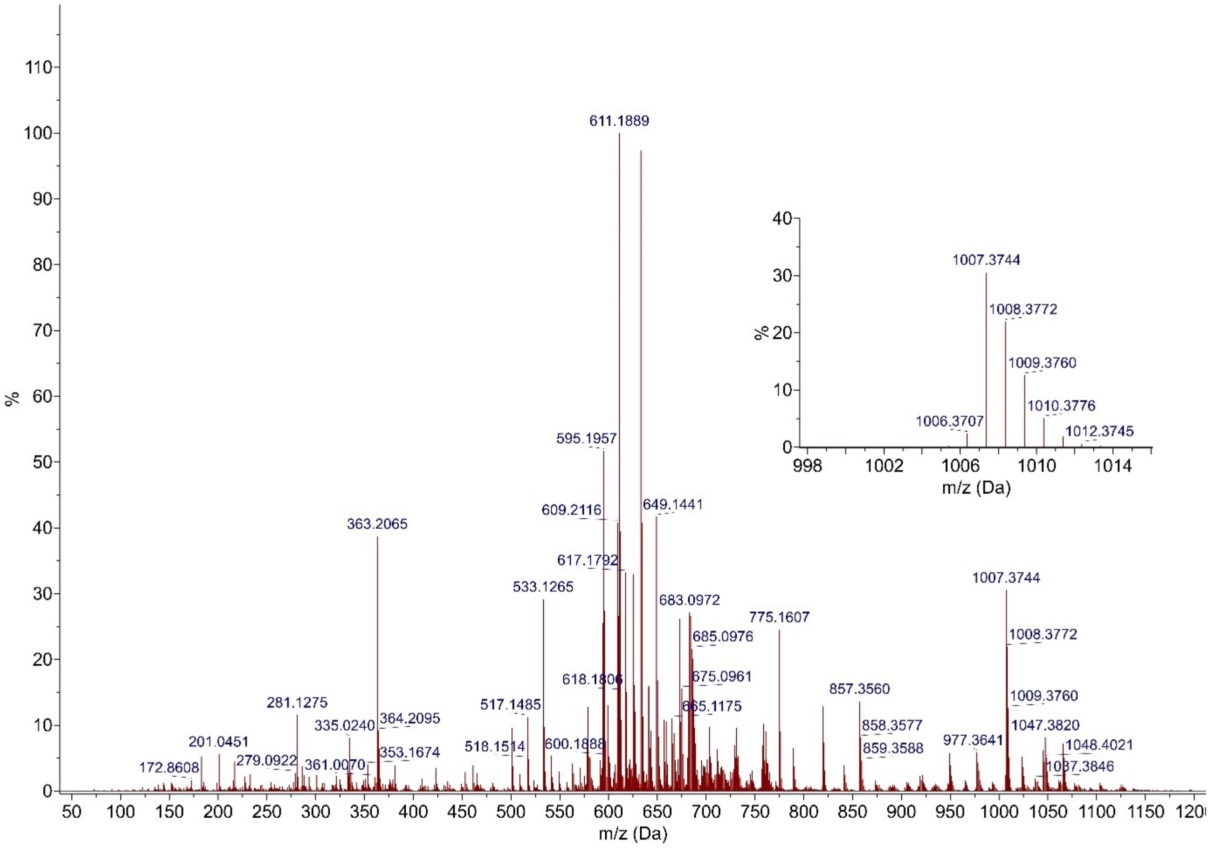

9a MS (ESI) *m/z*: calc. $[C_{60}H_{65}N_4O_6Si_2S_2]^+$ = 1057.3884, found: 1057.3887

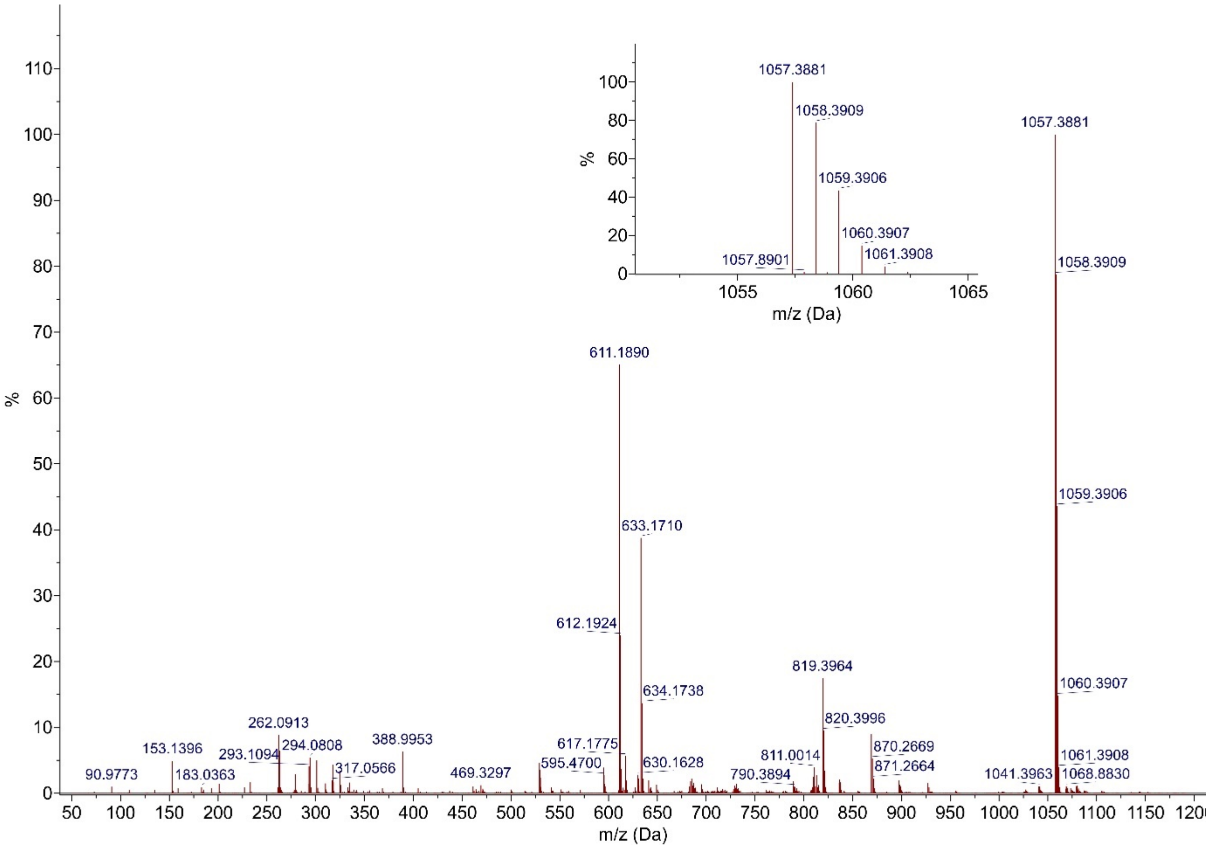

9b MS (ESI) *m/z*: calc. $[C_{60}H_{65}N_4O_6Si_2S_2]^+$ = 1057.3884, found: 1057.3907

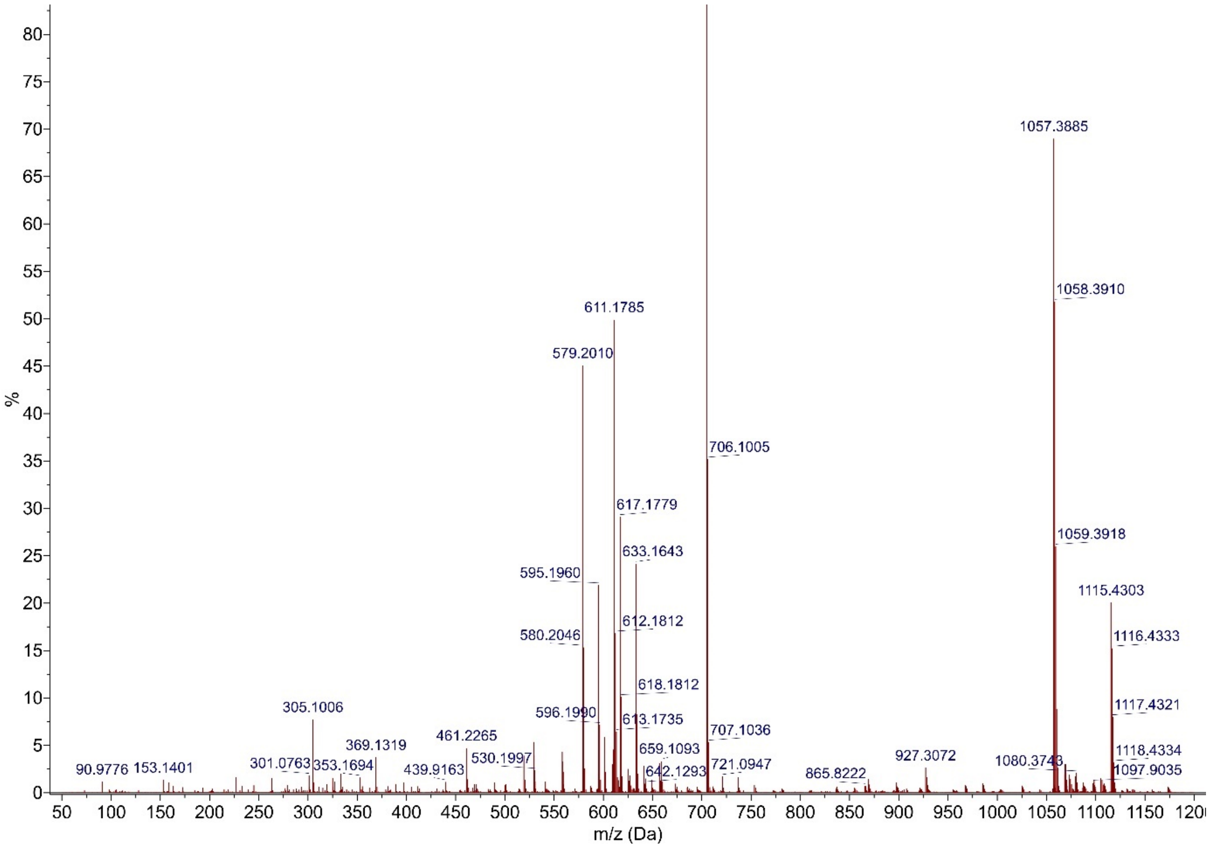

SI5 MS (ESI) *m/z*: calc. $[C_{44}H_{35}N_4O_4S_2]^+$ = 747.2100, found: 747.2120

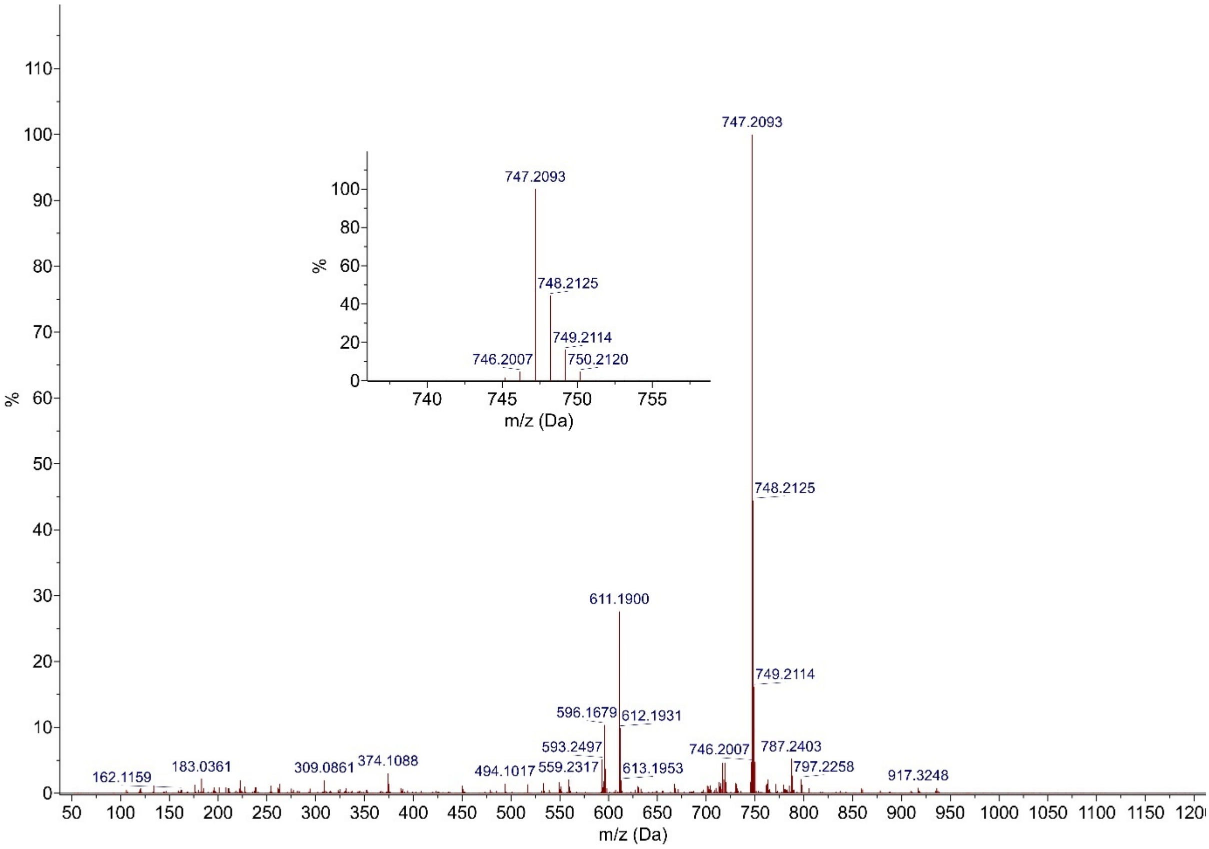

SI6 MS (ESI) *m/z*: calc. $[C_{48}H_{37}N_4O_4S_2]^+$ = 797.2256, found: 797.2294

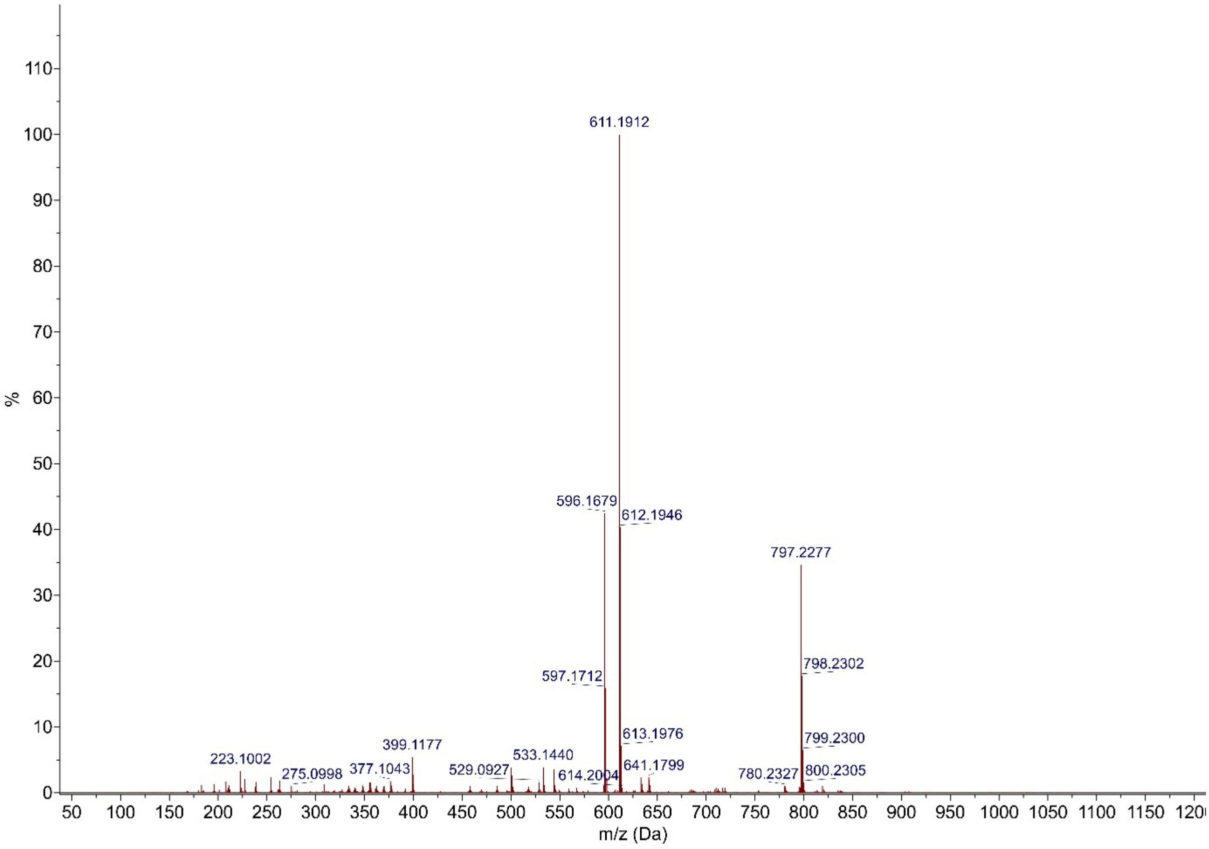

SI7 MS (ESI) *m/z*: calc. $[C_{48}H_{37}N_4O_4S_2]^+$ = 797.2256, found: 797.2267

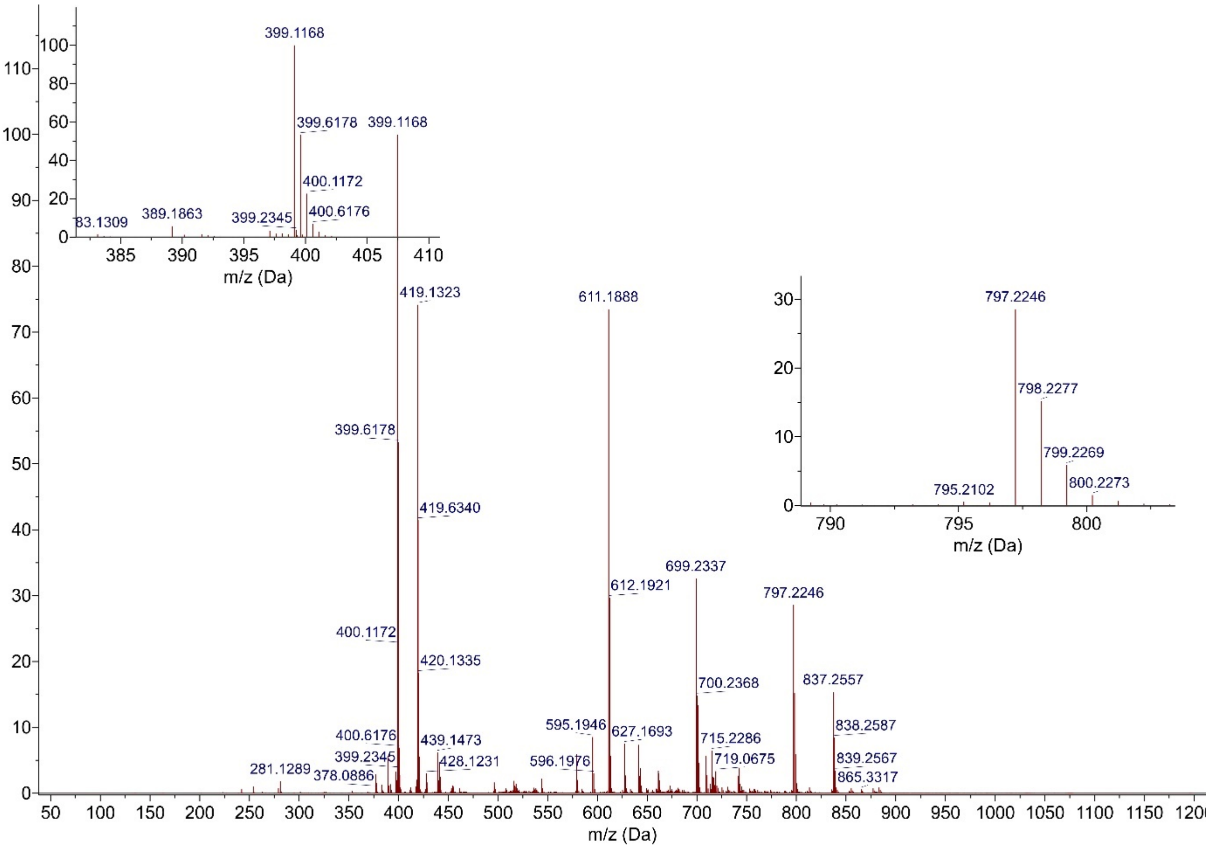

14 MS (ESI) *m/z*: calc. $[C_{44}H_{33}N_4O_4S_2]^+$ = 745.1943, found: 745.1965

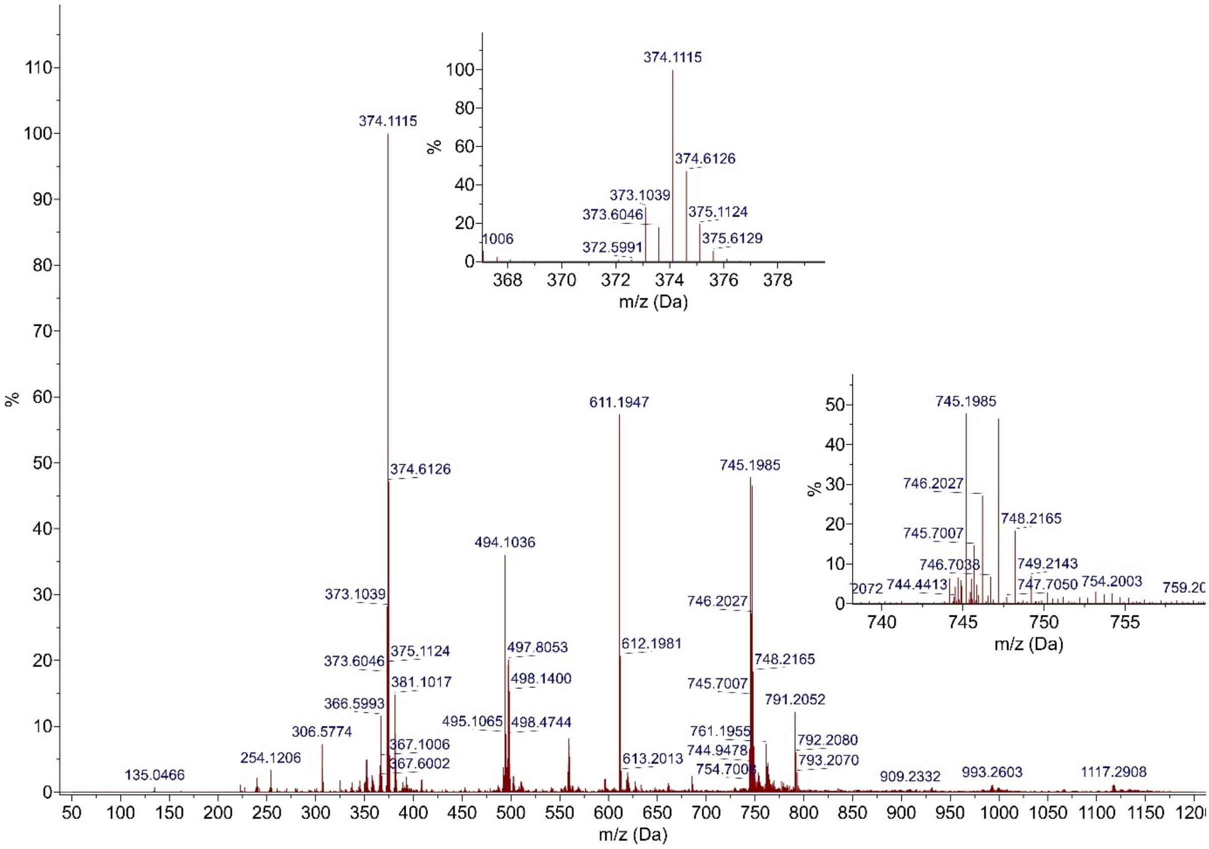

15a MS (ESI) *m/z*: calc. $[C_{48}H_{35}N_4O_4S_2]^+$ = 795.2100, found: 795.2155

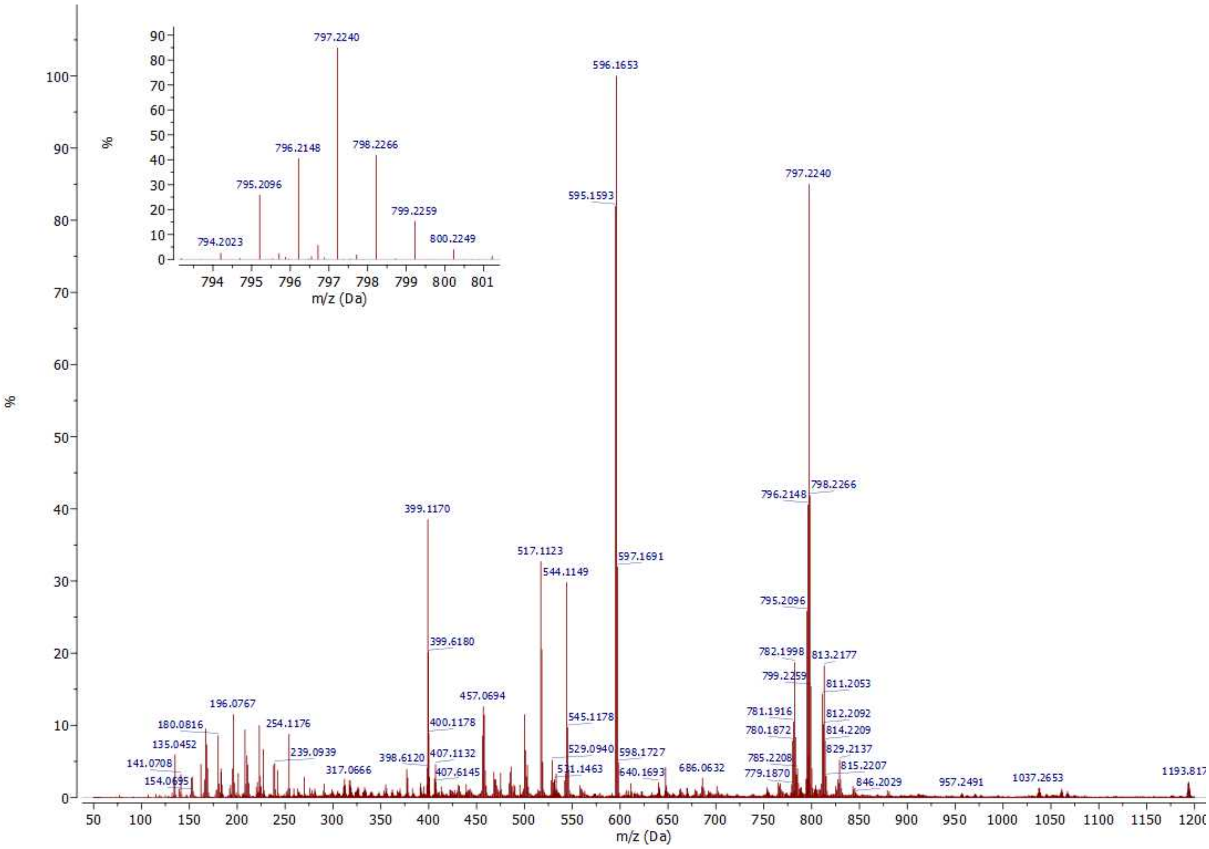

15b MS (ESI) *m/z*: calc. $[C_{48}H_{35}N_4O_4S_2]^+$ = 795.2100, found: 795.2097

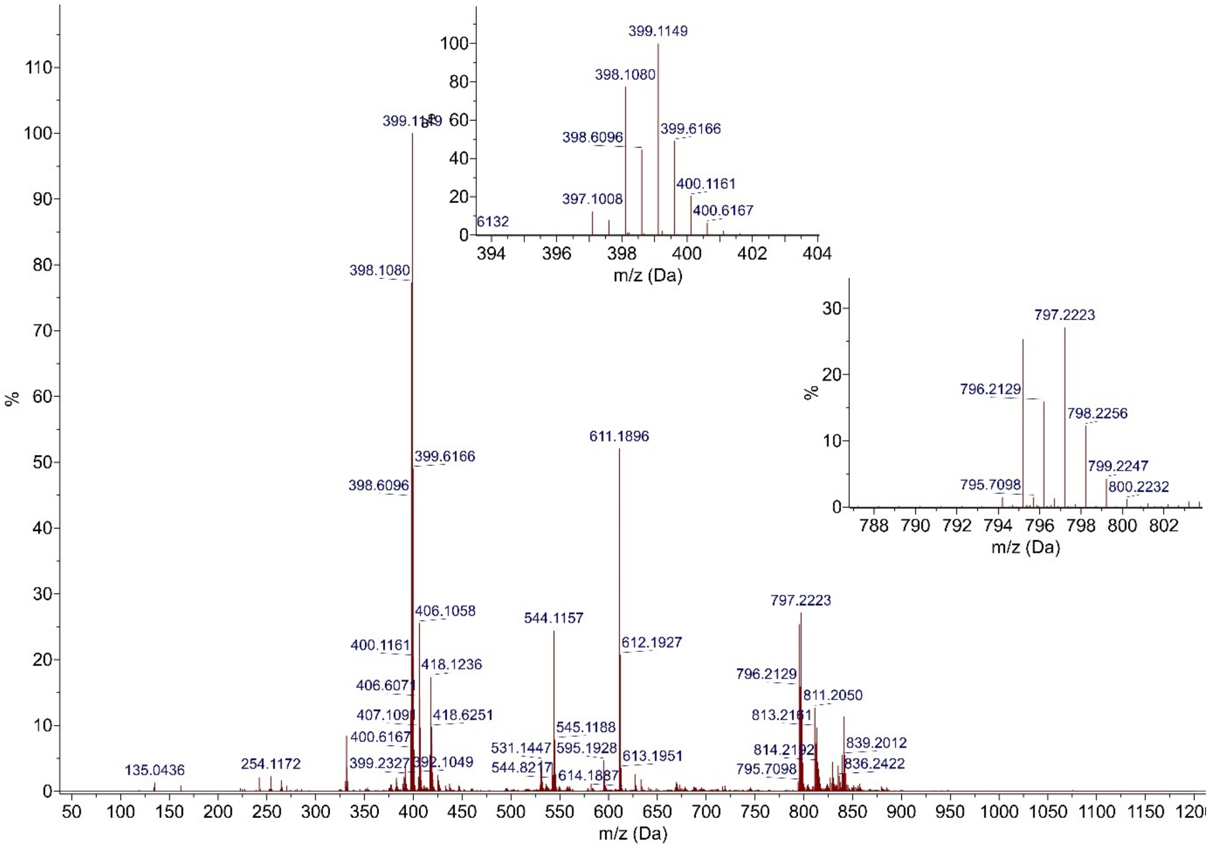

3.3. UV-Vis difference spectra

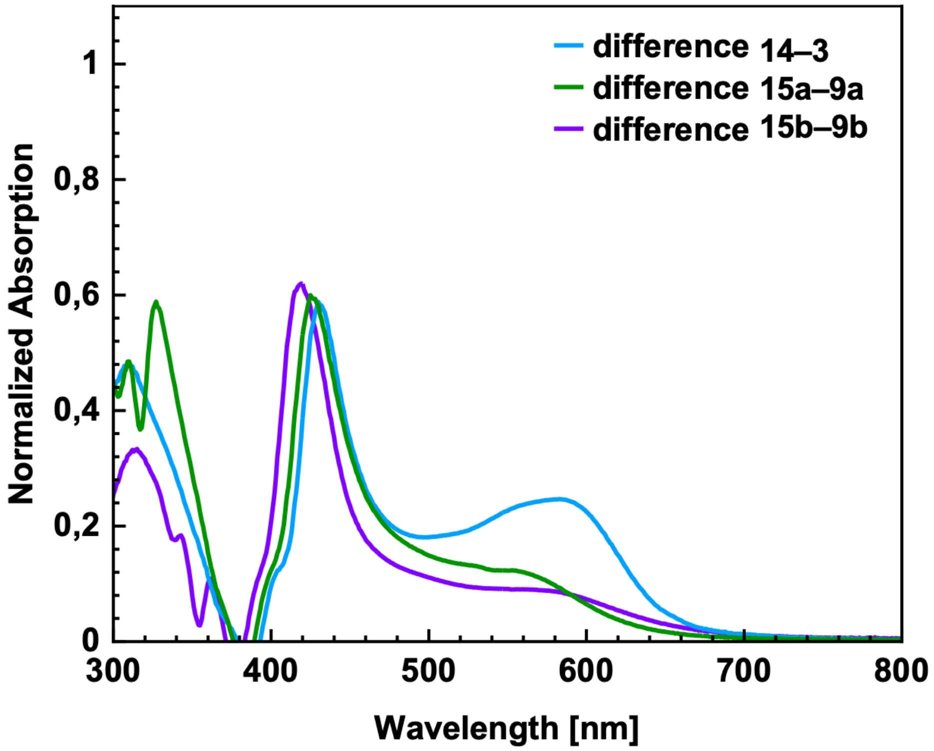

Figure S1. Difference spectra of **14**, **15a** and **15b** with their respective oxidation precursors **3**, **9a** and **9b**.

3.4. EPR measurements

EPR spectra were recorded using a Bruker EMXplus continuous wave (cw) X-Band spectrometer with nitrogen cooling for 100 K measurements. Samples were filled into fused silica glass tubes. Solvents were vacuum transferred to the samples and afterwards the sample tubes were sealed under vacuum. EPR spectra were analysed and simulated using the EasySpin MATLAB toolbox (Stoll, S. & Schweiger, A. EasySpin, a comprehensive software package for spectral simulation and analysis in EPR. J. Magn. Reson. 178, 42–55 (2006). MATLAB R2017a (9.2.0). The MathWorks (2017).)

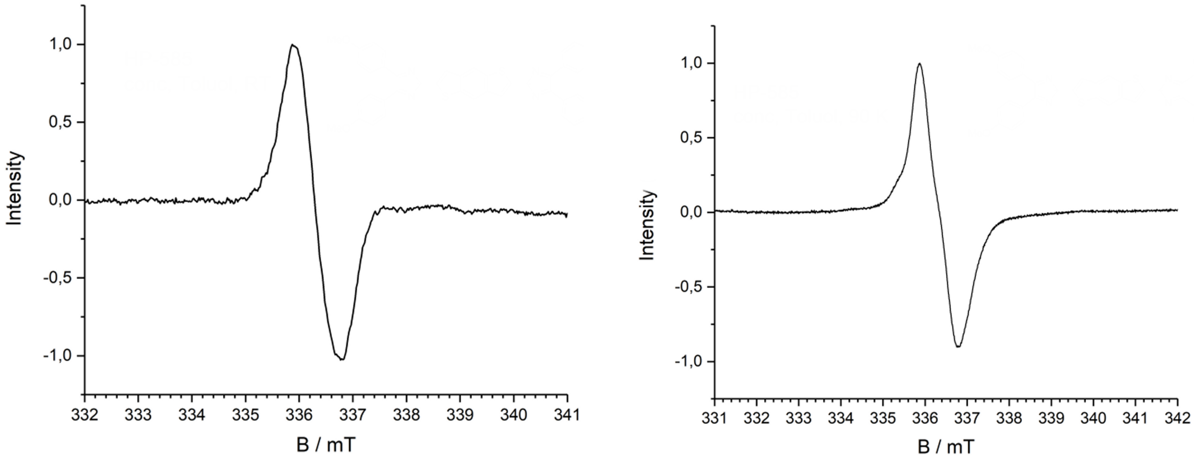

Figure S2. X-band EPR spectra of **14** (500µM in toluene) at 270 K (left) and 90 K (right)

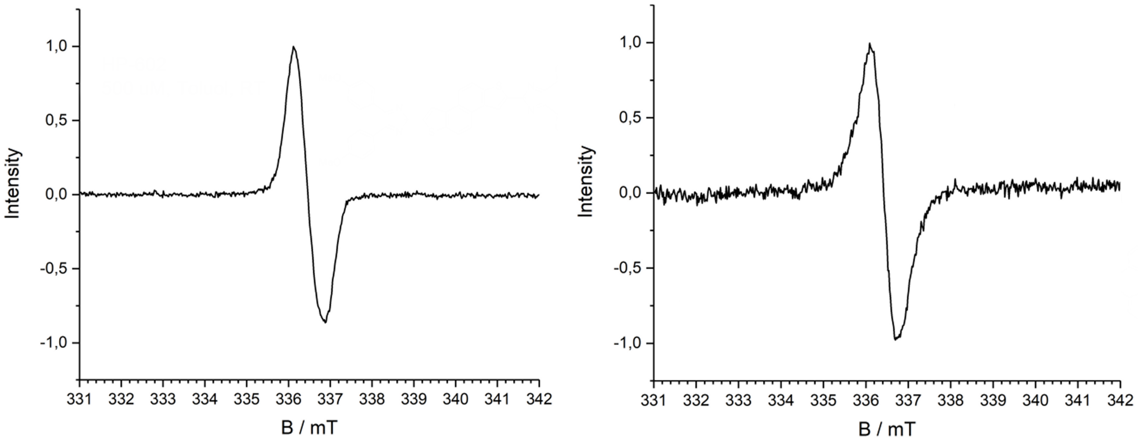

Figure S3. X-band EPR spectra of **15a** (500µM in toluene) at 270 K (left) and 90 K (right)

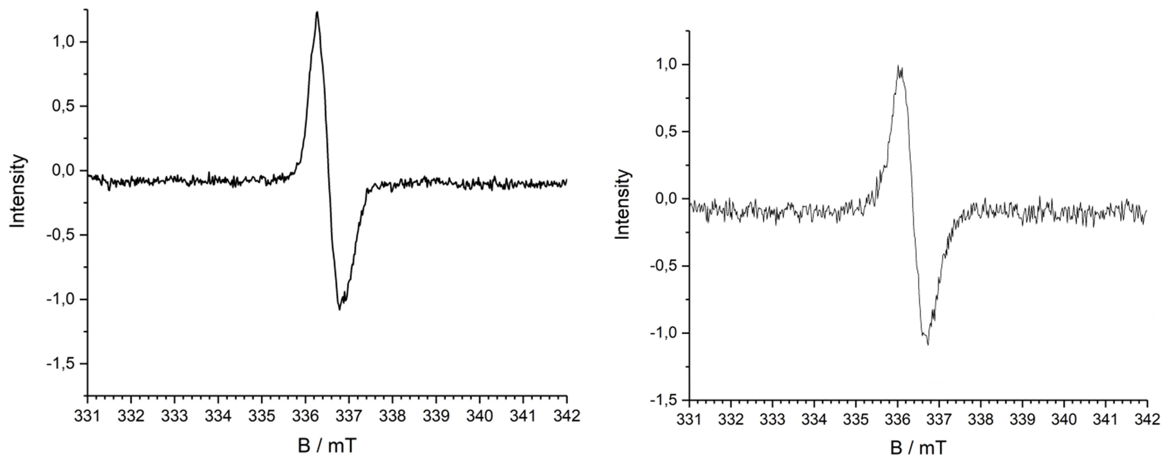

Figure S4. X-band EPR spectra of **15b** (500µM in toluene) at 270 K (left) and 90 K (right)

4. X-ray single crystal diffraction

X-ray diffraction data were collected using a Bruker Venture 8 three cycle diffractometer equipped with a Mo µ-source and a Photon III area detector at 120 K. Crystallographic details of the presented structure are given in the following table. The complete structure data information was deposited at the Cambridge Crystallographic Data Centre (CCDC) under deposition numbers 2214715 (**11b**), 2214716, (**12b**) 2214717 (**13a**), 2214718 (**11a**) and 2221938 (**6**).

Table S1. Overview over the crystallographic details of the presented structures.

	**6**	**11a**	**11b**	**12b**	**13a**
Chemical formula	$C_{16}H_{20}Br_2Si_2$	$C_{12}H_4Br_2S_2O_6F_6$	$C_{12}H_4Br_2S_2O_6F_6$	$C_{20}H_{22}Si_2Br_2$	$C_{60}H_{62}N_4O_6Si_2Br_2$
M_r	428.32	582.09	582.09	478.37	1151.13
Crystal system	Monoclinic	Triclinic	Triclinic	Orthorhombic	triclinic
Space group	$P2_1/c$	P-1	P-1	P2(1)2(1)2(1)	P-1
a (Å)	6.2112(2)	6.037(4)	5.375(1)	6.578(1)	6.639(1)
b (Å)	32.4877(13)	7.298(5)	8.601(1)	17.997(3)	10.112(1)
c (Å)	9.9388(4)	10.008(7)	9.882(2)	18.308(3)	22.165(2)
α (°)	90	75.544(8)	112.526(3)	90	95.765(2)
β (°)	107.660(2)	85.734(8)	92.069(3)	90	90.401(2)
γ (°)	90	85.660(9)	93.633(4)	90	93.682(2)
V ($Å^3$)	1911.01(13)	425.0(5)	420.3(1)	2167.5(6)	1477.3(3)
Z	4	1	1	4	1
Densitiy (g cm^{-3})	1.489	2.274	2.300	1.466	1.294
F(000)	856	280	280	960	596
Radiation Type	MoKα	MoKα	MoKα	MoKα	MoKα
µ (mm^{-1})	4.357	5.107	5.165	3.850	1.464
Crystal size	0.25x0.18x0.16	0.35x0.39x0.27	0.45x0.15x0.15	0.45x0.43x0.37	0.32x0.31x0.27
Meas. Refl.	51801	3839	3351	12922	18045
Indep. Refl.	4755	1591	1694	4130	6079
Obsvd. [$I > 2\sigma(I)$]	4392	1381	1598	3504	5195
R_{int}	0.0728	0.0401	0.033	0.0638	0.0311
R [$F^2 > 2\sigma(F^2)$]	0.0316	0.0318	0.0284	0.0357	0.0368
wR(F^2)	0.0678	0.0798	0.0736	0.0708	0.1126
S	1.130	1.054	1.163	1.007	1.114
$\Delta\rho_{max}$	0.585	0.654	0.526	0.665	0.403
$\Delta\rho_{min}$	-0.656	-0.733	-0.929	-0.447	-0.306
CCDC	2221938	2214718	2214715	2214716	2214717

5. Computational details

Computational experiments were performed with the ORCA 5.0.3 package.[SI3] Geometry optimizations were performed with the PBE0[SI4] functional and the def2-TZVP[SI5] basis set. The molecular structures were optimized as closed-shell (RKS), open-shell singlet (UKS using broken-symmetry formalism) and open-shell triplet (UKS). TD-DFT calculations were performed on this level of theory with the optimized geometries using CPCM solvation model for dichloromethane (SMD). The equilibrium structure was confirmed by frequency calculation on the same level of theory. Complete active space calculations with two electrons in two orbitals (CASSCF(2,2)) were performed with the broken-symmetry optimized geometries. Fractional occupation number weighted electron density (FOD)[SI6][SI7] analysis was performed on the broken-symmetry optimized geometries as implemented in the ORCA 5.0.3 package. The molecular coordinates can be found in the provided xyz-file.

Table S1: Summarized results of the TD-DFT calculations (PBE0/def2-TSVP, CPCM/SMD (CH_2Cl_2)

compound	RKS			UKS singlet			UKS triplet		
	[eV]	f	contribution	[eV]	f	contribution	[eV]	f	contribution
**14**	1.536	6.078	92% H –> L	1.601	1.287	59% H-1 –>L	1.094	0.396	94% H –> L
				1.731	0.842	60% H-1 –>L	1.628	0.272	53% H-1 –> L
				2.002	0.158	88% H-2 –>L	1.851	0.789	93% H-3 –> L
							2.387	0.963	67% H –> L
**15a**	1.284	6.810	92% H –> L	1.382	0.633	94% H –> L	1.678	0.842	88% H –> L
				1.832	0.386	75% H-1 –> L	1.848	0.314	58% H-1 –> L
				1.900	0.378	78% H-2 –> L	2.001	0.210	68% H-3 –> L
				2.164	0.162	53% H-4 –> L	2.453	0.141	63% H-5 –> L
**15b**	1.431	7.932	91% H –> L	1.440	1.423	92% H –> L	1.697	0.728	88% H –> L
				1.870	0.173	35% H-1 –> L	1.777	0.493	79% H-1 –> L
				1.955		35% H-2 –> L	1.858	0.237	62% H-3 –> L
						35% H-3 –> L			
					0.343	57% H –> L			

6. References

[SI1] a) G. M. Sheldrick *Acta Cryst. A71*, **2015**, 3-8.; G. M. Sheldrick *Acta Cryst. C* C71 **2015**, 3-8.

[SI2] P. Hou, S. Peschtrich, N. Huber, W. Feuerstein, A. Bihlmeier, I. Krummenacher, R. Schoch, W. Klopper, F. Breher, J. Paradies, *Chemistry – A European Journal* **2022**, *28*, e202200478.

[SI3] F. Neese, F. Wennmohs, U. Becker, C. Riplinger, *J. Chem. Phys.* **2020**, *152*, 224108.

[SI4] J. M. del Campo, J. L. Gázquez, S. B. Trickey, A. Vela, *J. Chem. Phys.* **2012**, *136*, 104108.

[SI5] F. Weigend, R. Ahlrichs, *Phys. Chem. Chem. Phys.* **2005**, *7*, 3297–3305.

[SI6] C. A. Bauer, A. Hansen, S. Grimme, *Chemistry – A European Journal* **2017**, *23*, 6150–6164.

[SI7] S. Grimme, A. Hansen, *Angewandte Chemie International Edition* **2015**, *54*, 12308–12313.
